# Building up libraries and production line for single atom catalysts with precursor-atomization strategy

**DOI:** 10.1038/s41467-022-33442-2

**Published:** 2022-09-29

**Authors:** Xiaohui He, Hao Zhang, Xingcong Zhang, Ying Zhang, Qian He, Hongyu Chen, Yujie Cheng, Mi Peng, Xuetao Qin, Hongbing Ji, Ding Ma

**Affiliations:** 1 Fine Chemical Industry Research Institute, School of Chemistry, Sun Yat-sen University, 510275 Guangzhou, China; 2 Beijing National Laboratory for Molecular Sciences, College of Chemistry and Molecular Engineering and College of Engineering, and BIC-ESAT, Peking University, 100871 Beijing, China; 3 Huizhou Research Institute, Sun Yat-sen University, 516081 Huizhou, China

**Keywords:** Catalyst synthesis, Heterogeneous catalysis, Synthesis and processing

## Abstract

Having the excellent catalytic performance, single atom catalysts (SACs) arouse extensive research interest. However, the application of SACs is hindered by the lack of versatile and scalable preparation approaches. Here, we show a precursor-atomization strategy to produce SACs, involving the spray of droplets of solutions containing metal precursors onto support surface through ultrasonic atomization and the subsequent calcination. This approach is versatile to successful synthesis of a series of catalysts, including 19 SACs with different metal sites and supports and 3 derivatives of SACs (single atom alloys, double atom catalysts and bi-metallic SACs). Furthermore, it can be scaled up by a homemade production line with productivity over 1 kg day^−1^, and the well-controlled catalyst uniformity is evidenced by the identical characterization results and catalytic properties in Suzuki-Miyaura cross-coupling. This strategy lays a foundation for further investigation and may accelerate the trend from basic research to industrial applications of SACs.

## Abstract (web summary)

The application of single atom catalysts (SACs) is hindered by the lack of versatile and scalable preparation approaches. Here the authors report a precursor-atomization strategy that can be scaled up with productivity over 1 kg/day for the synthesis of a series of SACs and derivatives.

## Introduction

Single-atom catalysts (SACs), featured with spatially isolated metal atoms on underlying supports, are regarded as a class of promising materials^1^, because they integrate the merits of homogeneous catalysts (high atom utilization, activity, and selectivity) and heterogeneous ones (good stability and reusability)^2^, and show superior catalytic performance in practical application, including high-value chemicals production^3^, energy conversion^4^, and pollution eliminating^5^. However, it is still widely recognized plenty of unknowns of SACs to be explored^6,7^, including discovering unique catalytic properties^8,9^, understanding structure–performance relations^10,11^, and realizing industrial applications^12^.

The prerequisite for the further study of SACs is to develop flexible and scalable preparation methods^13^, but it remains a great challenge.

First, developing a general route to fabricate various SACs with different metal sites and supports is very difficult^14–16^, due to their obviously different physical/chemical properties, including but not limited to the solubility, reactivity, and thermal stability of metal precursors as well as the surface coordination environments and hydrophilic/hydrophobic properties of supports^17^.

Second, the scaling-up effect seriously hampers the mass production of SACs^18,19^. Take the commonly used wet-chemistry approaches (e.g., impregnation^20^, hydrothermal treatment^21^, co-precipitation^22^, etc.) as examples. The synthesis conditions, such as concentration, temperature, stirring rate, and solution pH, have a noticeable influence on the aggregation states of the metal species^23^. Although these conditions can be well controlled on a lab scale (so the atomically dispersed structure is obtained), it is almost impossible to realize on a plant scale on account of the inevitable uneven mass transfer and heat transfer, leading to the excessive local concentrations and then the undesired aggregation of metal species^24^. One of the solutions is to introduce continuous preparation methods (increasing the production over time) instead of the current batch-type ones, but the relative reports are very rare^25,26^.

Therefore, it is a scientific and technological imperative to develop a versatile, continuous, and straightforward strategy to fabricate SACs.

In this work, we report a precursor-atomization strategy for the synthesis of SACs and their derivatives, and the whole fabrication process includes only two steps: Step 1, the dilute solution of the precursors is atomized and sprayed onto the supports; Step 2, the above samples undergo heat treatment to decompose the precursors, and the corresponding SACs are obtained. It is worth noting that according to this simple method we can build up SACs libraries for apparently various different metal‐support combinations (as many as 19 SACs and 3 derivatives) without any special precautions and the productivity can reach 1 kg day^−1^ by a homemade production line.

## Results

### Synthesis of Pd SACs with the precursor-atomization strategy

The homemade equipment was shown in Supplementary Fig. 1, which was composed of an ultrasonic atomizer (to atomize the precursor solution), three infrared lamps and a heating plate (to remove the solvent quickly), and a plastic dome shield and an iron basin (to confine the atomization in a relatively enclosed space and facilitate the recycling of the unemployed precursors, details see the “Methods” section).

**Fig. 1:**
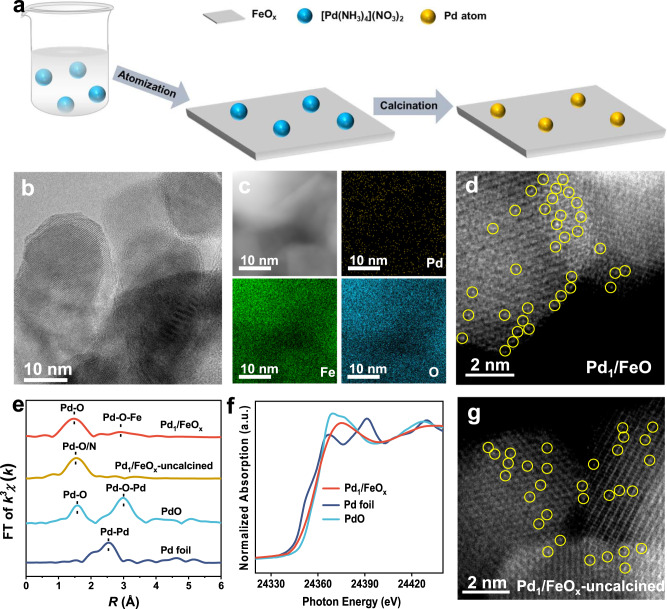
Preparation and structural characterization of Pd_1_/FeO_*x*_. **a** The scheme for the preparation of Pd_1_/FeO_*x*_ with the precursor-atomization strategy. **b** TEM image of Pd_1_/FeO_*x*_. Scale bar, 10 nm. **c** Element mapping of Pd_1_/FeO_*x*_. **d** AC HAADF-STEM image of Pd_1_/FeO_*x*_. Pd atoms were highlighted by yellow circles. Scale bar, 2 nm. **e** The *k*^3^-weighted FT spectra of Pd K-edge EXAFS for Pd_1_/FeO_*x*_, Pd_1_/FeO_*x*_-uncalcined, PdO, and Pd foil. **f** Pd K-edge XANES for Pd_1_/FeO_*x*_, PdO, and Pd foil. **g** AC HAADF-STEM image of Pd_1_/FeO_*x*_-uncalcined.

Here we take Pd_1_/FeO_*x*_ as an example to show the synthesis process (Fig. 1a). The aqueous solution of tetraamminepalladium nitrate ([Pd(NH_3_)_4_](NO_3_)_2_, 2.45 mmol L^−1^) was atomized into very small drops of ~40 μm^3^ in volume by an ultrasonic atomizer^27,28^ and sprayed at a rate of ~40 mL h^−1^ onto FeO_*x*_, which was spread out evenly on the iron basin. The infrared lamps and the heating plate were employed to remove the moisture quickly by heating the FeO_*x*_ powders to over 60 °C. After being sprayed, the sample was carefully collected and calcined at 400 °C in a tube furnace^29,30^.

To our great delight, there were no detectable Pd nanoparticles in the transmission electron microscopy (TEM) and scanning transmission electron microscopy (STEM) images of Pd_1_/FeO_x_ (Fig. 1b and Supplementary Fig. 2) and the corresponding element mapping results suggested that Pd species were uniformly distributed over Pd_1_/FeO_*x*_ (Fig. 1c). Furthermore, from the aberration-corrected high-angle annular dark-field scanning transmission electron microscopy (AC HAADF-STEM, Fig. 1d) image, isolated Pd atoms were clearly observed as bright dots. The inductively coupled plasma optical emission spectrometry (ICP–OES) and Brunauer–Emmet–Teller (BET) measurement revealed that the Pd loading and specific surface area of Pd_1_/FeO_*x*_ was 0.27 wt% and 41.6 m^2^ g^−1^, respectively (Supplementary Table 1). The X-ray diffraction (XRD, Supplementary Fig. 2) pattern of Pd_1_/FeO_*x*_ showed similar peaks as Fe_2_O_3_ (PDF#87-1165), without the Pd NPs or PdO peaks, indicating the well-dispersed Pd species in FeO_*x*_. There were two notable peaks at 1.5 and 2.9 Å (Fig. 1e), which can be ascribed to the Pd–O and Pd–O–Fe scattering paths, respectively, in agreement with the previous report^31^. It should be noted that the peak at 2.9 Å cannot be ascribed to the Pd–Pd scattering path (Pd foil, 2.5 Å) and Pd–O–Pd scattering path (PdO, 3.0 Å), which was further confirmed by the EXAFS fitting results of Pd_1_/FeO_*x*_ that these fitting results were in good agreement with the original curves (Supplementary Fig. 3 and Supplementary Table 2). Furthermore, the quantitative structural parameters results (Supplementary Table 2) of Pd_1_/FeO_*x*_ indicated that Pd atom was connected with four O atoms with a mean bond length of about 2.01 Å^31^. The wavelet transforms (WT) plot (Supplementary Fig. 4) of Pd_1_/FeO_*x*_ showed the maximum peak at ~6.2 Å^−1^, corresponding to the Pd–O scattering path by comparing Pd foil with the intensity maxima of Pd–Pd at ~10.2 Å^−1^ ^31^, which agreed with the EXAFS results in R space. These results further confirmed the dominant presence of atomically dispersed Pd species. In addition, as shown in X-ray absorption near edge structure (XANES) spectra (Fig. 1f), the absorption edge positions of Pd_1_/FeO_*x*_ were situated between those of Pd foil and PdO, indicative of the positively charged Pd species. All these characterization results corroborated the successful synthesis of Pd SACs (Pd_1_/FeO_*x*_) via the precursor-atomization strategy.

In order to understand the formation process of Pd_1_/FeO_*x*_, the samples (Pd_1_/FeO_*x*_-uncalcined), which were after being sprayed but before being calcined, were also characterized. The visible Pd atoms in AC HAADF image (Fig. 1g) and the absence of Pd–Pd bonds in EXAFS results (Fig. 1e) co-indicated that the Pd species were spatially isolated, demonstrating the molecularly dispersed states of the precursors ([Pd(NH_3_)_4_](NO_3_)_2_). Considering the complete decomposition temperature of [Pd(NH_3_)_4_](NO_3_)_2_ was about 270 °C (Supplementary Fig. 5), the samples above were subsequently treated at 400 °C, which was higher than the decomposition temperature but much lower than the migration temperature of noble metal species on metal oxide supports^32,33^, ensuring the transformation of molecularly dispersed [Pd(NH_3_)_4_](NO_3_)_2_ into atomically dispersed Pd atoms after calcination (i.e., Pd_1_/FeO_*x*_ was fabricated).

### The versatility of the precursor-atomization strategy

Surprisingly, the precursor-atomization strategy is of extraordinary flexibility for SACs fabrication through adjusting preparation parameters, including the type of precursors and the supports (details see Supplementary Table 3). These catalysts were characterized comprehensively by TEM, STEM, element mapping, X-ray absorption spectroscopy (XAS), XRD, ICP-OES, Elemental analysis (EA), and BET (see Supplementary Figs. 6–70 and Supplementary Tables 4–46) to determine the aggregation states and loadings of metal species as well as identify the texture properties of supports.

**Fig. 2:**
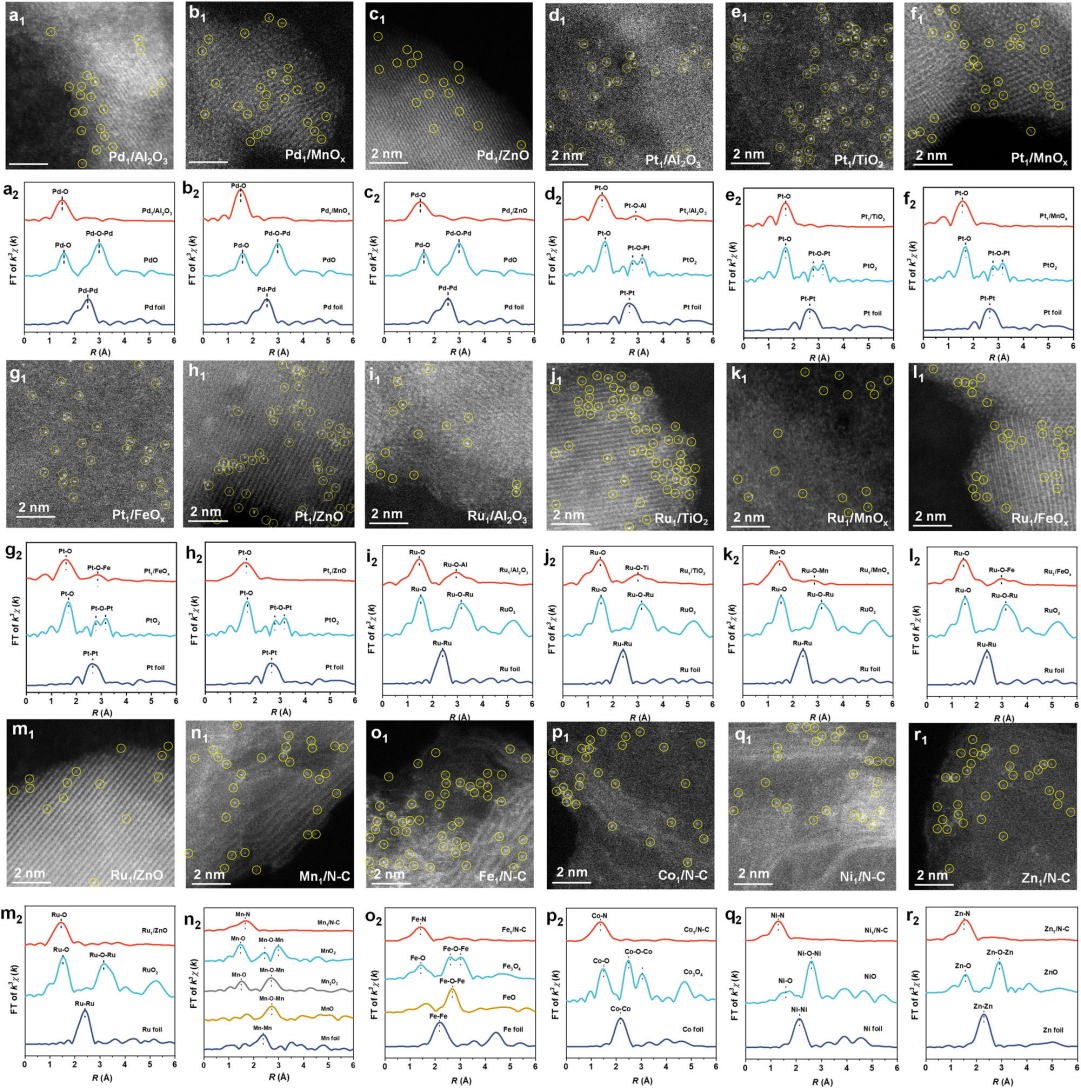
The universality of the precursor-atomization strategy. **a**_**1**_–**r**_**1**_ AC HAADF-STEM images of Pd_1_/Al_2_O_3_ (**a**_**1**_), Pd_1_/MnO_*x*_ (**b**_**1**_), Pd_1_/ZnO (**c**_**1**_), Pt_1_/Al_2_O_3_ (**d**_**1**_), Pt_1_/TiO_2_ (**e**_**1**_), Pt_1_/MnO_*x*_ (**f**_**1**_), Pt_1_/FeO_*x*_ (**g**_**1**_), Pt_1_/ZnO (**h**_**1**_), Ru_1_/Al_2_O_3_ (**i**_**1**_), Ru_1_/TiO_2_ (**j**_**1**_), Ru_1_/MnO_*x*_ (**k**_**1**_), Ru_1_/FeO_*x*_ (**l**_**1**_), Ru_1_/ZnO (**m**_**1**_), Mn_1_/N-C (**n**_**1**_), Fe_1_/N–C (**o**_**1**_), Co_1_/N–C (**p**_**1**_), Ni_1_/N–C (**q**_**1**_), Zn_1_/N-C (**r**_**1**_). Scale bar, 2 nm. **a**_**2**_–**r**_**2**_ FT-EXAFS spectra of Pd_1_/Al_2_O_3_ (**a**_**2**_), Pd_1_/MnO_*x*_ (**b**_**2**_), Pd_1_/ZnO (**c**_**2**_), Pt_1_/Al_2_O_3_ (**d**_**2**_), Pt_1_/TiO_2_ (**e**_**2**_), Pt_1_/MnO_*x*_ (**f**_**2**_), Pt_1_/FeO_*x*_ (**g**_**2**_), Pt_1_/ZnO (**h**_**2**_), Ru_1_/Al_2_O_3_ (**i**_**2**_), Ru_1_/TiO_2_ (**j**_**2**_), Ru_1_/MnO_*x*_ (**k**_**2**_), Ru_1_/FeO_*x*_ (**l**_**2**_), Ru_1_/ZnO (**m**_**2**_), Mn_1_/N–C (**n**_**2**_), Fe_1_/N–C (**o**_**2**_), Co_1_/N–C (**p**_**2**_), Ni_1_/N–C(**q**_**2**_), Zn_1_/N–C (**r**_**2**_).

First, by simply replacing [Pd(NH_3_)_4_](NO_3_)_2_ with other precursors ([Pt(NH_3_)_4_](NO_3_)_2_, Ru(NO)(NO_3_)_*x*_(OH)_*y*_, Mn(NO_3_)_2_·4H_2_O, Fe(NO_3_)_3_·9H_2_O, Co(NO_3_)_2_·6H_2_O, Ni(NO_3_)_2_·6H_2_O and Zn(NO_3_)_2_·6H_2_O) and replacing FeO_*x*_ with other supports (Al_2_O_3_, TiO_2_, MnO_*x*_, ZnO, and nitrogen-doped carbon (N–C)), as many as 18 SACs were also prepared. As shown in Fig. 2, the atomically dispersed nature of metal species was co-demonstrated by AC HAADF-STEM images (isolated metal atoms distributed throughout the supports) and EXAFS results (no corresponding metal–metal bond detected).

**Fig. 3:**
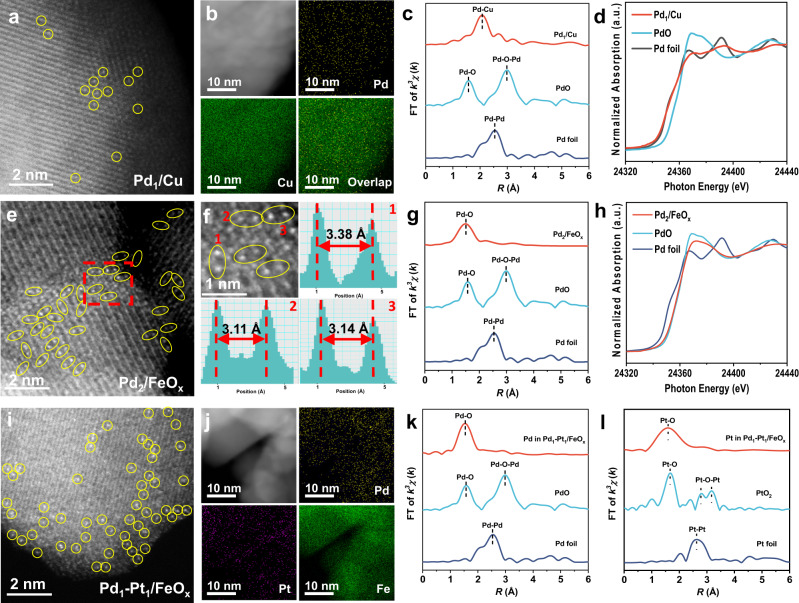
Preparation and structural characterization of SACs derivatives. **a–d** AC HAADF-STEM image, element mapping, FT-EXAFS, and XANES Pd K-edge spectra of Pd_1_/Cu. **e–h** AC HAADF-STEM image, intensity profiles obtained in areas labeled 1–3, FT-EXAFS, and XANES spectra at Pd K-edge of Pd_2_/FeO_*x*_. **i–l** AC HAADF-STEM image, element mapping, and Pd K-edge FT-EXAFS spectra of Pd_1_–Pt_1_/FeO_*x*_.

Moreover, to obtain Pd_1_/Cu single atom alloys (SAAs)^6^, the H_2_PdCl_4_ solution was sprayed on the Cu powders directly (Fig. 3a–d). As shown in Fig. 3a, there were bright dots in the catalysts of Pd_1_/Cu, which can be ascribed to the isolated Pd atoms, and the mapping result also indicated the homogenous distribution of Pd species (Fig. 3b). The EXAFS result showed no Pd–Pd bonds, and the fitting results indicated the presence of a Pd–Cu bond with the coordination numbers of ~8, similar to the previous reports of palladium–copper SAAs^18,34^ (Fig. 3c and Supplementary Fig. 62; Supplementary Table 41). The XANES and XRD results revealed that both the Pd and Cu species were primarily in the metallic states (Fig. 3d and Supplementary Fig. 60). Combining the results above, it was concluded that the Pd_1_/Cu SAAs were successfully fabricated. In addition, when we replaced [Pd(NH_3_)_4_](NO_3_)_2_ by C_6_H_10_Cl_2_Pd_2_ as the precursor, Pd DACs^7,35^ were obtained (Pd_2_/FeO_*x*_, Fig. 3e–h), featured with 3.15 Å distance between the adjacent Pd atoms in AC HAADF-STEM images (Fig. 3e, f), and exhibited the dominant peak at 1.6 Å in EXAFS results with the absence of the signal for Pd–Pd bond (2.5 Å, Fig. 3g). XANES result (Fig. 3h) suggested the positively charged Pd species on Pd_2_/FeO_*x*_, similar with those on Pd_1_/FeO_*x*_. The results above demonstrated a unique configuration of DACs that were composed of spatially adjacent atoms without strong electronic interaction. Furthermore, when the solutions of [Pd(NH_3_)_4_](NO_3_)_2_ and [Pt(NH_3_)_4_](NO_3_)_2_ were subsequently sprayed to FeO_*x*_, bi-metallic SACs^36,37^ (e.g., Pd_1_–Pt_1_/FeO_*x*_, Fig. 3i–l) were obtained, which were confirmed by the AC HAADF-STEM image (the two metal species were atomically dispersed, Fig. 3i), element mapping analysis (Pd and Pt species were homogeneously distributed, Fig. 3j), and the EXAFS results (no Pd–Pd and Pt–Pt bond, Fig. 3k, l). The high flexibility of this approach for the combination of the catalytically active sites and underlying supports constructs a huge SACs matrix, which facilitates further exploration of practical applications.

### The large-scale production of Pd_1_/FeO_*x*_ SACs

**Fig. 4:**
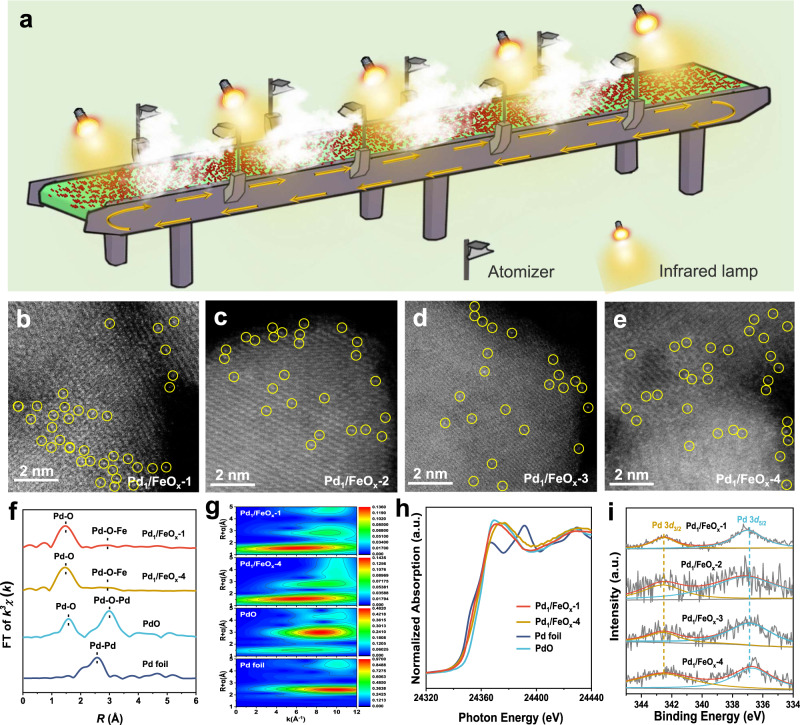
The production line based on the precursor-atomization strategy. **a** The scheme for the production line that is based on the precursor-atomization strategy. **b**–**e** AC HAADF-STEM image of Pd_1_/FeO_*x*_-1, 2, 3, 4, respectively. Pd atoms were highlighted by yellow circles. Scale bar, 2 nm. **f** The *k*^3^-weighted FT spectra of Pd K-edge EXAFS for Pd_1_/FeO_*x*_-1 and 4, PdO, and Pd foil. **g** WT of Pd K-edge EXAFS of Pd_1_/FeO_*x*_-1, 4, PdO, and Pd foil. **h** Pd K-edge XANES for Pd_1_/FeO_*x*_-1 and 4, PdO, and Pd foil. **i** XPS patterns of Pd 3*d* for Pd_1_/FeO_*x*_-1, 2, 3, 4.

Besides, the precursor-atomization strategy has great potentials for mass production of SACs. To fulfill the strategy, we designed a homemade production line (Fig. 4a, Supplementary Fig. 71, <$1000 in cost and <4 m^2^ in floor area), which was composed of 34 ultrasonic atomizers, 16 infrared lamps, and 2 pieces of 2-m-long conveyor belts (to enable Step 1 (i.e., the spray of precursor solution onto the support surface) to be carried out continuously). The continuous synthesis process was shown in Supplementary Video 1. In addition, the productivity for Pd_1_/FeO_*x*_ discussed below can reach more than 1 kg day^−1^ (Supplementary Fig. 72).

The supports, i.e., FeO_*x*_, were spread out evenly (areal density: ~120 g m^−2^) on the conveyor belt (running speed: ~1 cm min^−1^), and it took about 6 h to complete the transfer (4 m long in total). Two conveyor belts were connected end to end, and the first conveyor belt was ~10 cm higher than the second one. As a result, FeO_x_ was flipped over at the junction of the two conveyor belts and most of the surfaces of FeO_*x*_ were exposed evenly. Similarly, the solution of [Pd(NH_3_)_4_](NO_3_)_2_ was atomized and sprayed at a rate of ~40 mL h^−1^ onto FeO_*x*_ on the conveyor belts, and the infrared lamps were employed to keep the FeO_*x*_ powders dry (temperature > 60 °C). After being sprayed, the sample was collected at the end of the second conveyor belt and then calcined at 400 °C in a tube furnace. Four batches (every batch ~10 g) were collected at different times (each about 50 min apart, denoted as Pd_1_/FeO_*x*_-1, Pd_1_/FeO_*x*_-2, Pd_1_/FeO_*x*_-3, and Pd_1_/FeO_*x*_-4, respectively) were selected for the further investigation of catalyst structures and catalytic performances.

The four catalysts showed similar Pd loadings (~0.23 wt%, Supplementary Tables 47, 49–51) and specific surface areas (~44 m^2^ g^−1^, Supplementary Tables 47, 49–51) as well as a homogeneous distribution of Pd species (element mapping, Supplementary Figs. 73, 75–77). Neither Pd/PdO characteristic signals in XRD patterns nor nanoparticles in TEM/STEM images were observed (Supplementary Figs. 73, 75–77). However, visible Pd atoms were present in AC HAADF-STEM images (Fig. 4b–e). Hence, it was deduced that the Pd species on all four catalysts were atomically dispersed. This was further supported by the very similar EXAFS and WT results for Pd_1_/FeO_*x*_-1and Pd_1_/FeO_*x*_-4 (Fig. 4f, g and Supplementary Figs. 74, 78; Supplementary Table 48, 52), including the coordination numbers (~4) and bond lengths (~2.0 Å) of Pd–O shell. The XANES spectra (Fig. 4h) suggested the positively charged Pd species on both Pd_1_/FeO_*x*_-1 and Pd_1_/FeO_*x*_-4. In addition, XPS curves for Pd_1_/FeO_*x*_-2, Pd_1_/FeO_*x*_-3, and Pd_1_/FeO_*x*_-4 resembled that of Pd_1_/FeO_*x*_-1 (Fig. 4i) and thus indicated the primary presence of Pd^2+^ on all Pd_1_/FeO_*x*_ catalysts^38^. Clearly, the structures of Pd species on the four Pd_1_/FeO_*x*_ catalysts obtained at different time intervals were well retained, demonstrated by these complementary results above.

### The remarkable catalytic properties of Pd SACs

**Fig. 5:**
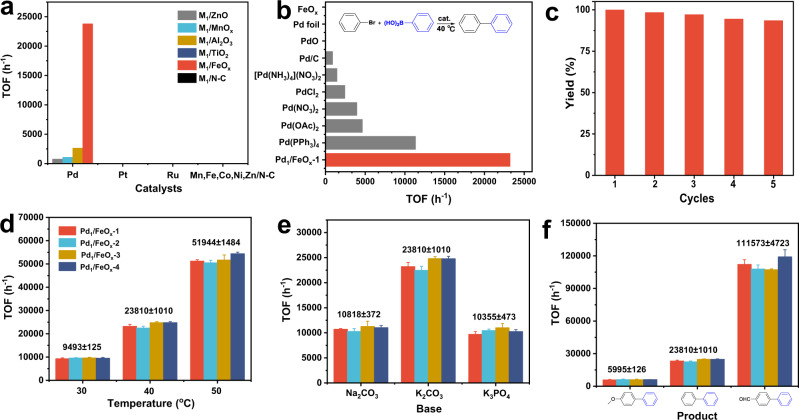
Catalytic properties of Pd_1_/FeO_*x*_ in Suzuki–Miyaura cross-coupling reaction. **a**, **b** Suzuki–Miyaura cross-coupling catalyzed by 19 SACs and different commercial Pd catalysts, respectively. Reaction conditions: 5 mg catalyst, bromobenzene (0.5 mmol), phenylboronic acid (0.75 mmol), K_2_CO_3_ (1.5 mmol), solvent (2 mL EtOH + 4 mL H_2_O), 40 °C, decane as internal standard, biphenyl yields <30%. **c** Recycling experiments over Pd_1_/FeO_*x*_-1. Reaction conditions were identical as **a**, except the amount of catalyst was 10 mg and the time was 2 h. **d** Catalytic performance of Pd_1_/FeO_*x*_-1, 2, 3, 4 at different temperatures. Reaction conditions were identical as **a**, except for the temperatures. **e** Catalytic performance of Pd_1_/FeO_*x*_-1, 2, 3, 4 with different bases. Reaction conditions were identical as **a**, except for the bases. **f** Catalytic performance of Pd_1_/FeO_x_-1, 2, 3, 4 for the formation of different products. Reaction conditions were identical as **a**, except for the substrates: 0.5 mmol (4-bromoanisole, bromobenzene or 4-bromobenzaldehyde) and 0.75 mmol phenylboronic acid, respectively. The error bars represent the standard deviation based on triplicate measurements.

**Fig. 6:**
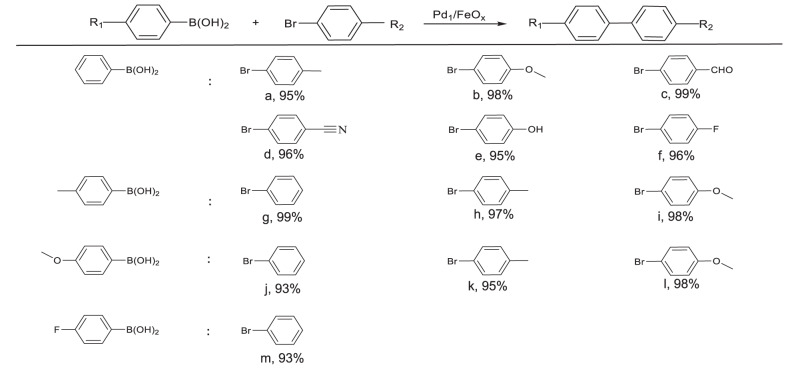
Suzuki–Miyaura cross-coupling of aryl bromide and aryl boronic acids catalyzed by Pd_1_/FeO_*x*_-1. Reaction conditions: 10 mg catalyst, aryl bromide (0.5 mmol), aryl boronic acids (0.75 mmol), K_2_CO_3_ (1.5 mmol), solvent (2 mL EtOH + 4 mL H_2_O), 40 °C, decane as internal standard, 2 h.

Suzuki–Miyaura cross-coupling is a typical homogeneous catalysis process for the formation of C–C bonds using soluble Pd/organophosphine ligands complex as catalysts, which entails tedious separation steps after reactions, thus leading to the increased cost and processing time^39,40^. To address the challenge and considering that SACs may combine the advantages of both homogeneous and heterogeneous catalysts, we tentatively tested the performance of 19 prepared SACs above with bromobenzene and phenylboronic acid as model substrates at 40 °C without inert gas protection (Fig. 5a, all the selectivities >99%). Pd-based SACs exhibited higher catalytic activity than other SACs (including Pt, Ru, and non-noble metals-based SACs), mainly because Pd was the most active metal species in the Suzuki–Miyaura cross-coupling reaction. In addition, Pd_1_/FeO_*x*_-1 showed higher activity than Pd SACs on other underlying supports. According to the previous reports^41^, Suzuki–Miyaura cross-coupling reaction proceeds generally through a three-step pathway: oxidative addition, transmetalation, and reductive elimination, so the catalysts with superior redox properties are probably suitable for this reaction. In the H_2_ temperature programmed reduction (TPR) results (Supplementary Fig. 79), Pd_1_/FeO_*x*_-1 showed the initial reduction peak at a lower temperature (~110 °C) than Pd SACs on other underlying supports, demonstrating the superior redox properties and accordingly the remarkable catalytic properties in Suzuki–Miyaura cross-coupling reaction. Moreover, the activity of the screened-out SACs of Pd_1_/FeO_*x*_-1 was much higher than that of commercial Pd catalysts, like Pd(PPh_3_)_4_, Pd(NO_3_)_2_, Pd(OAc)_2_, [Pd(NH_3_)_4_](NO_3_)_2_, Pd/C, PdO, etc., as shown in Fig. 5b, which was also comparable with that of the top-tier heterogeneous catalysts under similar reaction conditions (Supplementary Table 53). To explore the ordinary compatibility of Pd_1_/FeO_*x*_-1 for the Suzuki–Miyaura cross-coupling reaction, the substrate scope was investigated using a variety of substituted aryl bromides and aryl boronic acids as raw materials. As shown in Fig. 6, under the optimized conditions, various combinations of aryl bromides with electron-donating and electron-withdrawing groups and aryl boronic acids with electron-donating and electron-withdrawing groups were successfully converted to the desired products, and excellent yields (93–99%) were successfully obtained without the protection of the inert atmosphere. The results thus suggested that Pd_1_/FeO_*x*_-1 exhibited a good substrate tolerance toward the Suzuki–Miyaura cross-coupling reaction.

Additionally, unlike the soluble catalysts (e.g., Pd(PPh_3_)_4_), Pd_1_/FeO_x_-1 can be easily separated by filtration, and no obvious activity loss was observed after 5 cycles (Fig. 5c). Notably, the AC HAADF-STEM image showed the catalysts maintained the atomically dispersed morphology after 5 catalytic runs (Supplementary Fig. 80) and the ICP results evidenced a negligible metal leaching in solutions. These results above indicated the superiority of Pd_1_/FeO_*x*_-1, including excellent catalytic properties, structural stability and recyclability. Remarkably, the catalysts of Pd_1_/FeO_*x*_-1, Pd_1_/FeO_*x*_-2, Pd_1_/FeO_*x*_-3, and Pd_1_/FeO_*x*_-4 exhibited very similar catalytic performance (see the small standard deviations for the TOFs of the four catalysts) under different reaction conditions, including temperatures (9493 ± 125, 23810 ± 1010, and 51,944 ± 1484 h^−1^ at 30, 40, and 50 °C, respectively, Fig. 5d), bases (10,818 ± 372, 23,810 ± 1010, and 10,355 ± 473 h^−1^ for Na_2_CO_3_, K_2_CO_3_, and K_3_PO_4_, respectively, Fig. 5e), or with different substrates (5995 ± 126 and 111,573 ± 4723 h^−1^ for aryl bromides bearing electron-donating and electron-withdrawing groups, respectively, Fig. 5f). Combined with the above results of structure characterizations, it demonstrated the identical catalytically active sites over the four Pd_1_/FeO_*x*_ catalysts and thus the excellent reproducibility of our synthesis approach, which was significant for the subsequent industrial applications.

## Discussion

In summary, we have developed a versatile precursor-atomization strategy with great potential to precisely manipulate and arrange the atoms to obtain a library for SACs (including 19 SACs as well as SAAs, DACs, and bi-metallic SACs), basically covering the widely concerned catalyst structures of SACs at present. A homemade production line is established for the large-scale synthesis of SACs and the productivity can be increased to over 1 kg day^−1^. The prepared Pd_1_/FeO_*x*_ shows identical catalyst structures and catalytic properties in Suzuki–Miyaura cross-coupling irrespective of different batches in production, demonstrating the remarkable reproductivity and thus the great potential for the large-scale synthesis of SACs.

## Methods

### Catalyst preparation

Here, we took Pd_1_/FeO_*x*_ as an example to illustrate the preparation process. The homemade equipment was shown in Supplementary Fig. 1, which was composed of an ultrasonic atomizer (Shenzhen Ikone Technology Co., Ltd), three infrared lamps (Royal Dutch Philips Electronics Ltd.), a heating plate, a plastic dome shield (ø 30 cm), an iron plate (ø 30 cm), and some cotton (for filling gap). First, take 12 g FeO_*x*_ powders and spread them evenly on the iron plate. Then the tetraamminepalladium(II) nitrate aqueous solution (2.45 mmol/L) was sprayed onto the surface of FeO_*x*_ through an atomizer with a rate of ~40 mL/h. To make the precursors deposit homogeneously onto the different surfaces of FeO_*x*_, the FeO_*x*_ powders were re-spread every hour. After a cumulative consumption of 0.16 L of solution, samples were collected and then treated at 400 °C (heating rate: 5 °C/min) for 2 h under 100 mL/min airflow in a tube furnace (Hefei Kejing Material Technology Co., Ltd), and Pd_1_/FeO_*x*_ (Pd: 0.27 wt%) was obtained. Afterward, the dome shield, iron dish, and cotton were thoroughly washed to recycle the unused precursors. Based on the ICP results, ~80% noble metal precursors ended up depositing on the supports, and ~7% on the plastic dome shield, the iron dish, and cotton, respectively, and the waste of precursors was <13%.

To verify the versatility of the precursor-atomization strategy, a series of catalysts, including Pd_1_/Al_2_O_3_, Pd_1_/MnO_*x*_, Pd_1_/ZnO, Pt_1_/Al_2_O_3_, Pt_1_/TiO_2_, Pt_1_/MnO_*x*_, Pt_1_/FeO_*x*_, Pt_1_/ZnO, Ru_1_/Al_2_O_3_, Ru_1_/TiO_2_, Ru_1_/MnO_*x*_, Ru_1_/FeO_*x*_, Ru_1_/ZnO, Mn_1_/N–C, Fe_1_/N–C, Co_1_/N–C, Ni_1_/N–C, Zn_1_/N–C, Pd_1_/Cu, Pd_2_/FeO_*x*_, and Pd_1_–Pt_1_/FeO_*x*_, were prepared with similar synthesis procedures and the detailed parameters were given in Supplementary Table 3. For convenience, simplified homemade equipment was used here, which was only composed of an ultrasonic atomizer, an infrared lamp, a turntable (Shenzhen Xuriqiqi Technology Co., Ltd), and a glass dish. N–C was prepared as follows: the mixture of carbon black and 2-methylimidazole (weight ratio is 1:1) was milled for 10 h at 400 r/min in a planetary ball mill (CHISHUN, QM3SP2).

The process of prepared Pd_1_/FeO_*x*_ on a large scale was as follows. The homemade production line was shown in Supplementary Fig. 71, which was composed of 2 pieces of 2-m-long conveyor belts (0.5-m width, Dongguan Hengyuntong Industrial Equipment Co., Ltd.), 34 ultrasonic atomizers, and 16 infrared lamps. The first conveyor belt was ~10 cm higher than the second one, and the atomizers and infrared lamps were evenly distributed on both sides of the conveyor belts. FeO_*x*_ evenly dispersed (~120 g/m^2^) on the surface of the conveyor belt was conveyed at a constant speed (~1 cm/min). Tetraamminepalladium(II) nitrate aqueous solution (2.45 mmol/L) was atomized and sprayed onto the FeO_*x*_ through commercial ultrasonic atomizers (flow rate: ~40 mL/h). Afterward, the samples were collected at the end of the second conveyor belt and calcined in the tube furnace under airflow at 400 °C for 2 h.

### Characterization

EA was performed on a Vario EL cube instrument, and ICP-OES was carried out on a PerkinElmer OPTIMA 8000DV. A Micromeritic ASAP2020M analyzer was employed to conduct BET surface area measurements at liquid nitrogen temperature. The samples underwent vacuum treatment for about 6 h at 200 °C prior to the measurement. XRD measurements were carried out on a Bruker D8 Advanced diffractometer and the 2*θ* range was 10–80°. XPS measurements were performed on an ESCALab250 XPS system equipped with an Al Kα source and a charge neutralizer, and all the binding energies were referenced to the contaminated C 1*s* (284.8 eV). Both TEM and STEM images were obtained on an FEI Tecnai G2 F30, which was operated at 300 kV. AC HAADF-STEM images and element mapping were obtained on a JEM-ARM200F transmission electron microscopy incorporated with double spherical aberration correctors, operating at 200 kV. X-ray absorption spectroscopy (XAS) measurements were conducted on BL14W beamline at the Shanghai Synchrotron Radiation Facility (SSRF), 20-BM-B beamline at the Advanced Photon Source at Argonne National Laboratory (APS), and Singapore Synchrotron Light Source (SSLS). The samples were measured in fluorescence mode using Lytle detector (SSRF), 32-element Ge solid state detector (APS), or silicon drift detector (SSLS) and the corresponding metal foils and metal oxides were used as reference samples and measured in the transmission mode. Thermogravimetric analysis (TGA) results were obtained using a TG209F1 libra instrument. ^1^H and ^13^C nuclear magnetic resonance (NMR) spectra were obtained on Bruker Avance III 500 HD. H_2_ TPR was performed on a DAS-7200.

### Catalytic performance test

The typical performance evaluation was carried out as follows: bromobenzene (0.5 mmol), phenylboronic acid (0.75 mmol), K_2_CO_3_ (1.5 mmol), 5 mg Pd_1_/FeO_*x*_ (Pd:substrate = 1:4000 (mol:mol)) and solvent (2 mL EtOH + 4 mL H_2_O) were added to a 25 mL Schlenk tube, using decane as an internal standard. The mixture in the tube was stirred at 40 °C in the air atmosphere. After the reaction, the cooled mixture was first extracted with ethyl acetate (3 mL × 3), and the organic phase was collected and dried over anhydrous sodium sulfate. Product yields of the reaction were measured using GC (Shimadzu 2010 GC Plus), and the structure of the products was confirmed by GC–MS (Shimadzu GCMS-QP2010 Ultra) analysis. The corresponding NMR data are provided in Supplementary information (Supplementary Figs. 81–100). The catalyst was filtered and washed with ethanol and water several times, dried at 80 °C for 10 h and calcined in the air at 400 °C for 2 h, and then reused for the next cycle experiment.

The TOF is calculated at <30% conversion level and the Pd dispersion is assumed to be 100% (Eq. (1)):

1 $${{{{{\rm{TOF}}}}}}={n}_{0}\,\ast \,C\,\ast \,106.42/t\,\ast \,{m}_{{{{{{\rm{cat}}}}}}}\,\ast \,{{{{{\rm{\omega }}}}}}$$

where *n*_0_ is the initial number of moles of the substrate, *C* is the conversion of the substrate at reaction time *t*, *m*_cat_ is the weight of the catalyst, and *ω* is Pd loading.

The yield is based on the bromobenzene or its derivatives, using decane as the internal standard, and the calculation equation is shown as Eq. (2).

2 $${{{{{\rm{Yield}}}}}}=n/{n}_{0}$$

where *n* is the number of moles of the product, and *n*_0_ is the initial number of moles of the substrate.

The calculation equation of selectivity is shown as Eq. (3)

3 $${{{{{\rm{Selectivity}}}}}}=n/({n}_{0}\ast C)$$

where *n* is the number of moles of product, *n*_0_ is the initial number of moles of the substrate, and *C* is the conversion of the substrate.

## Supplementary information

Supplementary Information

Peer Review File

Description of Additional Supplementary Files

Supplementary Video 1

## Data Availability

The data underlying Figs. 1–5, and Supplementary Figs. 2, 3, 5–60, 62–63, 65–70, 73–79 are provided as a Source Data file. The other data that support the findings of this study are available from the corresponding author upon request. Source data are provided with this paper.

## Source data

Source Data
