# Supplementary material for: Building up libraries and production line for single atom catalysts with precursor-atomization strategy
Source: Nat Commun. 2022 Sep 29;13:5721. doi: 10.1038/s41467-022-33442-2 (PMC9522824; doi:10.1038/s41467-022-33442-2)
Supplement: Supplementary file 1 — Supplementary Information [file 41467_2022_33442_MOESM1_ESM.pdf]

Supplementary Information

Building up libraries and production line for single atom catalysts with precursor-atomization strategy

Xiaohui He[1], Hao Zhang[1], Xingcong Zhang[1], Ying Zhang[1], Qian He[1], Hongyu Chen[1], Yujie Cheng[1], Mi Peng[2], Xuetao Qin[2], Hongbing Ji[1,3]*, and Ding Ma[2]*

[1]Fine Chemical Industry Research Institute, School of Chemistry, Sun Yat-sen University, Guangzhou 510275, China.

[2]Beijing National Laboratory for Molecular Sciences, College of Chemistry and Molecular Engineering and College of Engineering, and BIC-ESAT, Peking University, Beijing 100871, China.

[3]Huizhou Research Institute, Sun Yat-sen University, Huizhou 516081, China.

[*]Correspondence and requests for materials should be addressed to H.J. (email: jihb@mail.sysu.edu.cn) or to D.M. (email: dma@pku.edu.cn).

Supplementary Methods

Materials. All the solvents and chemicals were available from suppliers and used as received unless specially stated.

Tetraamminepalladium(II) nitrate ($[Pd(NH_3)_4](NO_3)_2$, Macklin, 98%), allylpalladium(II) chloride ($C_6H_{10}Cl_2Pd_2$, Bide Pharmatech Ltd., 97%), Palladium(II) Chloride ($PdCl_2$, Adamas, 99%), tetraammineplatinum(II) nitrate ($(Pt(NH_3)_4](NO_3)_2$, Alfa Aesar, 99.9%), ruthenium(III) nitrosyl nitrate solution ($Ru(NO)(NO_3)_x(OH)_y$, x+y=3, Macklin, Ru 1.5% w/v), manganese nitrate tetrahydrate ($Mn(NO_3)_2·4H_2O$, Saan Chemical Technology (Shanghai) Co., Ltd, 99%), ferric nitrate monohydrate ($Fe(NO_3)_3·9H_2O$, Sinopharm chemical reagent co. Ltd, 99%), cobalt nitrate hexahydrate ($Co(NO_3)_2·6H_2O$, Aladdin, 99%), nickel nitrate hexahydrate ($Ni(NO_3)_2·6H_2O$, Tianjin Damao Chemical Reagent Factory, 99%), zinc nitrate hexahydrate ($Zn(NO_3)_2·6H_2O$, Aladdin, 99%). Tetrakis(triphenylphosphine)palladium ($Pd[P(C_6H_5)_3]_4$, Saan Chemical Technology (Shanghai) Co., Ltd, 99%), Palladium(II) nitrate dihydrate ($Pd(NO_3)_2 ·2H_2O$, Adamas, 99%), Palladium(II) acetate ($Pd(OAc)_2$, Adamas, 99%), Palladium 5% on Carbon (Pd/C, Macklin, 99%), Palladium foil (Pd foil, Alfa Aesar, 99.9%)

Nano copper powder (Cu, Macklin, 99.9%), ferric oxide (Fe_2O_3, Shanghai Yaotian new material technology co., Ltd, 99%), manganese oxide (Shanghai Yaotian new material technology co., Ltd, 99%), zinc oxide (ZnO, Shanghai Yaotian new material technology co., Ltd, 99%), nano alumina (Al_2O_3, Macklin, 99%), titanium oxide (TiO_2, Aladdin, 99%), 2-methylimidazole (Aladdin, 98%), carbon black(Alfa Aesar, 99.9%).

Bromobenzene (Adamas, 99%), 4-bromoanisole (Adamas, 99%), 4-bromobenzaldehyde (Adamas, 99%), phenylboronic acid (Adamas, 98%), biphenyl (Adamas, 99%), 4-methoxybiphenyl (Adamas, 98%), 4-phenylbenzaldehyde (Adamas, 99%), ethanol (Greagent, 99.5%), ethyl acetate (Greagent, 99.5%), decane (TCI, 99%), potassium carbonate (K_2CO_3, Greagent, 99%), tripotassium orthophosphate (K_3PO_4, Adamas, 99%), Sodium carbonate (Na_2CO_3, Macklin, 99.5%).

4-Phenylphenol (Supplementary Figs. 82-83): ^{1}H NMR (500 MHz, $CDCl_3$) δ 7.55 (d, J = 7.6 Hz, 2H), 7.49 (d, J = 8.6 Hz, 2H), 7.42 (t, J = 7.8 Hz, 2H), 7.35 – 7.28 (m, 1H), 6.91 (d, J = 8.6 Hz, 2H), 4.99 (s, 1H); ^{13}C NMR (101 MHz, CD_3CN) δ 157.62, 141.69, 133.57, 129.79, 129.07, 127.57, 127.33, 116.62.

4,4'-Dimethoxybiphenyl (Supplementary Figs. 84-85): ^{1}H NMR (500 MHz, $CDCl_3$) δ 7.50 (d, J = 8.8 Hz, 4H), 6.98 (d, J = 8.7 Hz, 4H), 3.86 (s, 6H); ^{13}C NMR (126 MHz, $CDCl_3$) δ 158.8, 133.6, 127.8, 114.3, 55.5.

4-Phenyltoluene (Supplementary Figs. 86-87): ^{1}H NMR (500 MHz, $CDCl_3$) δ 7.60 (d, J = 7.0 Hz, 2H), 7.52 (d, J = 8.1 Hz, 2H), 7.45 (t, J = 7.7 Hz, 2H), 7.35 (td, J = 7.2, 1.3 Hz, 1H), 7.28 (d, J = 7.9 Hz, 2H), 2.42 (s, 3H); ^{13}C NMR (101 MHz, CD_3CN) δ 141.80, 138.95, 138.27, 130.49, 129.81, 128.07, 127.74, 127.67, 21.08.

4-Phenylbenzaldehyde (Supplementary Figs. 88-89): ^{1}H NMR (400 MHz, CD_3CN) δ 10.04 (s, 1H), 7.97 (d, J = 8.5 Hz, 2H), 7.84 (d, J = 8.3 Hz, 2H), 7.72 (dd, J = 8.3, 1.3 Hz, 2H), 7.54 – 7.48 (m, 2H), 7.47 – 7.41 (m, 1H); ^{13}C NMR (101 MHz, CD_3CN) δ 193.18, 147.60, 140.47, 136.51, 131.01, 130.05, 129.50, 128.57, 128.26.

4-Methoxybiphenyl (Supplementary Figs. 90-91): ^{1}H NMR (500 MHz, $CDCl_3$) δ 7.58 – 7.51 (m, 4H), 7.42 (t, *J* = 7.7 Hz, 2H), 7.34 – 7.29 (m, 1H), 6.99 (d, *J* = 8.7 Hz, 2H), 3.86 (s, 3H); ^{13}C NMR (126 MHz, $CDCl_3$) δ 159.3, 141.0, 133.9, 128.9, 128.3, 126.9, 126.8, 114.3, 55.5.

4,4'-Dimethylbiphenyl (Supplementary Figs. 92-93): ^{1}H NMR (500 MHz, $CDCl_3$) δ 7.47 (d, *J* = 8.2 Hz, 4H), 7.22 (d, *J* = 7.9 Hz, 4H), 2.38 (s, 6H); ^{13}C NMR (126 MHz, $CDCl_3$) δ 138.4, 136.8, 129.6, 126.9, 21.2.

4-Phenylbenzonitrile (Supplementary Figs. 94-95): ^{1}H NMR (500 MHz, $CDCl_3$) δ 7.73 (d, *J* = 8.5 Hz, 2H), 7.68 (d, *J* = 8.5 Hz, 2H), 7.59 (dd, *J* = 8.3, 1.3 Hz, 2H), 7.49 (t, *J* = 7.4 Hz, 2H), 7.45 – 7.40 (m, 1H); ^{13}C NMR (126 MHz, $CDCl_3$) δ 145.8, 139.3, 132.7, 129.2, 128.8, 127.8, 127.3, 119.0, 111.0.

4-Fluorobiphenyl (Supplementary Figs. 96-97): ^{1}H NMR (500 MHz, $CDCl_3$) δ 7.58 – 7.53 (m, 4H), 7.45 (t, *J* = 7.7 Hz, 2H), 7.38 – 7.33 (m, 1H), 7.17 – 7.10 (m, 2H); ^{13}C NMR (126 MHz, $CDCl_3$) δ 163.58, 161.62, 140.40, 137.49, 137.46, 128.96, 128.85, 128.79, 127.39, 127.16, 115.83, 115.66.

4-methoxy-4'-methylphenyl (Supplementary Figs. 98-99): ^{1}H NMR (500 MHz, $CDCl_3$) δ 7.52 (d, *J* = 8.7 Hz, 1H), 7.46 (d, *J* = 8.3 Hz, 1H), 7.24 (d, *J* = 7.8 Hz, 1H), 6.98 (d, *J* = 8.8 Hz, 1H), 3.85 (s, 3H), 2.39 (s, 3H); ^{13}C NMR (126 MHz, $CDCl_3$) δ 159.06, 138.10, 136.49, 133.89, 129.57, 128.09, 126.72, 114.29, 55.48, 21.20.

Diphenyl (Supplementary Fig. 100-101): ^{1}H NMR (500 MHz, $CDCl_3$) δ 7.64 (dd, *J* = 8.3, 1.3 Hz, 4H), 7.48 (t, *J* = 7.7 Hz, 4H), 7.41 – 7.34 (m, 2H); ^{13}C NMR (126 MHz, $CDCl_3$) δ 141.37, 128.88, 127.38, 127.30.

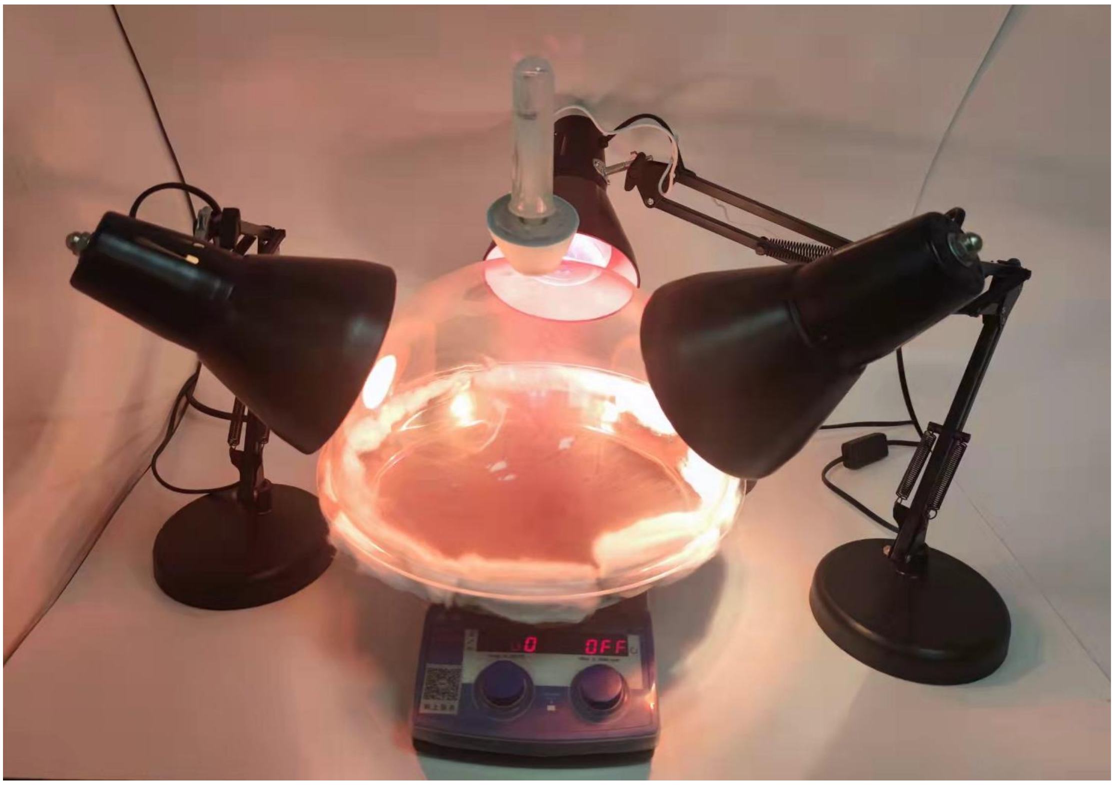

Supplementary Fig. 1 | Preparation of Pd_1/FeO_x. The picture of the homemade equipment for the synthesis of Pd_1/FeO_x.

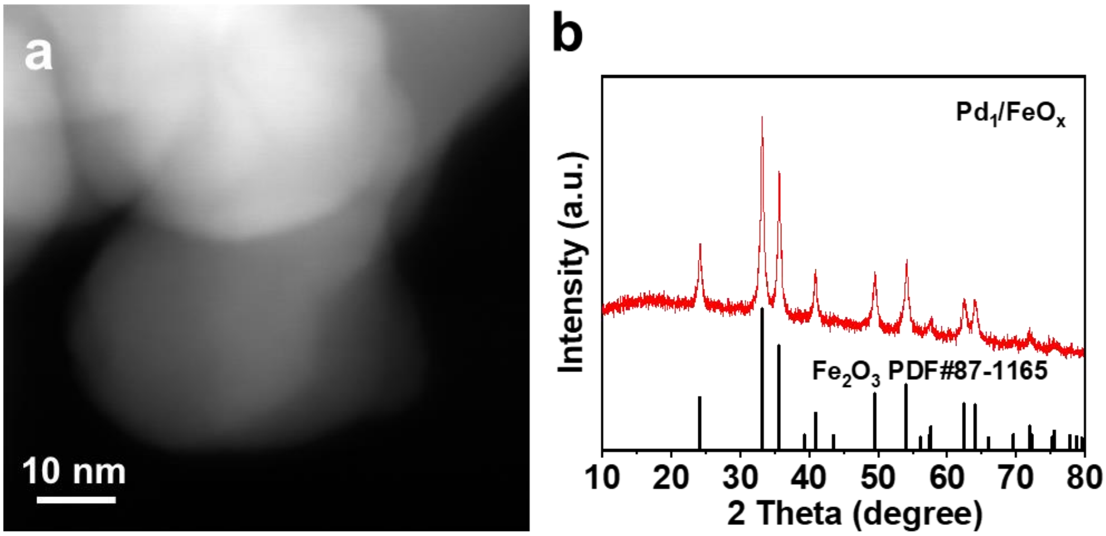

Supplementary Fig. 2 | Structural characterization results of Pd_1/FeO_x. a STEM image and **b** XRD pattern of Pd_1/FeO_x. Scale bar, 10 nm.

Supplementary Table 1. Pd content and BET surface area for Pd_1/FeO_x

Sample	Pd (wt%) [a]	BET surface area (m^2 g^{-1}) [b]
Pd_1/FeO_x	0.27	41.6

[a] ICP-OES. [b] Sorption isotherm of N_2 at 77 K.

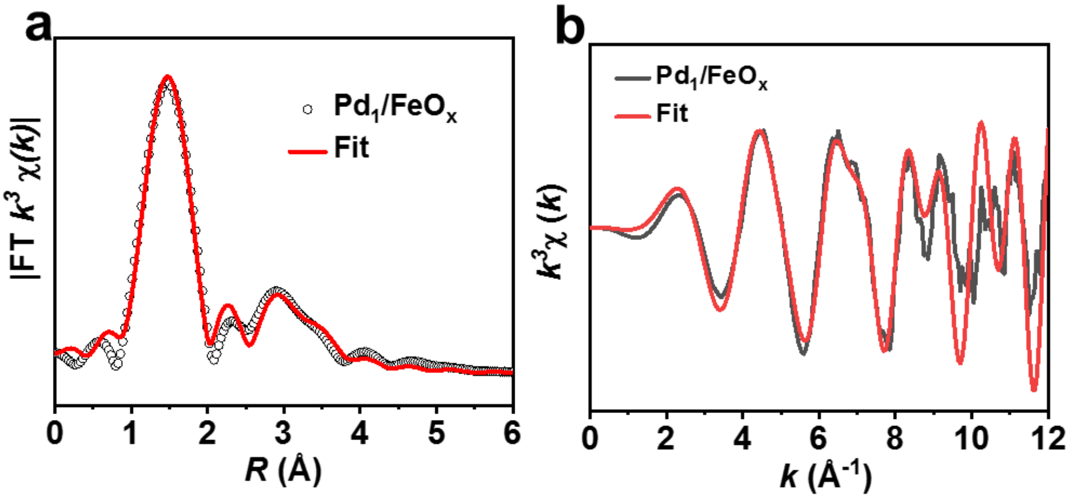

Supplementary Fig. 3 | EXAFS fitting for the Pd_1/FeO_x. **a** Fourier transform (FT) k^3-weighted $\chi(k)$-function of the EXAFS spectra for Pd K-edge and corresponding R-space fitting curves for the Pd_1/FeO_x catalyst. **b** EXAFS k space fitting curve and the experimental one of Pd_1/FeO_x.

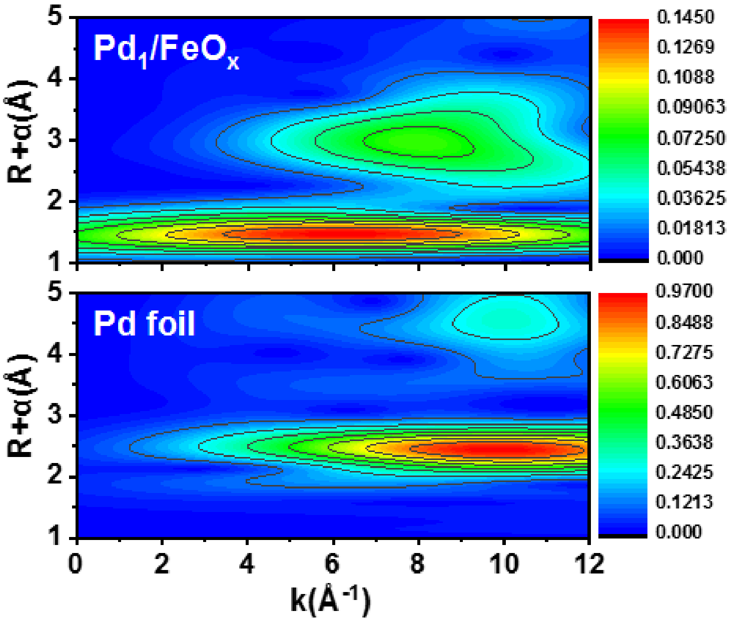

Supplementary Fig. 4 | WT results. WT of Pd K edge EXAFS of Pd_1/FeO_x and Pd foil.

Supplementary Table 2. Structural parameters of EXAFS fitting for the Pd_1/FeO_x

Sample	Scattering pair	CN [a]	R (Å) [b]	σ^2 ($\times 10^{-3}$ Å^2) [c]	ΔE_0 (eV) [d]	R factor [e]
Pd_1/FeO_x	Pd-O	4.2±0.6	2.01±0.01	4.3±1.7	1.0±1.7	0.01
	Pd-O-Fe	0.8±0.7	3.61±0.02	4.7±7.9		

[a] CN is the coordination number; [b] R is interatomic distance (the bond length between central atoms and surrounding coordination atoms); [c] σ^2 is Debye-Waller factor (a measure of thermal and static disorder in absorber-scatterer distances); [d] ΔE_0 is edge-energy shift (the difference between the zero kinetic energy value of the sample and that of the theoretical model). [e] R factor is used to value the goodness of the fitting. Data range: $2.8 \leq k \leq 8.7$ Å^{-1}, $1.0 \leq R \leq 4.3$ Å.

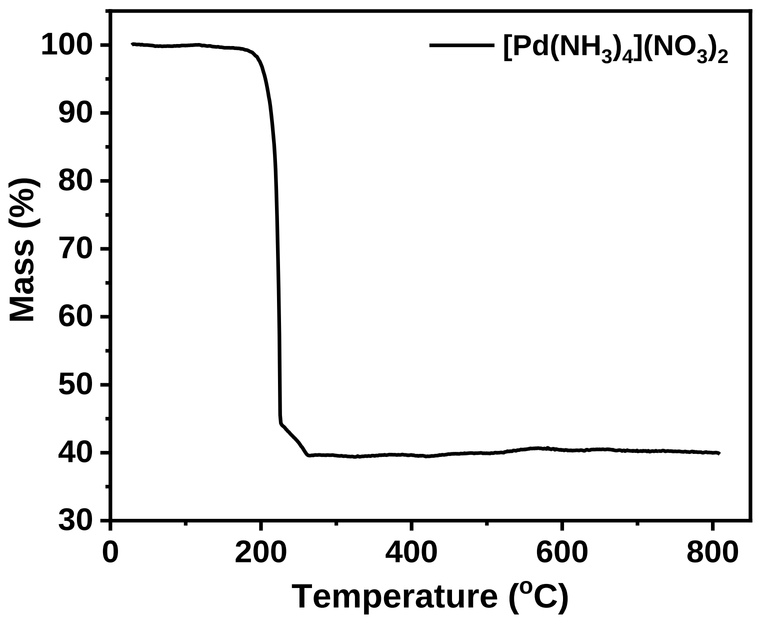

Supplementary Fig. 5 | Thermogravimetric analysis results. Thermogravimetric analysis of tetraamminepalladium(II) nitrate (10 °C/min, air).

Supplementary Table 3. The preparation parameters of catalysts in this work

Entry	Catalyst	Preparation scale (g)	Metal precursor	Solvent	Solution concentration (mmol/L)	Total volume of consumed solution (L)	Heat treatment conditions		
							Atmosphere	Temperature (°C)	Time (h)
1	Pd_1/Fe_2O_3	12	$[Pd(NH_3)_4](NO_3)_2$	H_2O	2.45	0.16	Air	400	2
2	Pd_1/Al_2O_3	1	$[Pd(NH_3)_4](NO_3)_2$	H_2O	1.45	0.60	Air	400	2
3	Pd_1/MnO_x	1	$[Pd(NH_3)_4](NO_3)_2$	H_2O	0.45	1.60	Air	400	2
4	Pd_1/ZnO	2	$[Pd(NH_3)_4](NO_3)_2$	H_2O	1.45	0.85	Air	400	2
5	Pd_1/Cu	2	H_2PdCl_4	H_2O	1.65	1.25	10% H_2/N_2	300	1
6[a]	Pt_1/Al_2O_3	1	$[Pt(NH_3)_4](NO_3)_2$	H_2O	0.65	0.24	Air	400	2
7	Pt_1/TiO_2	1	$[Pt(NH_3)_4](NO_3)_2$	H_2O	0.65	0.75	Air	400	2
8	Pt_1/MnO_x	1	$[Pt(NH_3)_4](NO_3)_2$	H_2O	0.65	1.30	Air	400	2
9	Pt_1/FeO_x	1	$[Pt(NH_3)_4](NO_3)_2$	H_2O	2.00	0.32	Air	400	2
10	Pt_1/ZnO	1	$[Pt(NH_3)_4](NO_3)_2$	H_2O	0.65	0.60	Air	400	2
11	Ru_1/Al_2O_3	1	$Ru(NO)(NO_3)_x(OH)_y$	H_2O	0.60	1.70	Air	400	2
12	Ru_1/TiO_2	1	$Ru(NO)(NO_3)_x(OH)_y$	H_2O	0.60	1.70	Air	400	2
13	Ru_1/MnO_x	1	$Ru(NO)(NO_3)_x(OH)_y$	H_2O	0.60	2.00	Air	400	2
14	Ru_1/FeO_x	1	$Ru(NO)(NO_3)_x(OH)_y$	H_2O	0.60	2.60	Air	400	2
15	Ru_1/ZnO	1	$Ru(NO)(NO_3)_x(OH)_y$	H_2O	0.60	2.50	Air	400	2
16	Co_1/N-C	1	$Co(NO_3)_2 \cdot 6H_2O$	methanol	2.50	0.80	N_2	600	2
17	Mn_1/N-C	2	$Mn(NO_3)_2 \cdot 4H_2O$	methanol	2.50	1.40	N_2	600	2
18	Fe_1/N-C	2	$Fe(NO_3)_3 \cdot 9H_2O$	methanol	5.00	1.30	N_2	600	2
19	Ni_1/N-C	2	$Ni(NO_3)_2 \cdot 6H_2O$	methanol	5.00	1.20	N_2	600	2

20	Zn_1/N-C	2	$Zn(NO_3)_2{\cdot}6H_2O$	methanol	2.50	1.60	N_2	600	2
21	Pd_2/FeO_x,	2	$C_6H_{10}Cl_2Pd_2$	methanol	1.35	0.60	Air	400	2
22	Pd_1-Pt_1/FeO_x	1	$[Pd(NH_3)_4](NO_3)_2$	H_2O	2.50	0.40	Air	400	2
23			$[Pt(NH_3)_4](NO_3)_2$		2.00	0.20			

[a] H_2PdCl_4 aqueous solution was prepared as following: $PdCl_2$ (44.5 mg) was first completely dissolved in HCl a.q. (20 mM, 25 mL) at 100 ℃, and then water was added to obtain the solution with desired concentration.

Pd_1/Al_2O_3

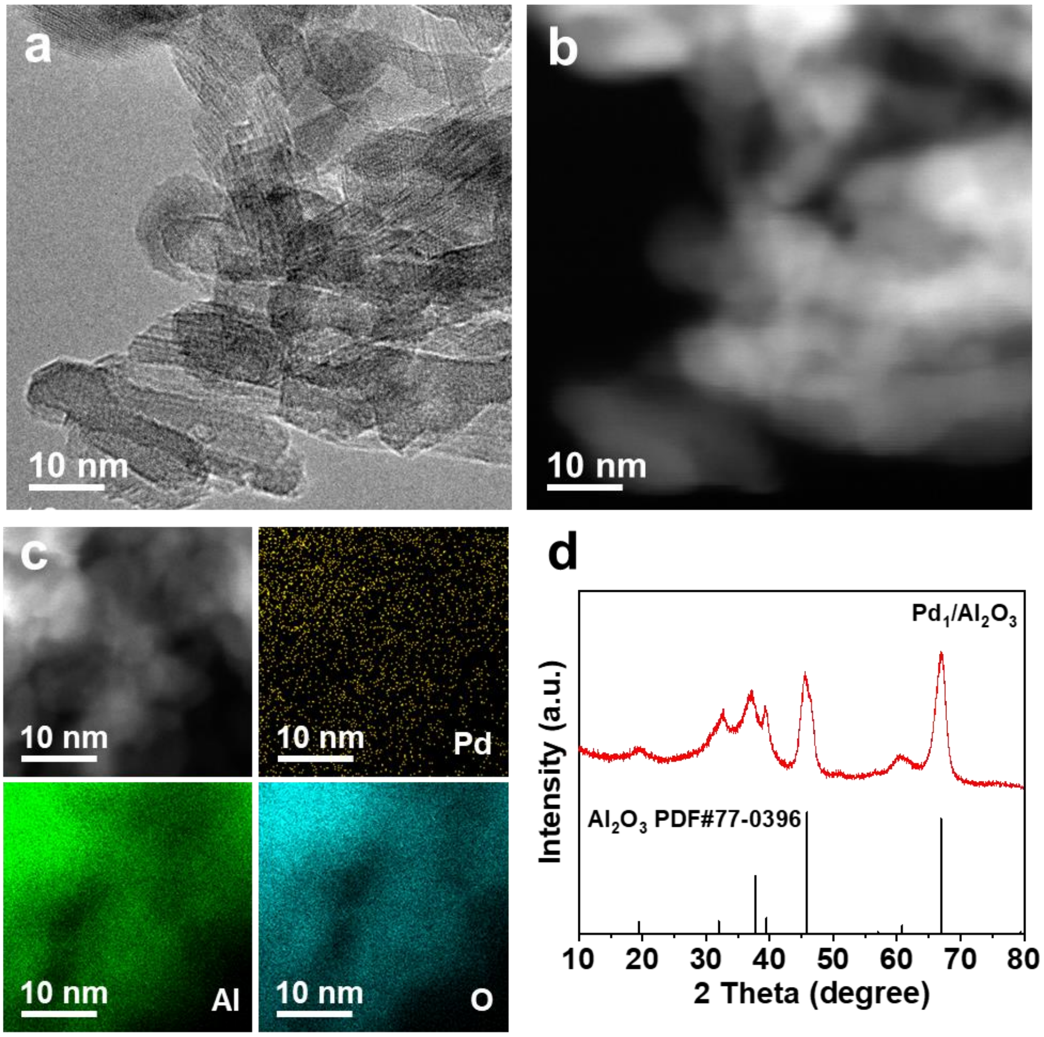

Supplementary Fig. 6 | Structural characterization results of Pd_1/Al_2O_3. a TEM image, **b** STEM image, **c** elemental mapping, and **d** XRD pattern of Pd_1/Al_2O_3. Scale bar, 10 nm.

Supplementary Table 4. Pd content and BET surface area for Pd_1/Al_2O_3

Sample	Pd (wt%) [a]	BET surface area ($m^2\ g^{-1}$) [b]
Pd_1/Al_2O_3	0.31	118.0

[a] ICP-OES. [b] Sorption isotherm of N_2 at 77 K.

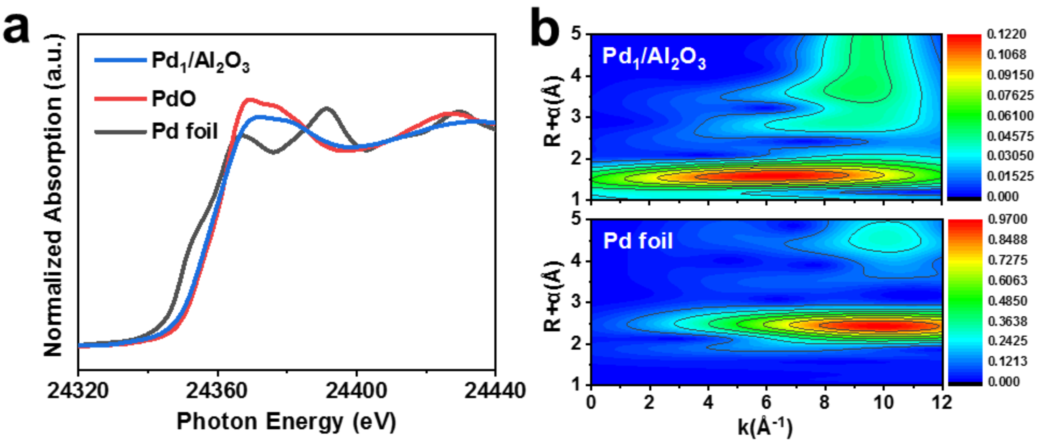

Supplementary Fig. 7 | Structural characterization results of Pd_1/Al_2O_3. a XANES Pd K-edge for Pd_1/Al_2O_3, PdO, and Pd foil. **b** WT of Pd K edge EXAFS of Pd_1/Al_2O_3 and Pd foil.

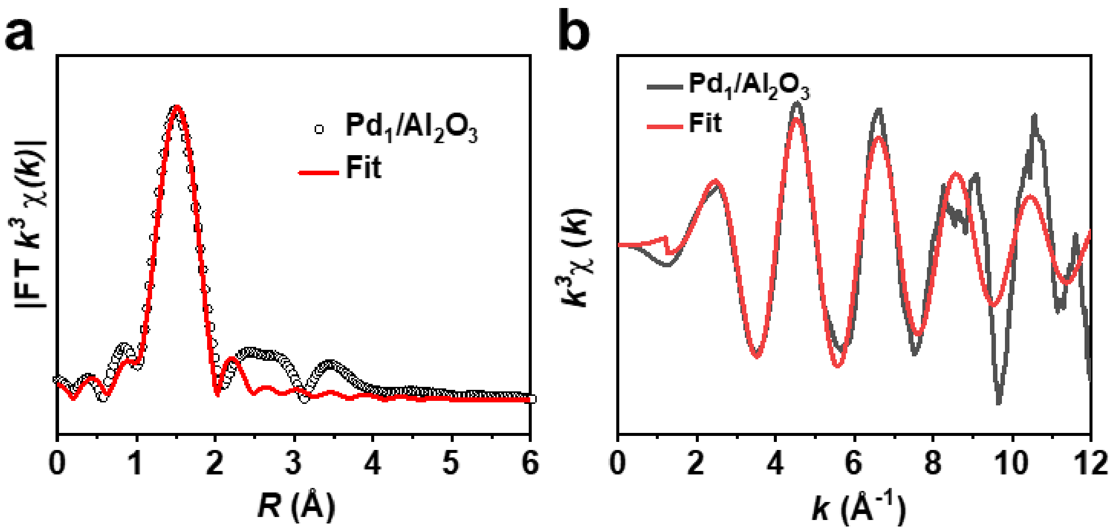

Supplementary Fig. 8 | EXAFS fitting for the Pd_1/Al_2O_3. a FT k^3-weighted $\chi(k)$-function of the EXAFS spectra for Pd K-edge and corresponding R-space fitting curves for the Pd_1/Al_2O_3 catalyst. **b** EXAFS *k* space fitting curve and the experimental one of Pd_1/Al_2O_3.

Supplementary Table 5. Structural parameters of EXAFS fitting for the Pd_1/Al_2O_3

Sample	Scattering pair	CN [a]	R (Å) [b]	σ^2 (× 10^{-3} Å^2) [c]	ΔE_0 (eV) [d]	R factor [e]
Pd_1/Al_2O_3	Pd-O	3.7±0.5	2.04±0.01	6.4±2.1	5.6±1.3	0.01

[a] CN is the coordination number; [b] R is interatomic distance (the bond length between central atoms and surrounding coordination atoms); [c] σ^2 is Debye-Waller factor (a measure of thermal and static disorder in absorber-scatterer distances); [d] ΔE_0 is edge-energy shift (the difference between the zero kinetic energy value of the sample and that of the theoretical model). [e] R factor is used to value the goodness of the fitting. Data range: $2.0 \le k \le 9.3$ Å^{-1}, $1.0 \le R \le 2.5$ Å.

Pd_1/MnO_x

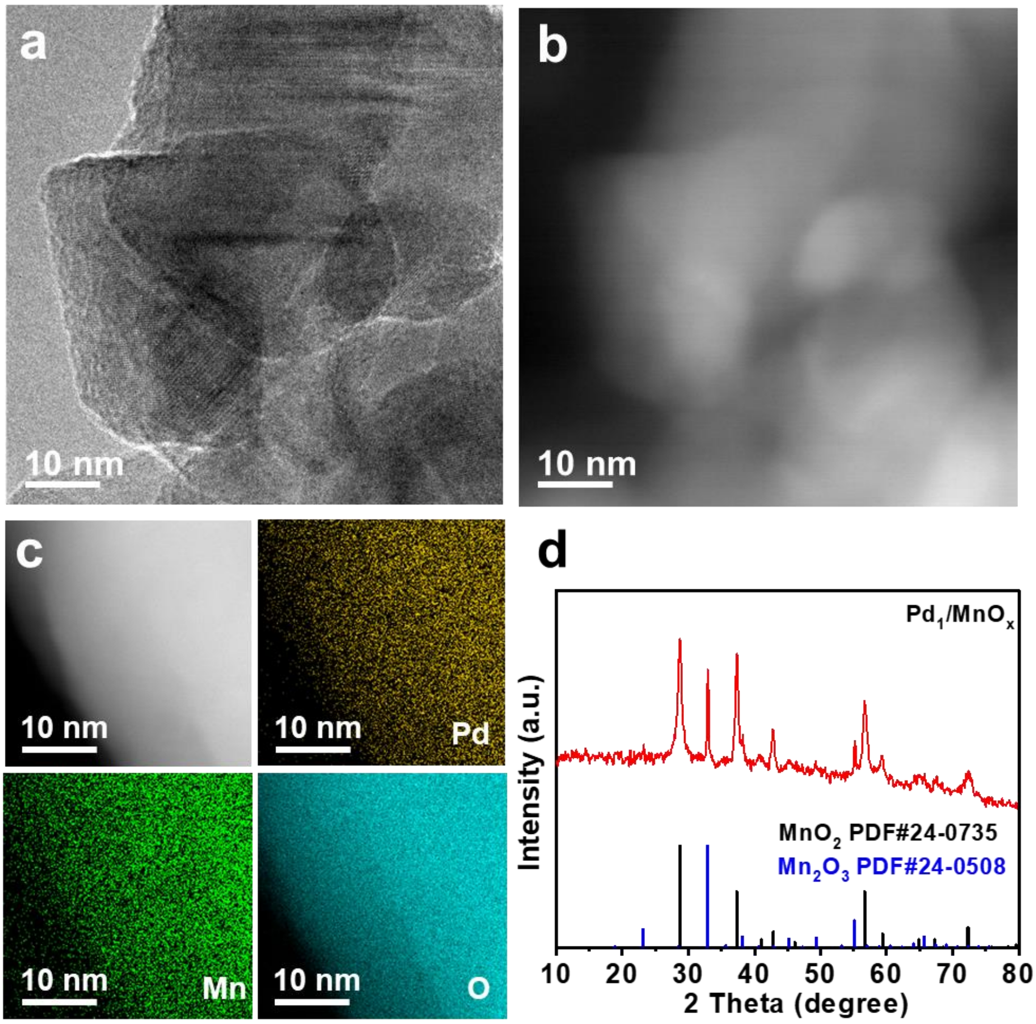

Supplementary Fig. 9 | Structural characterization results of Pd_1/MnO_x. a TEM image, **b** STEM image, **c** elemental mapping, and **d** XRD pattern of Pd_1/MnO_x. Scale bar, 10 nm.

Supplementary Table 6. Pd content and BET surface area for Pd_1/MnO_x

Sample	Pd (wt%) [a]	BET surface area ($m^2\ g^{-1}$) [b]
Pd_1/MnO_x	0.15	27.1

[a] ICP-OES. [b] Sorption isotherm of N_2 at 77 K.

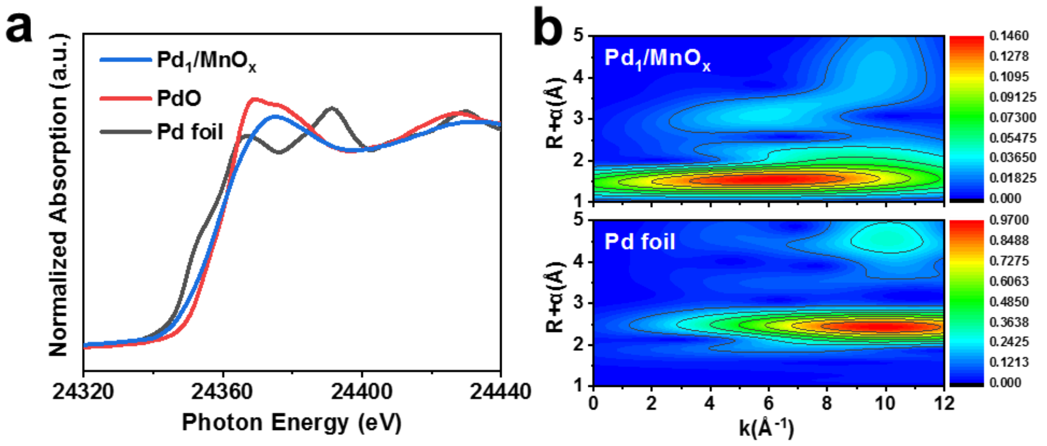

Supplementary Fig. 10 | Structural characterization results of Pd_1/MnO_x. a XANES Pd K-edge for Pd_1/MnO_x, PdO, and Pd foil. **b** WT of Pd K edge EXAFS of Pd_1/MnO_x and Pd foil.

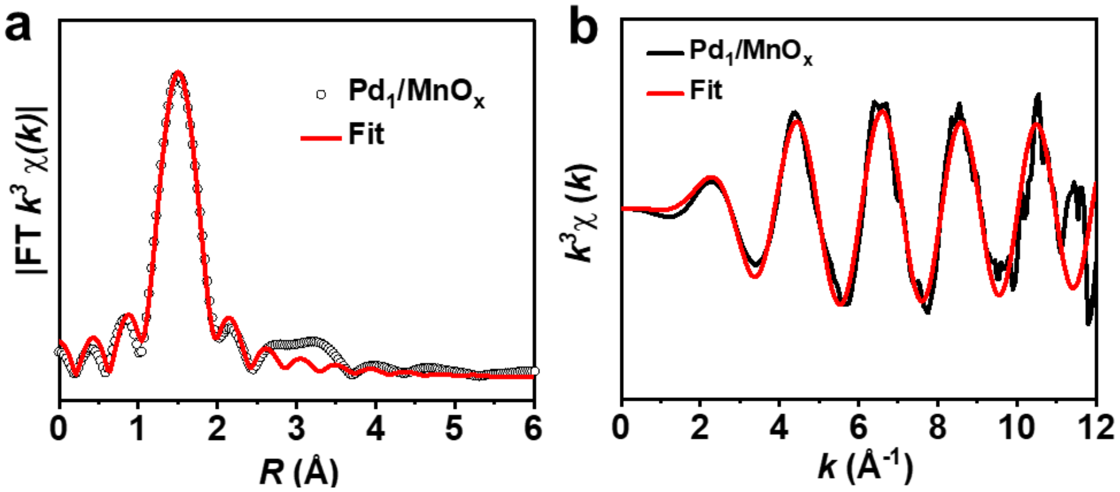

Supplementary Fig. 11 | EXAFS fitting for the Pd_1/MnO_x. **a** FT k^3-weighted $\chi(k)$-function of the EXAFS spectra for Pd K-edge and corresponding R-space fitting curves for the Pd_1/MnO_x catalyst. **b** EXAFS k space fitting curve and the experimental one of Pd_1/MnO_x.

Supplementary Table 7. Structural parameter of EXAFS fitting for the Pd_1/MnO_x

Sample	Scattering pair	CN [a]	R (Å) [b]	σ^2 (× 10^{-3} Å^2) [c]	ΔE_0 (eV) [d]	R factor [e]
Pd_1/MnO_x	Pd-O	3.7±0.4	2.02±0.01	1.5±1.0	1.9±1.5	0.008

[a] CN is the coordination number; [b] R is interatomic distance (the bond length between central atoms and surrounding coordination atoms); [c] σ^2 is Debye-Waller factor (a measure of thermal and static disorder in absorber-scatterer distances); [d] ΔE_0 is edge-energy shift (the difference between the zero kinetic energy value of the sample and that of the theoretical model). [e] R factor is used to value the goodness of the fitting. Data range: $2.8 \leq k \leq 10.0$ Å^{-1}, $1.0 \leq R \leq 2.8$ Å.

Pd_1/ZnO

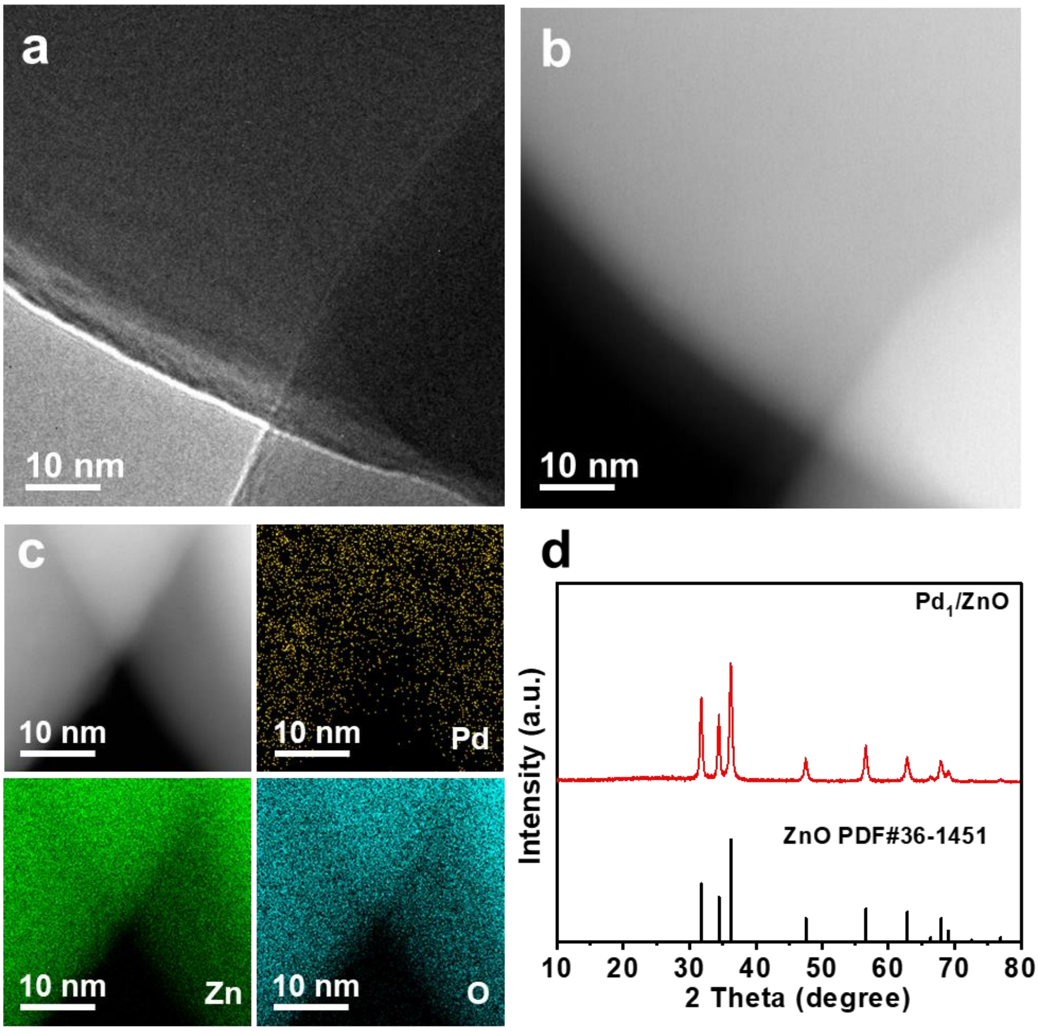

Supplementary Fig. 12 | Structural characterization results of Pd_1/ZnO. **a** TEM image, **b** STEM image, **c** elemental mapping, and **d** XRD pattern of Pd_1/ZnO. Scale bar, 10 nm

.

Supplementary Table 8. Pd content and BET surface area for Pd_1/ZnO

Sample	Pd (wt%) [a]	BET surface area (m^2 g^{-1}) [b]
Pd_1/ZnO	0.21	27.9

[a] ICP-OES. [b] Sorption isotherm of N_2 at 77 K.

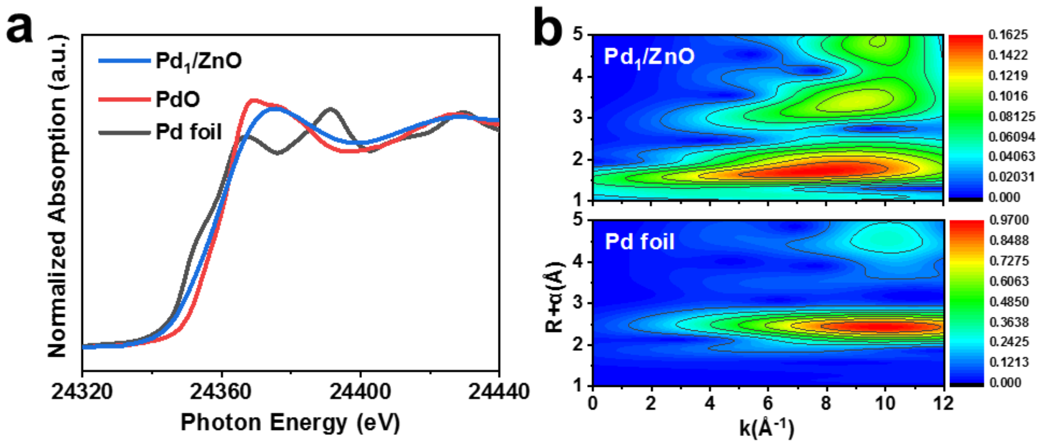

Supplementary Fig. 13 | Structural characterization results of Pd_1/ZnO. a XANES Pd K-edge for Pd_1/ZnO, PdO, and Pd foil. **b** WT of Pd K edge EXAFS of Pd_1/ZnO and Pd foil.

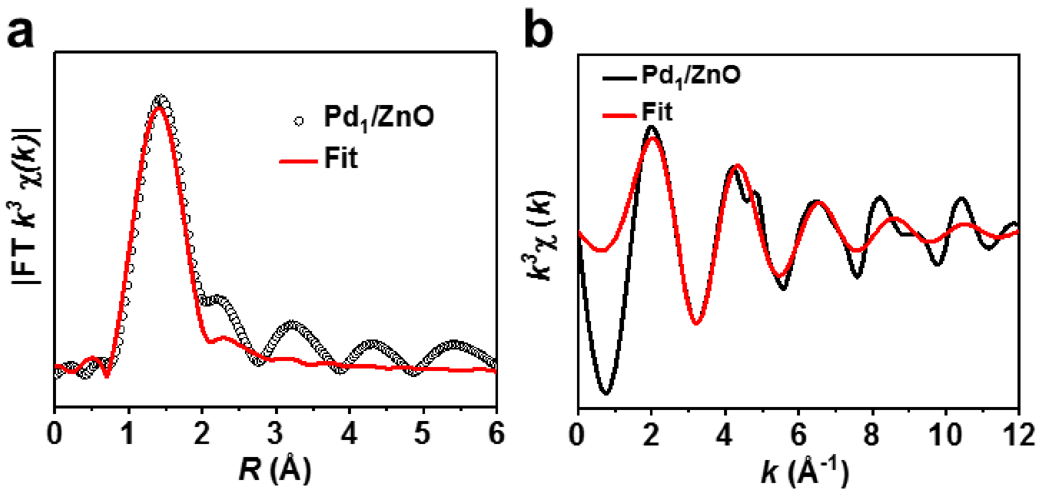

Supplementary Fig. 14 | EXAFS fitting for the Pd_1/FeO_x. **a** FT k^3-weighted $\chi(k)$-function of the EXAFS spectra for Pd K-edge and corresponding R-space fitting curves for the Pd_1/ZnO catalyst. **b** EXAFS *k* space fitting curve and the experimental one of Pd_1/ZnO.

Supplementary Table 9. Structural parameter of EXAFS fitting for the Pd_1/ZnO

Sample	Scattering pair	CN [a]	R (Å) [b]	σ^2 (× 10^{-3} Å^2) [c]	ΔE_0 (eV) [d]	R factor [e]
Pd_1/ZnO	Pd-O	3.5±0.9	2.00±0.03	3.0±5.8	-2.2±2.4	0.02

[a] CN is the coordination number; [b] R is interatomic distance (the bond length between central atoms and surrounding coordination atoms); [c] σ^2 is Debye-Waller factor (a measure of thermal and static disorder in absorber-scatterer distances); [d] ΔE_0 is edge-energy shift (the difference between the zero kinetic energy value of the sample and that of the theoretical model). [e] R factor is used to value the goodness of the fitting. Data range: $2.0 \leq k \leq 8.0$ Å^{-1}, $1.0 \leq R \leq 3.0$ Å.

Pt_1/Al_2O_3

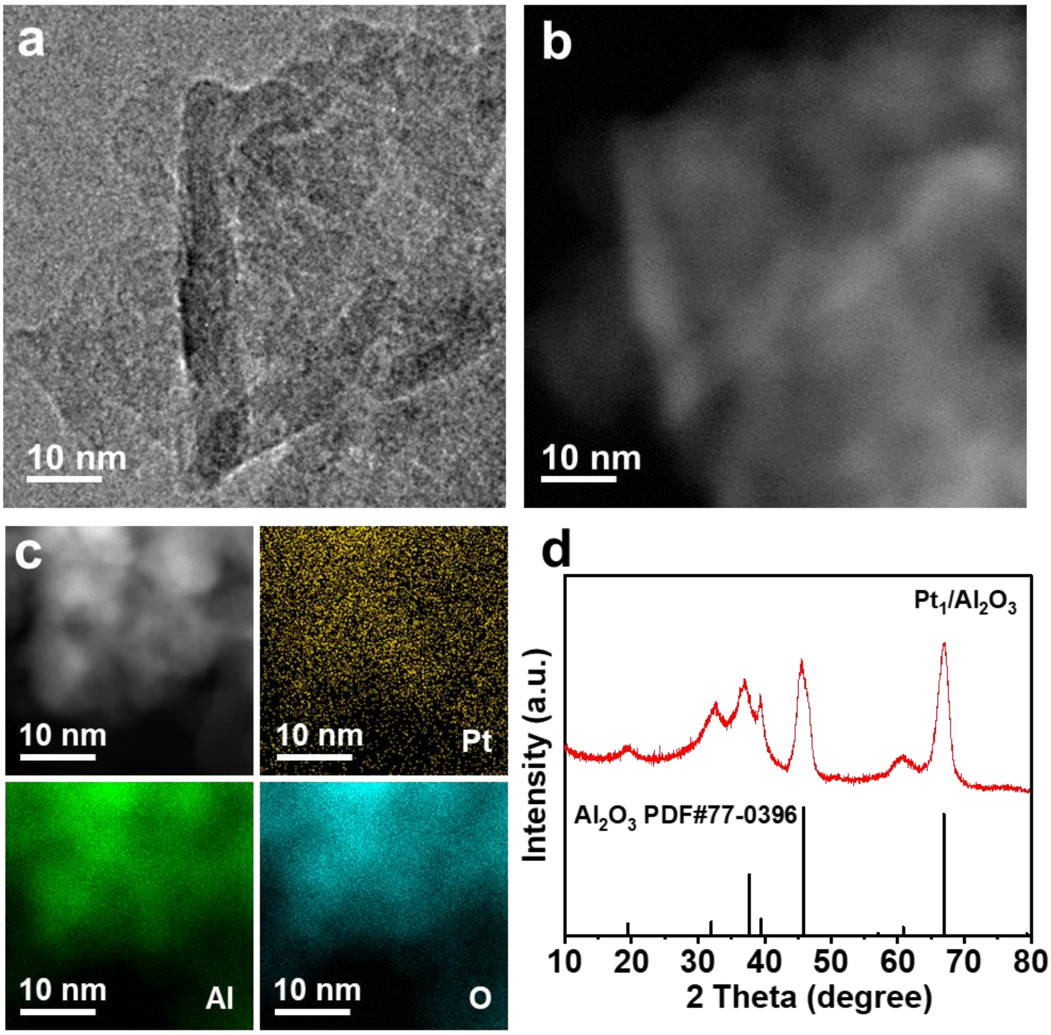

Supplementary Fig. 15 | Structural characterization results of Pt_1/Al_2O_3. a TEM image, **b** STEM image, **c** elemental mapping, and **d** XRD pattern of Pt_1/Al_2O_3. Scale bar, 10 nm.

Supplementary Table 10. Pt content and BET surface area for Pt_1/Al_2O_3

Sample	Pt (wt%) [a]	BET surface area ($m^2\ g^{-1}$) [b]
Pt_1/Al_2O_3	0.15	124.7

[a] ICP-OES. [b] Sorption isotherm of N_2 at 77 K.

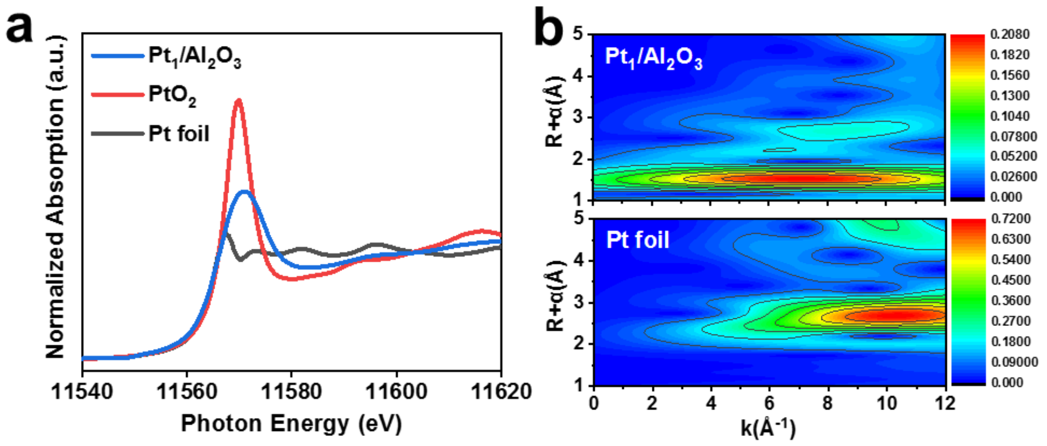

Supplementary Fig. 16 | Structural characterization results of Pt_1/Al_2O_3. a XANES Pt L_3-edge for Pt_1/Al_2O_3, PtO_2, and Pt foil. **b** WT of Pt L_3-edge EXAFS of Pt_1/Al_2O_3 and Pt foil.

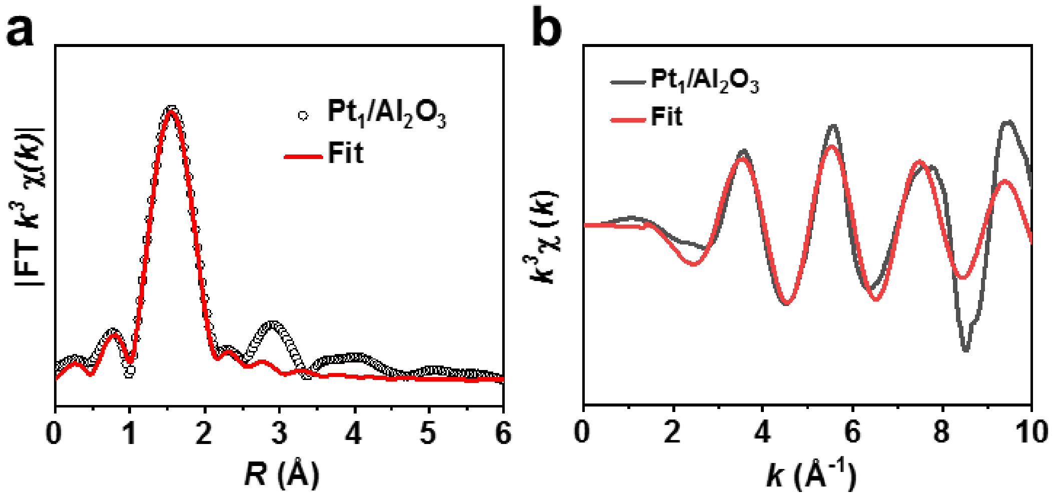

Supplementary Fig. 17 | EXAFS fitting for the Pt_1/Al_2O_3. **a** FT k^3-weighted $\chi(k)$-function of the EXAFS spectra for Pt L_3-edge and corresponding R-space fitting curves for the Pt_1/Al_2O_3 catalyst. **b** EXAFS k space fitting curve and the experimental one of Pt_1/Al_2O_3.

Supplementary Table 11. Structural parameter of EXAFS fitting for the Pt_1/Al_2O_3

Sample	Scattering pair	CN [a]	R (Å) [b]	σ^2 (× 10^{-3} Å^2) [c]	ΔE_0 (eV) [d]	R factor [e]
Pt_1/Al_2O_3	Pt-O	4.1±1.3	2.01±0.03	5.5±4.4	7.4±3.5	0.03

[a] CN is the coordination number; [b] R is interatomic distance (the bond length between central atoms and surrounding coordination atoms); [c] σ^2 is Debye-Waller factor (a measure of thermal and static disorder in absorber-scatterer distances); [d] ΔE_0 is edge-energy shift (the difference between the zero kinetic energy value of the sample and that of the theoretical model). [e] R factor is used to value the goodness of the fitting. Data range: $2.0 \leq k \leq 8.4$ Å^{-1}, $1.0 \leq R \leq 2.6$ Å.

Pt_1/TiO_2

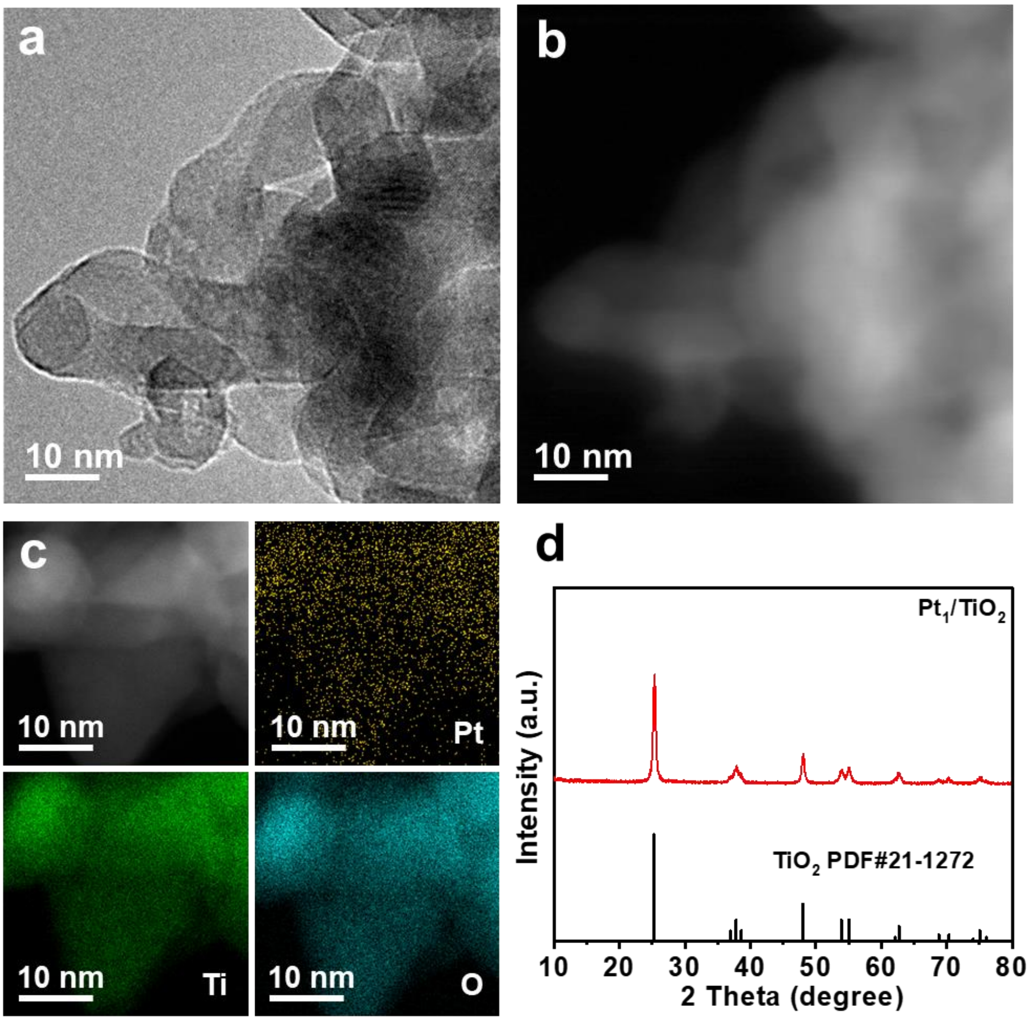

Supplementary Fig. 18 | Structural characterization results of Pt_1/TiO_2. a TEM image, **b** STEM image, **c** elemental mapping, and **d** XRD pattern of Pt_1/TiO_2. Scale bar, 10 nm.

Supplementary Table 12. Pt content and BET surface area for Pt_1/TiO_2

Sample	Pt (wt%) [a]	BET surface area (m^2 g^{-1}) [b]
Pt_1/TiO_2	0.14	82.4

[a] ICP-OES. [b] Sorption isotherm of N_2 at 77 K.

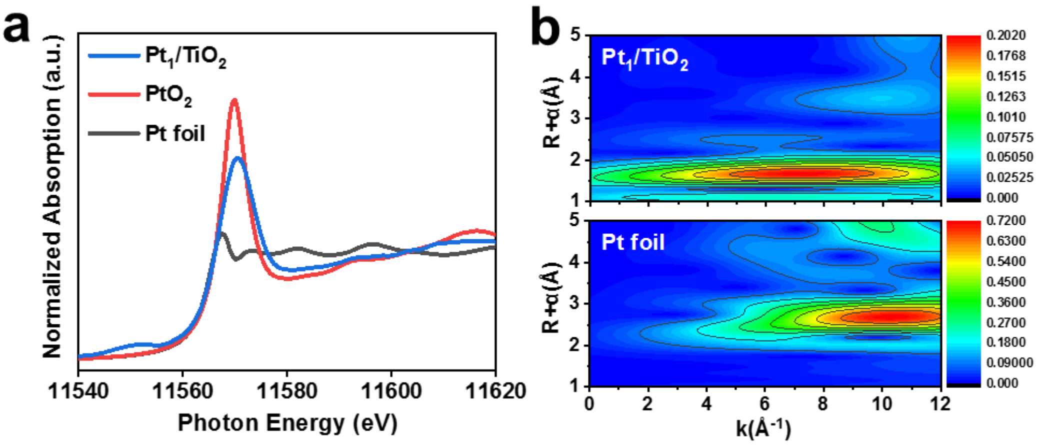

Supplementary Fig. 19 | Structural characterization results of Pt_1/TiO_2. a XANES Pt L_3-edge K-edge for Pt_1/TiO_2, PtO_2, and Pt foil. **b** WT of Pt L_3-edge EXAFS of Pt_1/TiO_2 and Pt foil.

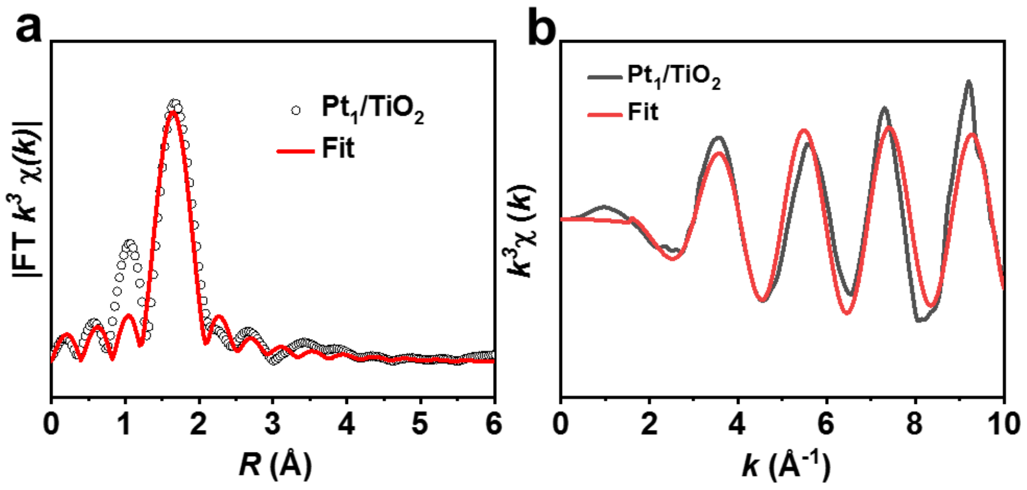

Supplementary Fig. 20 | EXAFS fitting for the Pt_1/TiO_2. **a** FT k^3-weighted $\chi(k)$-function of the EXAFS spectra for Pt L_3-edge and corresponding R-space fitting curves for the Pt_1/TiO_2 catalyst. **b** EXAFS *k* space fitting curve and the experimental one of Pt_1/TiO_2.

Supplementary Table 13. Structural parameter of EXAFS fitting for the Pt_1/TiO_2

Sample	Scattering pair	CN [a]	R (Å) [b]	σ^2 (× 10^{-3} Å^2) [c]	ΔE_0 (eV) [d]	R factor [e]
Pt_1/TiO_2	Pt–O	3.6±0.9	2.04±0.03	0.7±3.8	9.5±3.1	0.03

[a] CN is the coordination number; [b] R is interatomic distance (the bond length between central atoms and surrounding coordination atoms); [c] σ^2 is Debye-Waller factor (a measure of thermal and static disorder in absorber-scatterer distances); [d] ΔE_0 is edge-energy shift (the difference between the zero kinetic energy value of the sample and that of the theoretical model). [e] R factor is used to value the goodness of the fitting. Data range: $3.0 \leq k \leq 10.5$ Å^{-1}, $1.3 \leq R \leq 2.7$ Å.

Pt_1/MnO_x

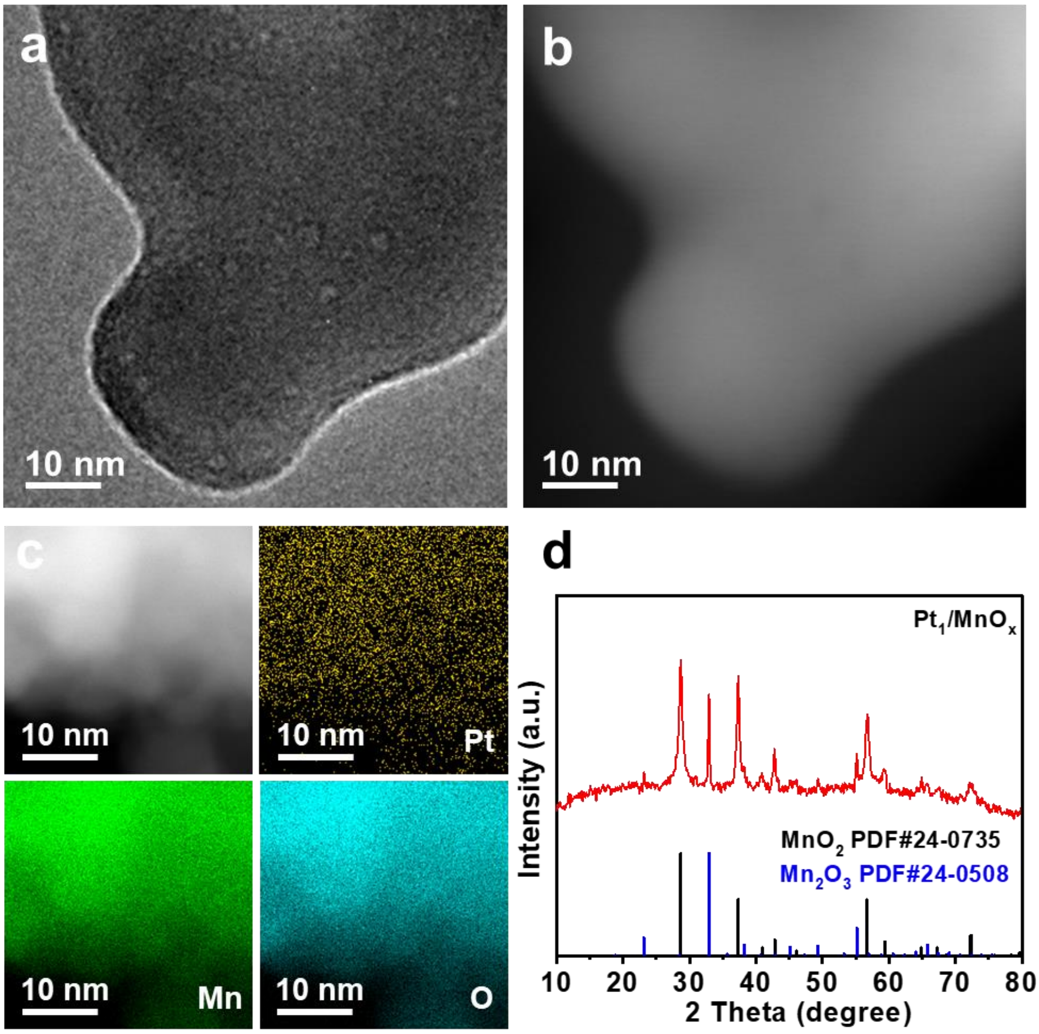

Supplementary Fig. 21 | Structural characterization results of Pt_1/MnO_x. **a** TEM image, **b** STEM image, **c** elemental mapping, and **d** XRD pattern of Pt_1/MnO_x. Scale bar, 10 nm.

Supplementary Table 14. Pt content and BET surface area for Pt_1/MnO_x

Sample	Pt (wt%) [a]	BET surface area ($m^2\ g^{-1}$) [b]
Pt_1/MnO_x	0.19	30.3

[a] ICP-OES. [b] Sorption isotherm of N_2 at 77 K.

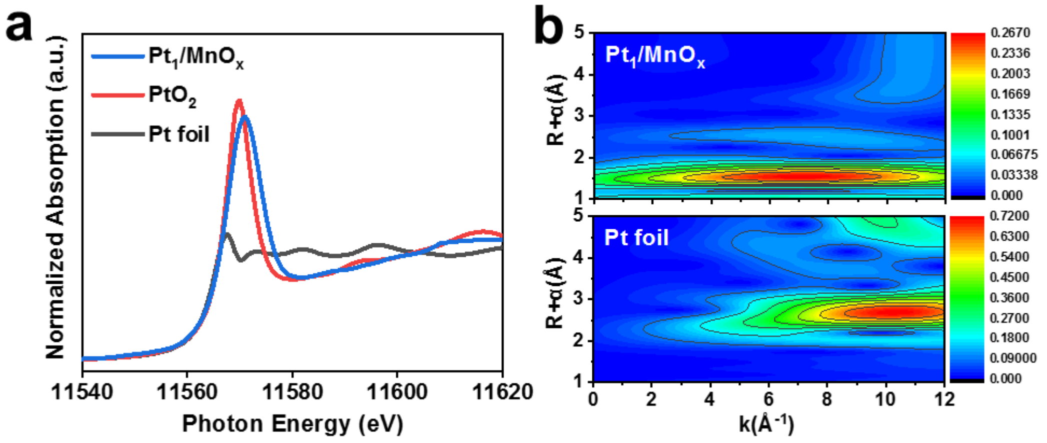

Supplementary Fig. 22 | Structural characterization results of Pt_1/MnO_x. a XANES Pt L_3-edge K-edge for Pt_1/MnO_x, PtO_2, and Pt foil. **b** WT of Pt L_3-edge EXAFS of Pt_1/MnO_x and Pt foil.

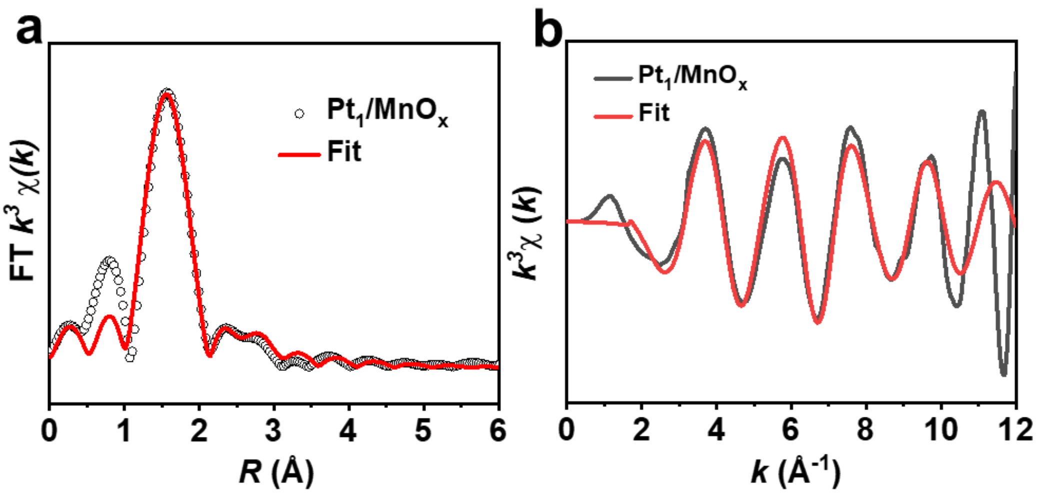

Supplementary Fig. 23 | EXAFS fitting for the Pt_1/MnO_x. **a** FT k^3-weighted $\chi(k)$-function of the EXAFS spectra for Pt L_3-edge and corresponding R-space fitting curves for the Pt_1/MnO_x catalyst. **b** EXAFS k space fitting curve and the experimental one of Pt_1/MnO_x.

Supplementary Table 15. Structural parameter of EXAFS fitting for the Pt_1/MnO_x

Sample	Scattering pair	CN [a]	R (Å) [b]	σ^2 (× 10^{-3} Å^2) [c]	ΔE_0 (eV) [d]	R factor [e]
Pt_1/MnO_x	Pt-O	4.4±0.4	1.98±0.01	2.6±1.1	9.6±1.0	0.003
	Pt-O-Mn	1.4±1.1	2.96±0.02	11.4±8.3		

[a] CN is the coordination number; [b] R is interatomic distance (the bond length between central atoms and surrounding coordination atoms); [c] σ^2 is Debye-Waller factor (a measure of thermal and static disorder in absorber-scatterer distances); [d] ΔE_0 is edge-energy shift (the difference between the zero kinetic energy value of the sample and that of the theoretical model). [e] R factor is used to value the goodness of the fitting. Data range: $3.1 \le k \le 9.3$ Å^{-1}, $1.3 \le R \le 4.0$ Å.

Pt_1/FeO_x

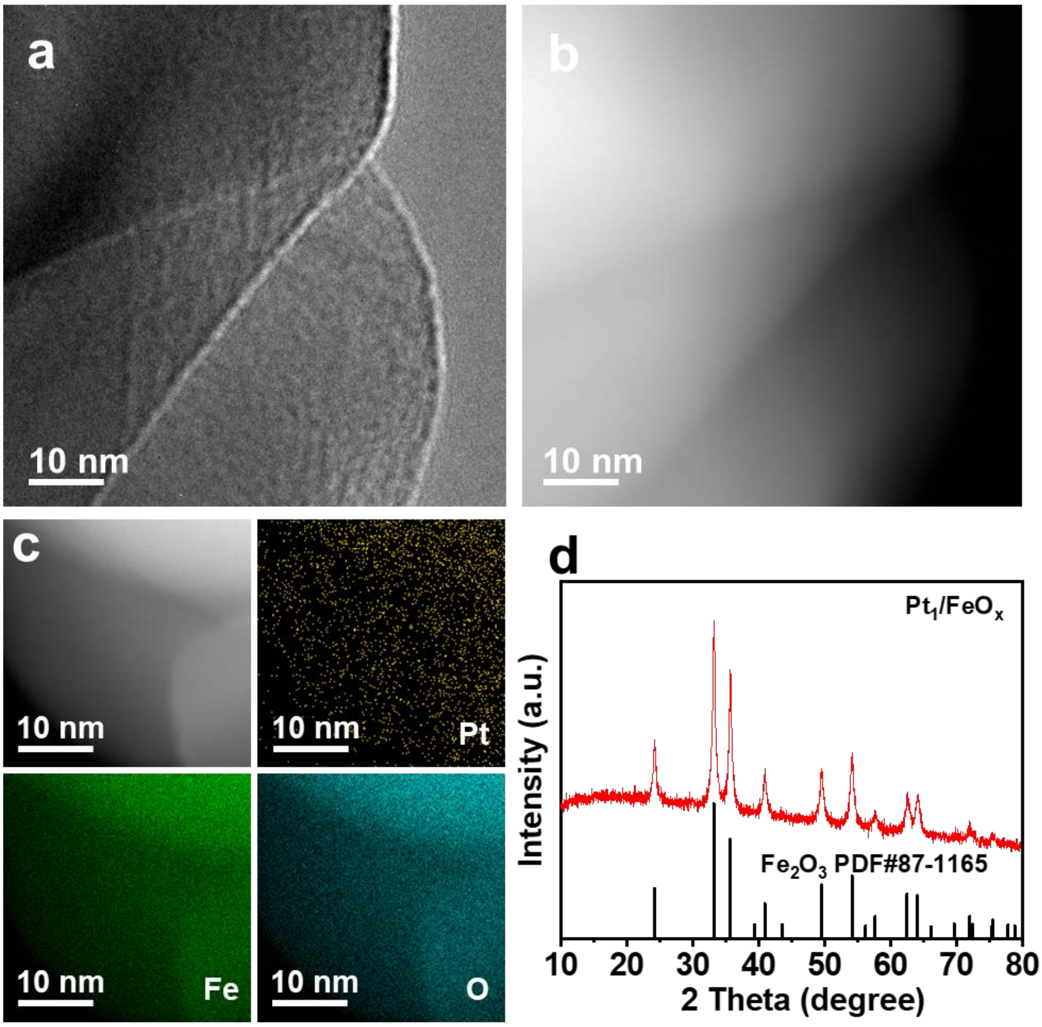

Supplementary Fig. 24 | Structural characterization results of Pt_1/FeO_x. a TEM image, **b** STEM image, **c** elemental mapping, and **d** XRD pattern of Pt_1/FeO_x. Scale bar, 10 nm.

Supplementary Table 16. Pt content and BET surface area for Pt_1/FeO_x

Sample	Pt (wt%) [a]	BET surface area ($m^2\ g^{-1}$) [b]
Pt_1/FeO_x	0.37	38.1

[a] ICP-OES. [b] Sorption isotherm of N_2 at 77 K.

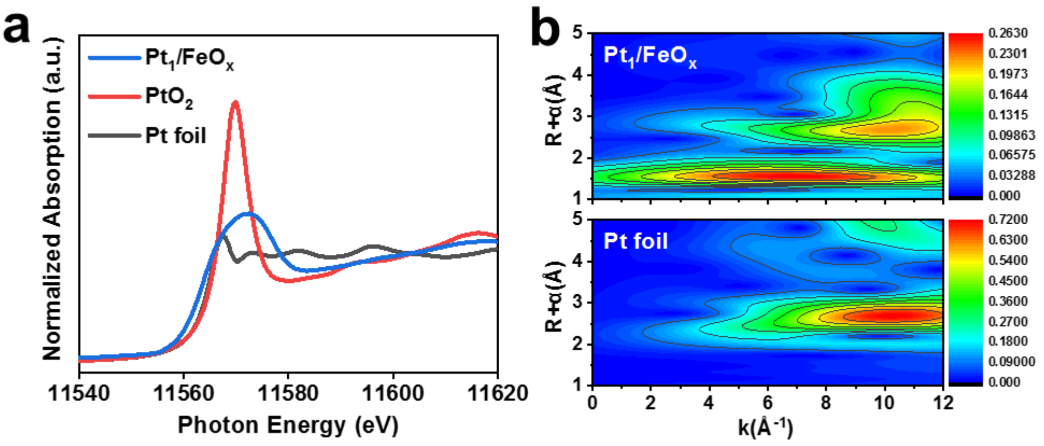

Supplementary Fig. 25 | Structural characterization results of Pt_1/FeO_x. a XANES Pt L_3-edge K-edge for Pt_1/FeO_x, PtO_2, and Pt foil. **b** WT of Pt L_3-edge EXAFS of Pt_1/FeO_x and Pt foil.

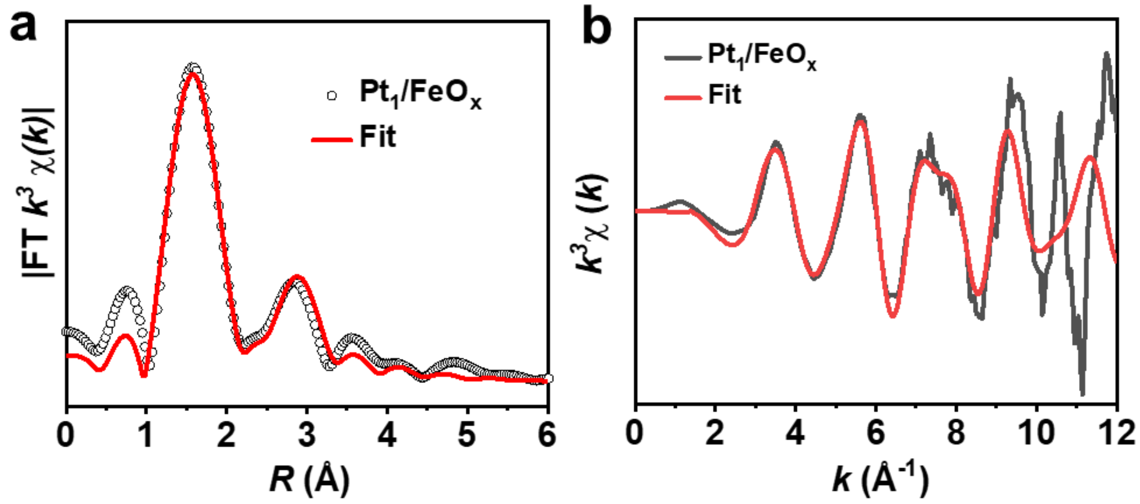

Supplementary Fig. 26 | EXAFS fitting for the Pt_1/FeO_x. **a** FT k^3-weighted $\chi(k)$-function of the EXAFS spectra for Pt L_3-edge and corresponding R-space fitting curves for the Pt_1/FeO_x catalyst. **b** EXAFS *k* space fitting curve and the experimental one of Pt_1/FeO_x.

Supplementary Table 17. Structural parameter of EXAFS fitting for the Pt_1/FeO_x

Sample	Scattering pair	CN [a]	R (Å) [b]	σ^2 ($\times 10^{-3}$ Å^2) [c]	ΔE_0 (eV) [d]	R factor [e]
Pt_1/FeO_x	Pt-O	4.4±0.6	2.01±0.01	3.1±1.8	7.5±1.7	0.007
	Pt-O-Fe	2.4±1.4	3.09±0.02	6.9±5.9		

[a] CN is the coordination number; [b] R is interatomic distance (the bond length between central atoms and surrounding coordination atoms); [c] σ^2 is Debye-Waller factor (a measure of thermal and static disorder in absorber-scatterer distances); [d] ΔE_0 is edge-energy shift (the difference between the zero kinetic energy value of the sample and that of the theoretical model). [e] R factor is used to value the goodness of the fitting. Data range: $3.5 \leq k \leq 9.0$ Å^{-1}, $1.1 \leq R \leq 4.0$ Å.

Pt_1/ZnO

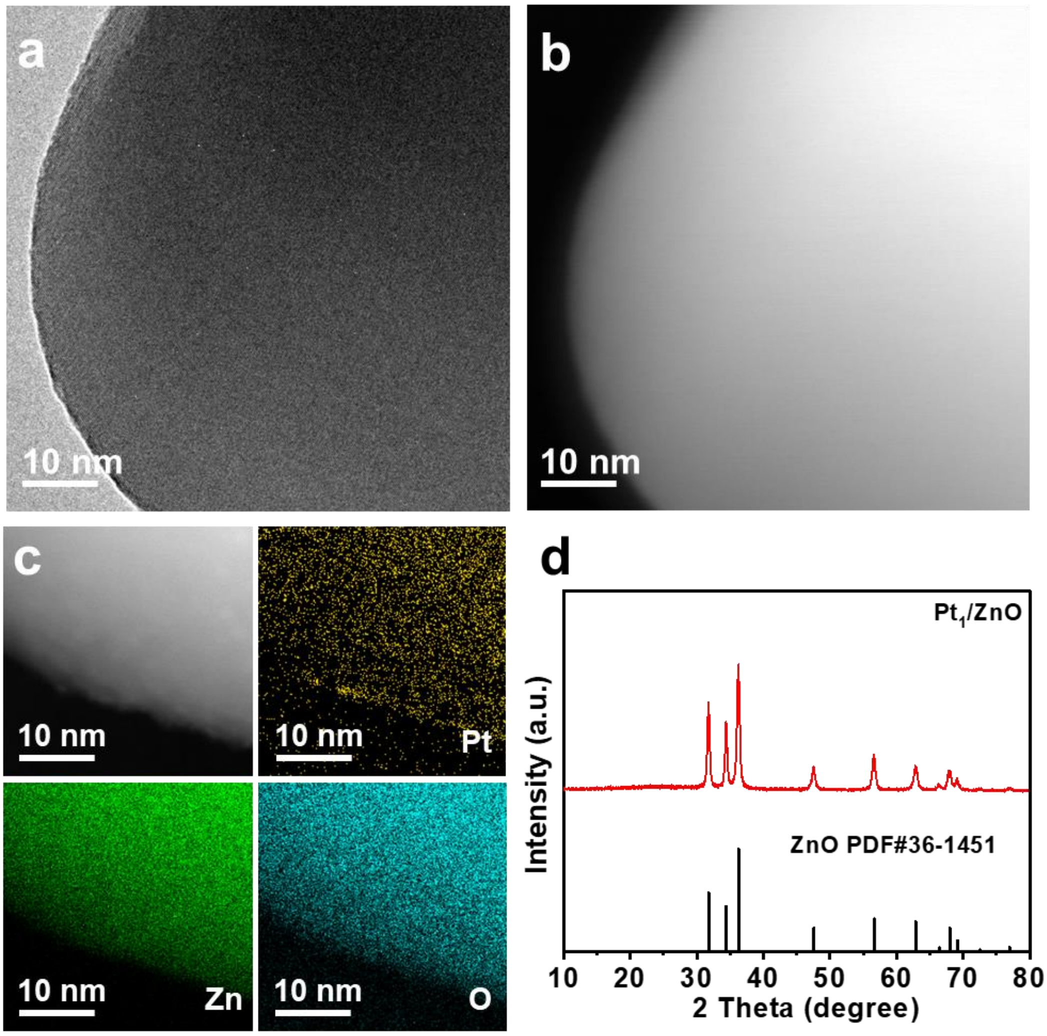

Supplementary Fig. 27 | Structural characterization results of Pt_1/ZnO. **a** TEM image, **b** STEM image, **c** elemental mapping, and **d** XRD pattern of Pt_1/ZnO. Scale bar, 10 nm.

Supplementary Table 18. Pt content and BET surface area for Pt_1/ZnO

Sample	Pt (wt%) [a]	BET surface area ($m^2\ g^{-1}$) [b]
Pt_1/ZnO	0.11	27.5

[a] ICP-OES. [b] Sorption isotherm of N_2 at 77 K.

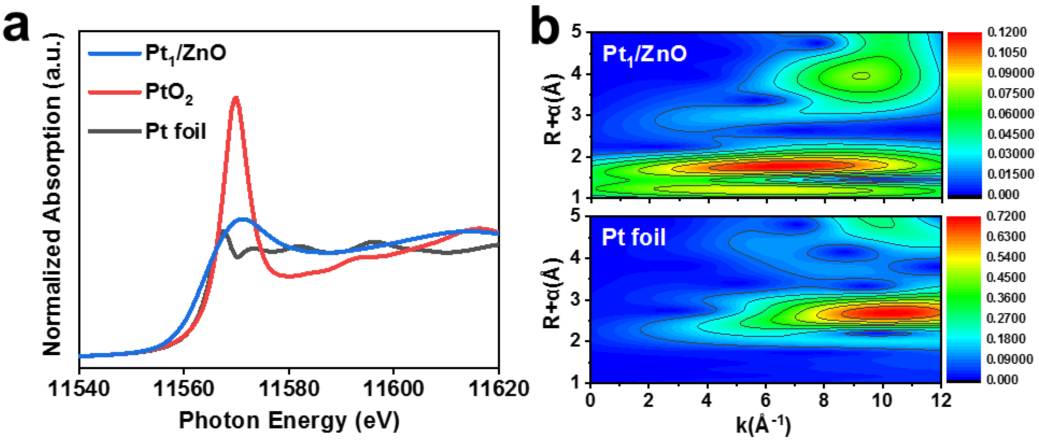

Supplementary Fig. 28 | Structural characterization results of Pt_1/ZnO. **a** XANES Pt L_3-edge K-edge for Pt_1/ZnO, PtO_2, and Pt foil. **b** WT of Pt L_3-edge EXAFS of Pt_1/ZnO and Pt foil.

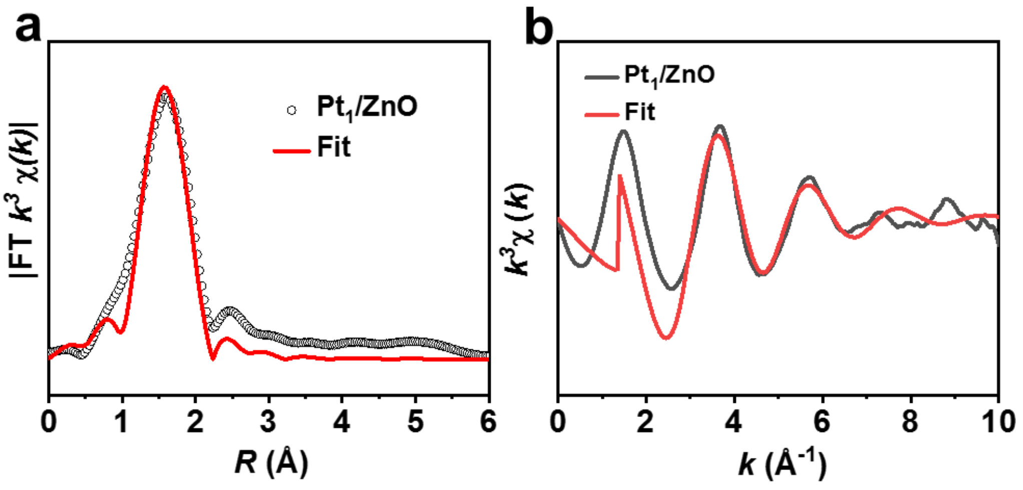

Supplementary Fig. 29 | EXAFS fitting for the Pt_1/ZnO. **a** FT k^3-weighted $\chi(k)$-function of the EXAFS spectra for Pt L_3-edge and corresponding R-space fitting curves for the Pt_1/ZnO catalyst. **b** EXAFS k space fitting curve and the experimental one of Pt_1/ZnO.

Supplementary Table 19. Structural parameter of EXAFS fitting for the Pt_1/ZnO

Sample	Scattering pair	CN [a]	R (Å) [b]	σ^2 (× 10^{-3} Å^2) [c]	ΔE_0 (eV) [d]	R factor [e]
Pt_1/ZnO	Pt–O	4.0±0.8	2.04±0.02	9.7±4.2	8.1±2.1	0.02

[a] CN is the coordination number; [b] R is interatomic distance (the bond length between central atoms and surrounding coordination atoms); [c] σ^2 is Debye-Waller factor (a measure of thermal and static disorder in absorber-scatterer distances); [d] ΔE_0 is edge-energy shift (the difference between the zero kinetic energy value of the sample and that of the theoretical model). [e] R factor is used to value the goodness of the fitting. Data range: $2.1 \leq k \leq 8.2$ Å^{-1}, $1.1 \leq R \leq 2.7$ Å.

Ru_1/Al_2O_3

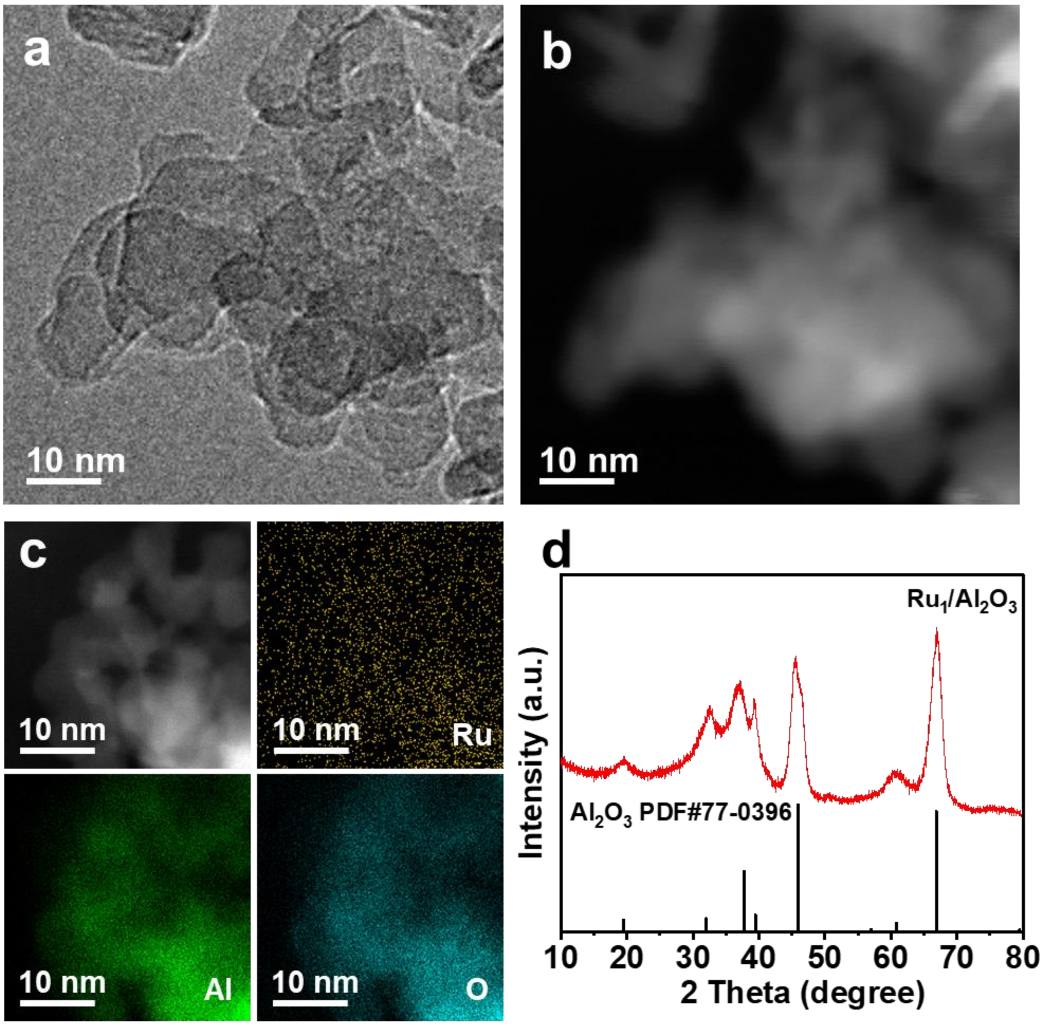

Supplementary Fig. 30 | Structural characterization results of Ru_1/Al_2O_3. **a** TEM image, **b** STEM image, **c** elemental mapping, and **d** XRD pattern of Ru_1/Al_2O_3. Scale bar, 10 nm

Supplementary Table 20. Ru content and BET surface area for Ru_1/Al_2O_3

Sample	Ru (wt%) [a]	BET surface area ($m^2\ g^{-1}$) [b]
Ru_1/Al_2O_3	0.12	116.4

[a] ICP-OES. [b] Sorption isotherm of N_2 at 77 K.

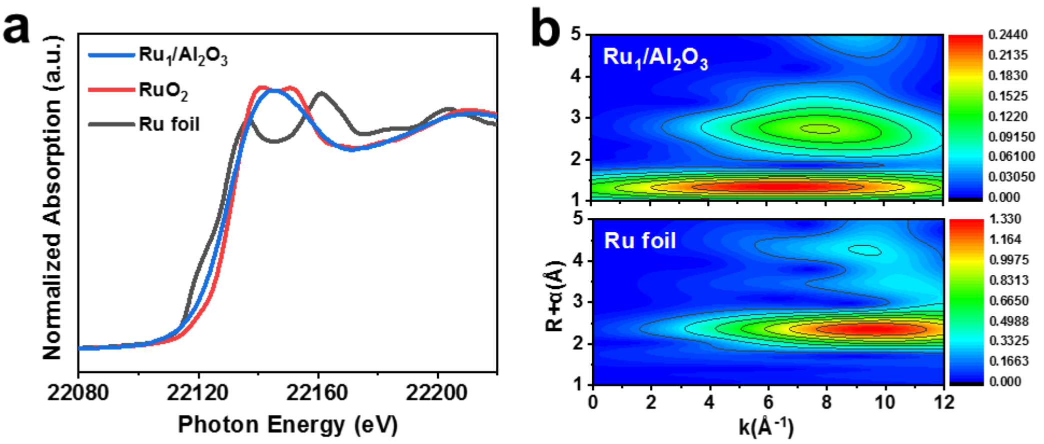

Supplementary Fig. 31 | Structural characterization results of Ru_1/Al_2O_3. a XANES Ru K-edge for Ru_1/Al_2O_3, RuO_2, and Ru foil. **b** WT of Ru K-edge EXAFS of Ru_1/Al_2O_3 and Ru foil.

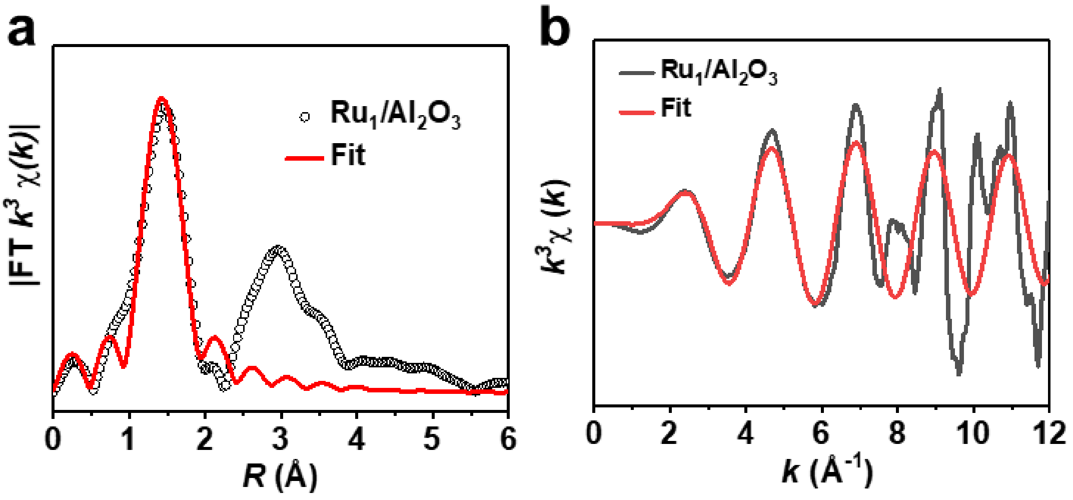

Supplementary Fig. 32 | EXAFS fitting for the Ru_1/Al_2O_3. **a** FT k^3-weighted $\chi(k)$-function of the EXAFS spectra for Ru K-edge and corresponding R-space fitting curves for the Ru_1/Al_2O_3 catalyst. **b** EXAFS k space fitting curve and the experimental one of Ru_1/Al_2O_3.

Supplementary Table 21. Structural parameter of EXAFS fitting for the Ru_1/Al_2O_3

Sample	Scattering pair	CN [a]	R (Å) [b]	σ^2 (× 10^{-3} Å^2) [c]	ΔE_0 (eV) [d]	R factor [e]
Ru_1/Al_2O_3	Ru-O	4.2±1.2	1.96±0.02	1.6±4.0	-1.6±3.0	0.02

[a] CN is the coordination number; [b] R is interatomic distance (the bond length between central atoms and surrounding coordination atoms); [c] σ^2 is Debye-Waller factor (a measure of thermal and static disorder in absorber-scatterer distances); [d] ΔE_0 is edge-energy shift (the difference between the zero kinetic energy value of the sample and that of the theoretical model). [e] R factor is used to value the goodness of the fitting. Data range: $2.8 \leq k \leq 9.2$ Å^{-1}, $1.2 \leq R \leq 2.7$ Å.

Ru_1/TiO_2

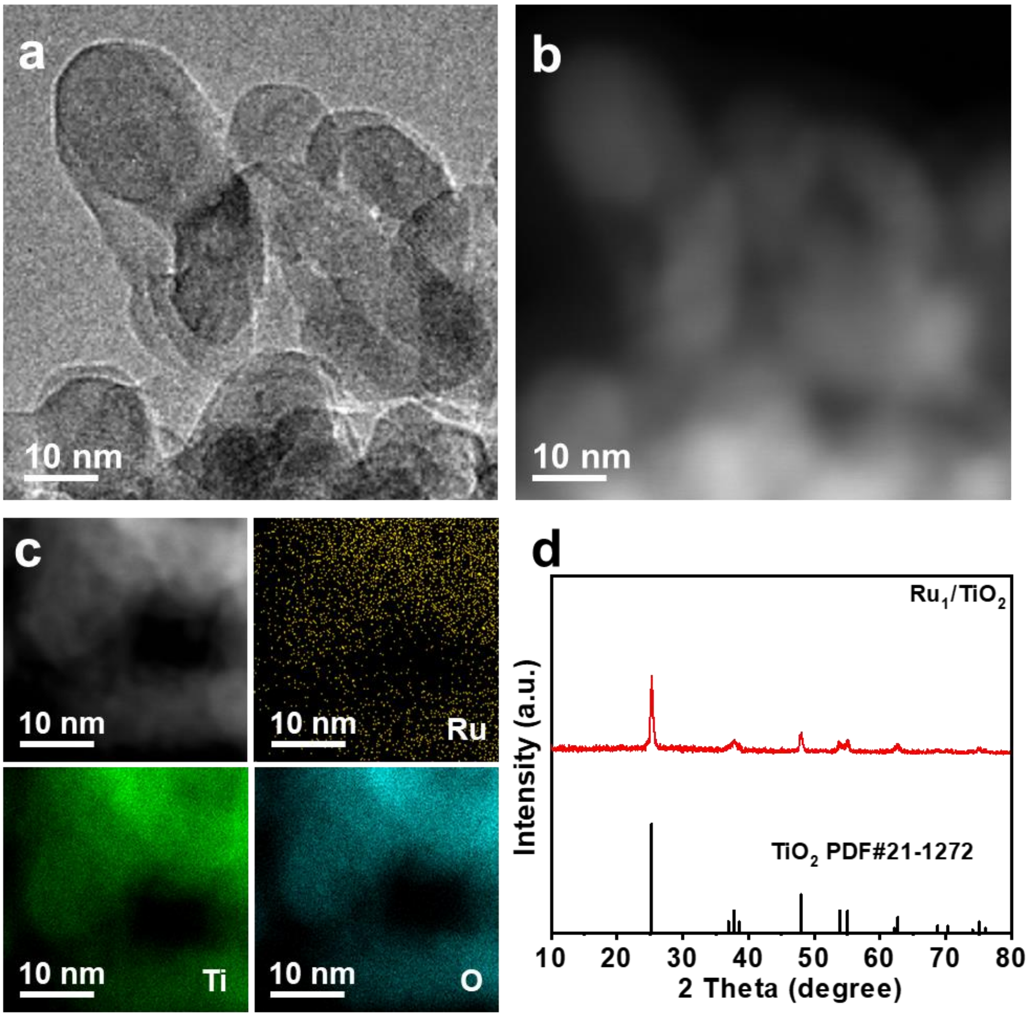

Supplementary Fig. 33 | Structural characterization results of Ru_1/TiO_2. a TEM image, **b** STEM image, **c** elemental mapping, and **d** XRD pattern of Ru_1/TiO_2. Scale bar, 10 nm

Supplementary Table 22. Ru content and BET surface area for Ru_1/TiO_2

Sample	Ru (wt%) [a]	BET surface area (m^2 g^{-1}) [b]
Ru_1/TiO_2	0.11	80.4

[a] ICP-OES. [b] Sorption isotherm of N_2 at 77 K

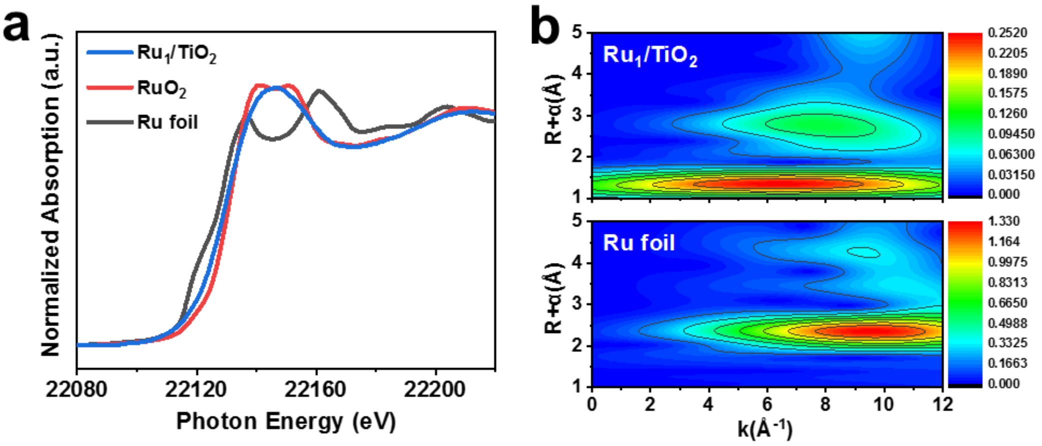

Supplementary Fig. 34 | Structural characterization results of Ru_1/TiO_2. a

XANES Ru K-edge for Ru_1/TiO_2, RuO_2, and Ru foil. **b** WT of Ru K-edge EXAFS of Ru_1/TiO_2 and Ru foil.

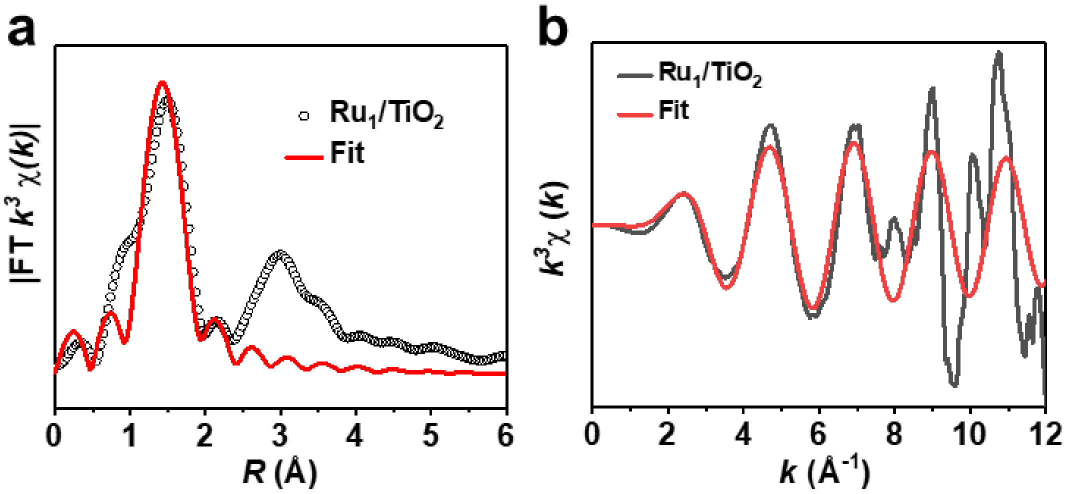

Supplementary Fig. 35 | EXAFS fitting for the Ru_1/TiO_2. **a** FT k^3-weighted $\chi(k)$-function of the EXAFS spectra for Ru K-edge and corresponding R-space fitting curves for the Ru_1/TiO_2 catalyst. **b** EXAFS k space fitting curve and the experimental one of Ru_1/TiO_2.

Supplementary Table 23. Structural parameter of EXAFS fitting for the Ru_1/TiO_2

Sample	Scattering pair	CN [a]	R (Å) [b]	σ^2 (× 10^{-3} Å^2) [c]	ΔE_0 (eV) [d]	R factor [e]
Ru_1/TiO_2	Ru-O	4.2±0.9	1.96±0.02	1.8±3.1	-2.6±2.3	0.01

[a] CN is the coordination number; [b] R is interatomic distance (the bond length between central atoms and surrounding coordination atoms); [c] σ^2 is Debye-Waller factor (a measure of thermal and static disorder in absorber-scatterer distances); [d] ΔE_0 is edge-energy shift (the difference between the zero kinetic energy value of the sample and that of the theoretical model). [e] R factor is used to value the goodness of the fitting. Data range: $2.8 \leq k \leq 9.5$ Å^{-1}, $1.1 \leq R \leq 2.6$ Å.

Ru_1/MnO_x

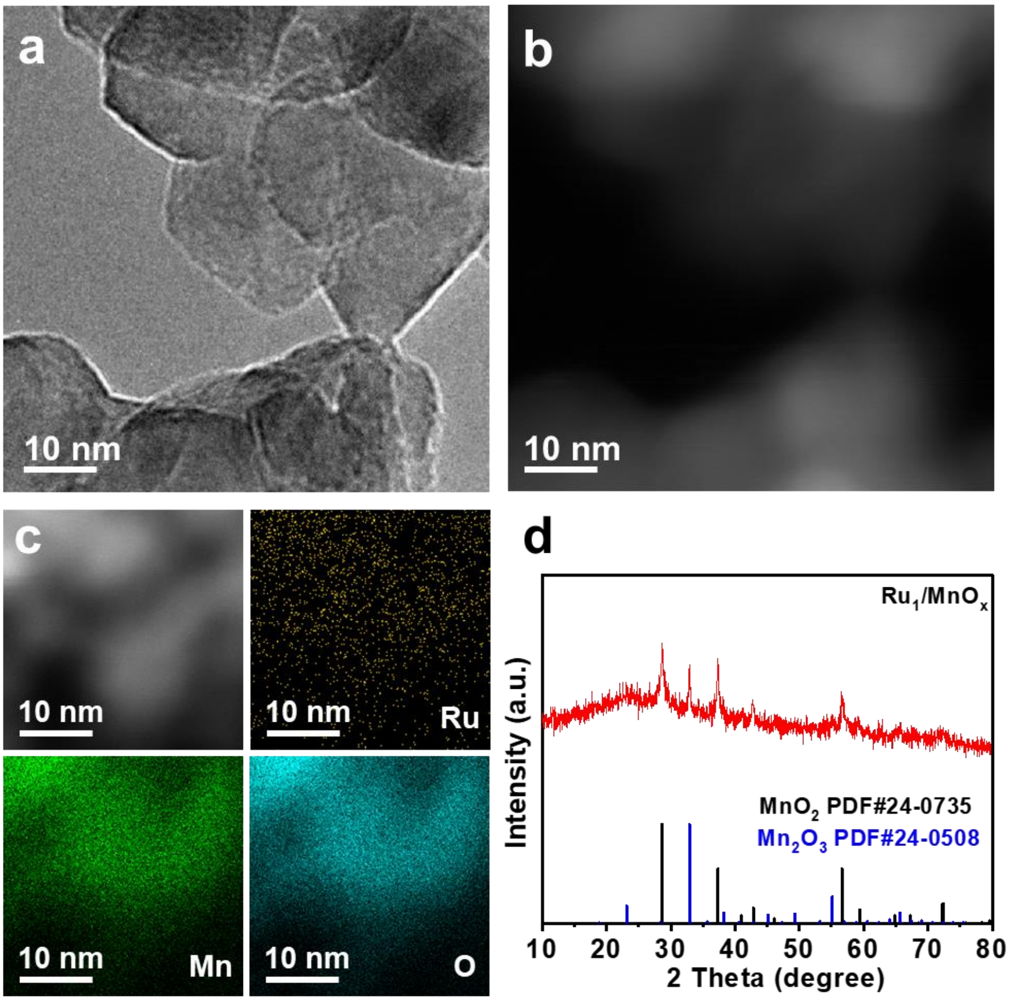

Supplementary Fig. 36 | Structural characterization results of Ru_1/MnO_x. a TEM image, **b** STEM image, **c** elemental mapping, and **d** XRD pattern of Ru_1/MnO_x. Scale bar, 10 nm

Supplementary Table 24. Ru content and BET surface area for Ru_1/MnO_x

Sample	Ru (wt%) [a]	BET surface area ($m^2\ g^{-1}$) [b]
Ru_1/MnO_x	0.11	22.7

[a] ICP-OES. [b] Sorption isotherm of N_2 at 77 K.

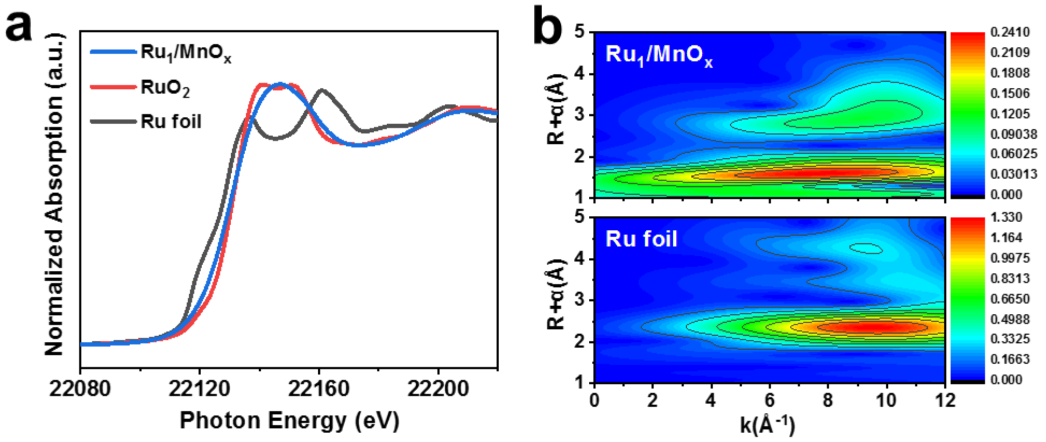

Supplementary Fig. 37 | Structural characterization results of Ru_1/MnO_x. a XANES Ru K-edge for Ru_1/MnO_x, RuO_2, and Ru foil. **b** WT of Ru K-edge EXAFS of Ru_1/MnO_x and Ru foil.

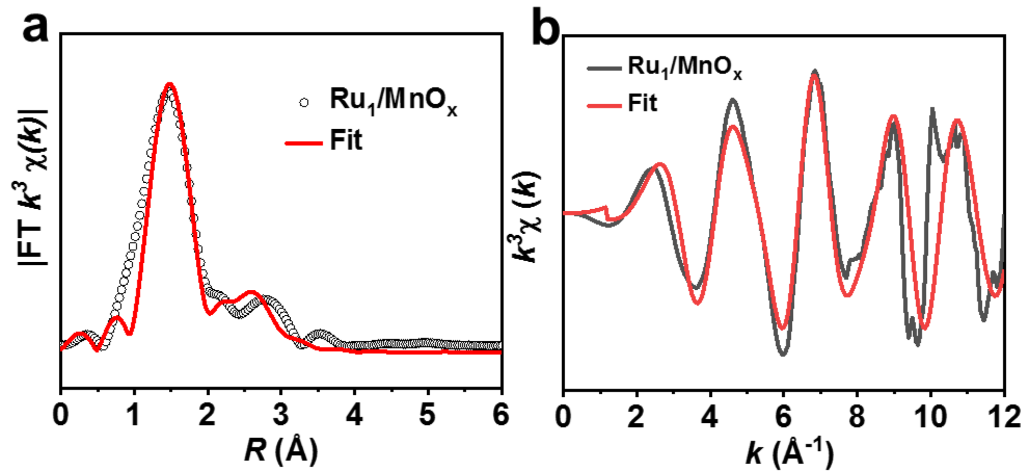

Supplementary Fig. 38 | EXAFS fitting for the Ru_1/MnO_x. **a** FT k^3-weighted $\chi(k)$-function of the EXAFS spectra for Ru K-edge and corresponding R-space fitting curves for the Ru_1/MnO_x catalyst. **b** EXAFS k space fitting curve and the experimental one of Ru_1/MnO_x.

Supplementary Table 25. Structural parameter of EXAFS fitting for the Ru_1/MnO_x

Sample	Scattering pair	CN [a]	R (Å) [b]	σ^2 (× 10^{-3} $Å^2$) [c]	ΔE_0 (eV) [d]	R factor [e]
Ru_1/MnO_x	Ru-O	4.1±1.0	1.99±0.01	0.9±2.6	5.3±2.8	0.02
	Ru-O-Mn	2.4±2.3	2.99±0.03	9.3±9.5		

[a] CN is the coordination number; [b] R is interatomic distance (the bond length between central atoms and surrounding coordination atoms); [c] σ^2 is Debye-Waller factor (a measure of thermal and static disorder in absorber-scatterer distances); [d] ΔE_0 is edge-energy shift (the difference between the zero kinetic energy value of the sample and that of the theoretical model). [e] R factor is used to value the goodness of the fitting. Data range: $2.8 \leq k \leq 9.2$ $Å^{-1}$, $1.2 \leq R \leq 3.8$ Å.

Ru_1/FeO_x

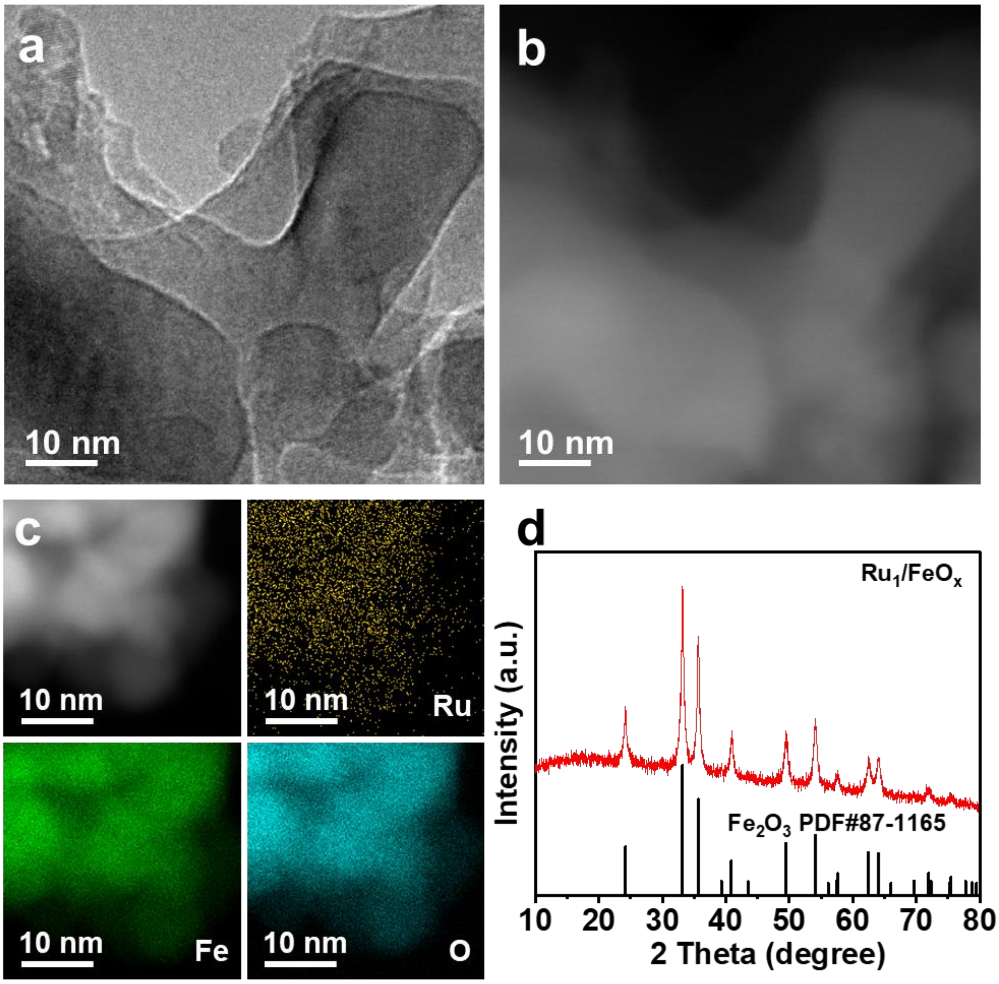

Supplementary Fig. 39 | Structural characterization results of Ru_1/FeO_x. **a** TEM image, **b** STEM image, **c** elemental mapping, and **d** XRD pattern of Ru_1/FeO_x. Scale bar, 10 nm

Supplementary Table 26. Ru content and BET surface area for Ru_1/FeO_x

Sample	Ru (wt%) [a]	BET surface area (m^2 g^{-1}) [b]
Ru_1/FeO_x	0.23	38.7

[a] ICP-OES. [b] Sorption isotherm of N_2 at 77 K.

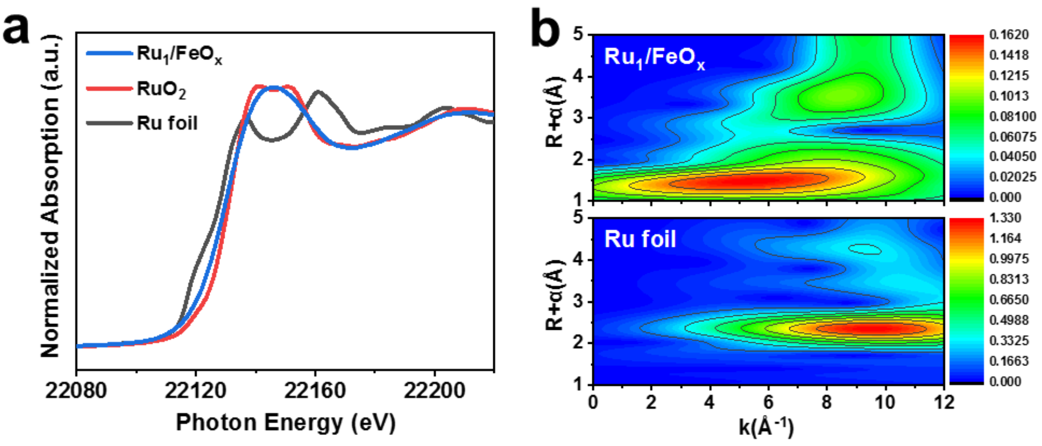

Supplementary Fig. 40 | Structural characterization results of Ru_1/FeO_x. a XANES Ru K-edge for Ru_1/FeO_x, RuO_2, and Ru foil. **b** WT of Ru K-edge EXAFS of Ru_1/FeO_x and Ru foil.

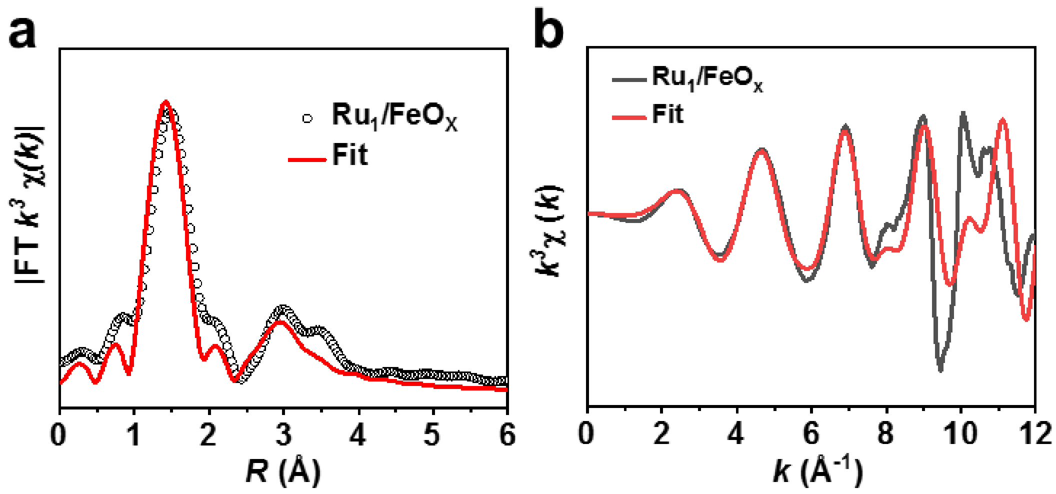

Supplementary Fig. 41 | EXAFS fitting for the Ru_1/FeO_x. **a** FT k^3-weighted $\chi(k)$-function of the EXAFS spectra for Ru K-edge and corresponding R-space fitting curves for the Ru_1/FeO_x catalyst. **b** EXAFS k space fitting curve and the experimental one of Ru_1/FeO_x.

Supplementary Table 27. Structural parameter of EXAFS fitting for the Ru_1/FeO_x

Sample	Scattering pair	CN [a]	R (Å) [b]	σ^2 (× 10^{-3} Å^2) [c]	ΔE_0 (eV) [d]	R factor [e]
Ru_1/FeO_x	Ru–O	4.3±0.5	1.97±0.01	1.0±2.1	-1.4±1.5	0.02
	Ru-O-Fe	0.8±1.2	3.33±0.03	3.8±12.3		

[a] CN is the coordination number; [b] R is interatomic distance (the bond length between central atoms and surrounding coordination atoms); [c] σ^2 is Debye-Waller factor (a measure of thermal and static disorder in absorber-scatterer distances); [d] ΔE_0 is edge-energy shift (the difference between the zero kinetic energy value of the sample and that of the theoretical model). [e] R factor is used to value the goodness of the fitting. Data range: $2.8 \le k \le 9.2$ Å^{-1}, $1.0 \le R \le 4.1$ Å.

Ru_1/ZnO

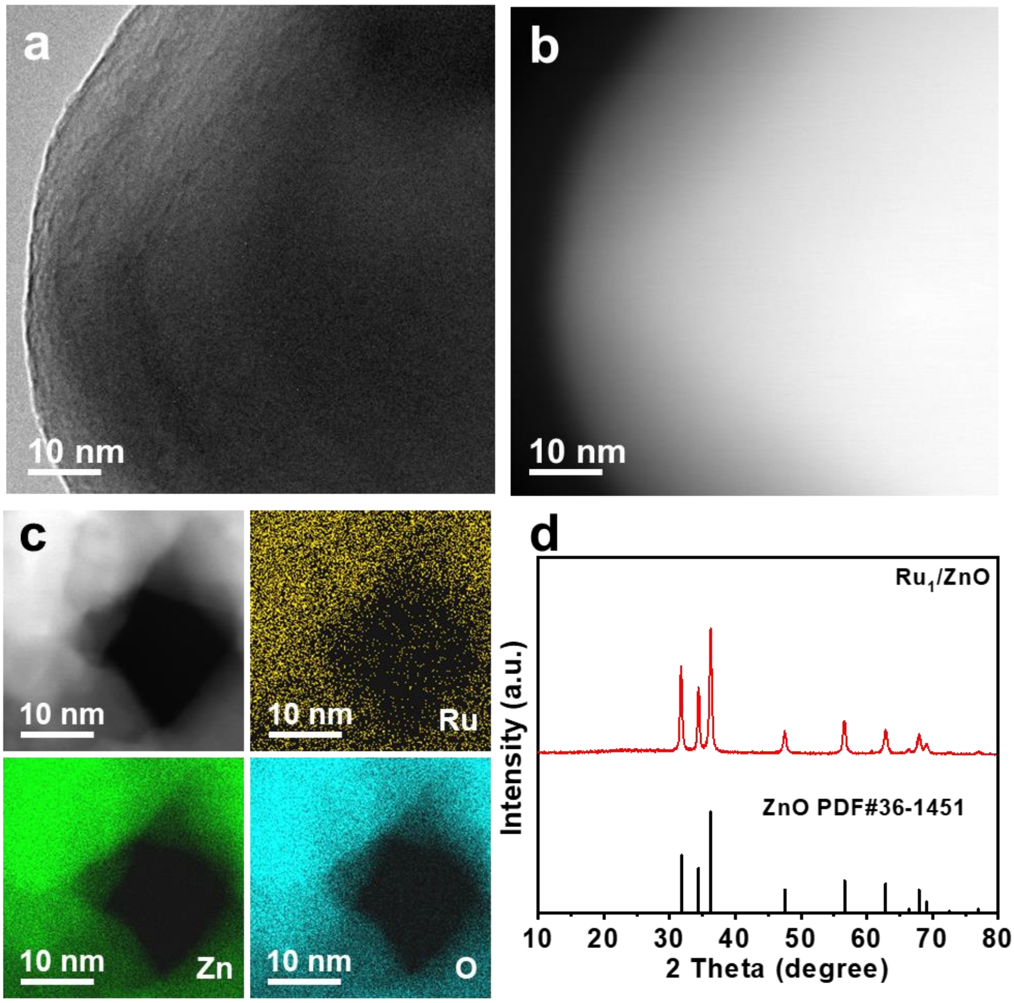

Supplementary Fig. 42 | Structural characterization results of Ru_1/ZnO. **a** TEM image, **b** STEM image, **c** elemental mapping, and **d** XRD pattern of Ru_1/ZnO. Scale bar, 10 nm

Supplementary Table 28. Ru content and BET surface area for Ru_1/ZnO

Sample	Ru (wt%) [a]	BET surface area ($m^2\ g^{-1}$) [b]
Ru_1/ZnO	0.2	24.8

[a] ICP-OES. [b] Sorption isotherm of N_2 at 77 K

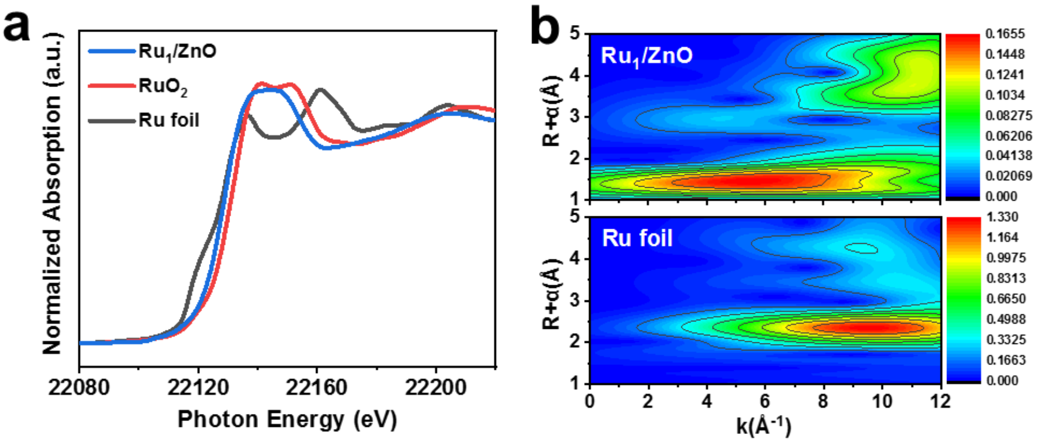

Supplementary Fig. 43 | Structural characterization results of Ru_1/ZnO. a XANES Ru K-edge for Ru_1/ZnO, RuO_2, and Ru foil. **b** WT of Ru K-edge EXAFS of Ru_1/ZnO and Ru foil.

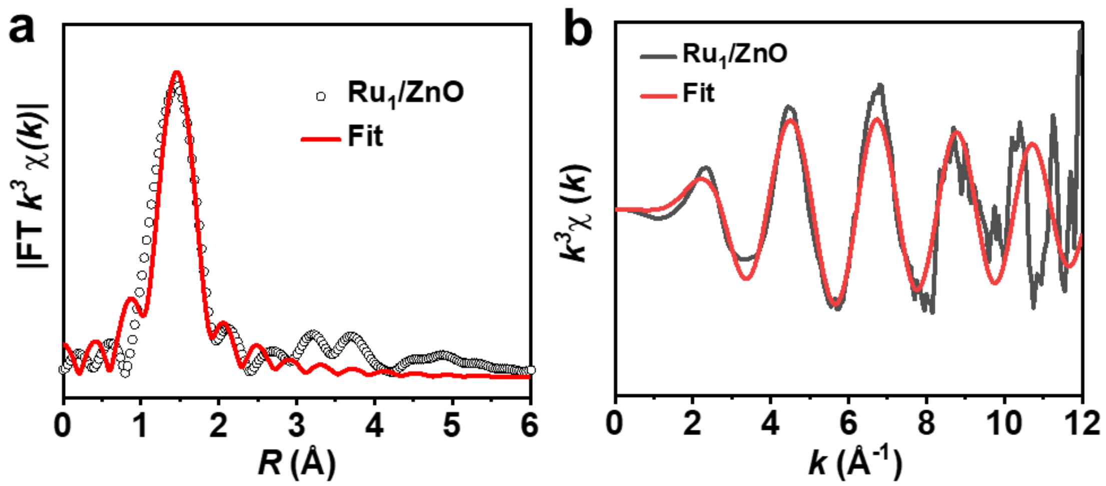

Supplementary Fig. 44 | EXAFS fitting for the Ru_1/ZnO. **a** FT k^3-weighted $\chi(k)$-function of the EXAFS spectra for Ru K-edge and corresponding R-space fitting curves for the Ru_1/ZnO catalyst. **b** EXAFS *k* space fitting curve and the experimental one of Ru_1/ZnO.

Supplementary Table 29. Structural parameter of EXAFS fitting for the Ru_1/ZnO

Sample	Scattering pair	CN [a]	R (Å) [b]	σ^2 (× 10^{-3} Å^2) [c]	ΔE_0 (eV) [d]	R factor [e]
Ru_1/ZnO	Ru-O	4.1±0.7	1.99±0.01	2.7±2.1	-4.0±2.0	0.02

[a] CN is the coordination number; [b] R is interatomic distance (the bond length between central atoms and surrounding coordination atoms); [c] σ^2 is Debye-Waller factor (a measure of thermal and static disorder in absorber-scatterer distances); [d] ΔE_0 is edge-energy shift (the difference between the zero kinetic energy value of the sample and that of the theoretical model). [e] R factor is used to value the goodness of the fitting. Data range: $2.7 \leq k \leq 10.4$ Å^{-1}, $1.1 \leq R \leq 2.6$ Å.

Mn_1/N-C

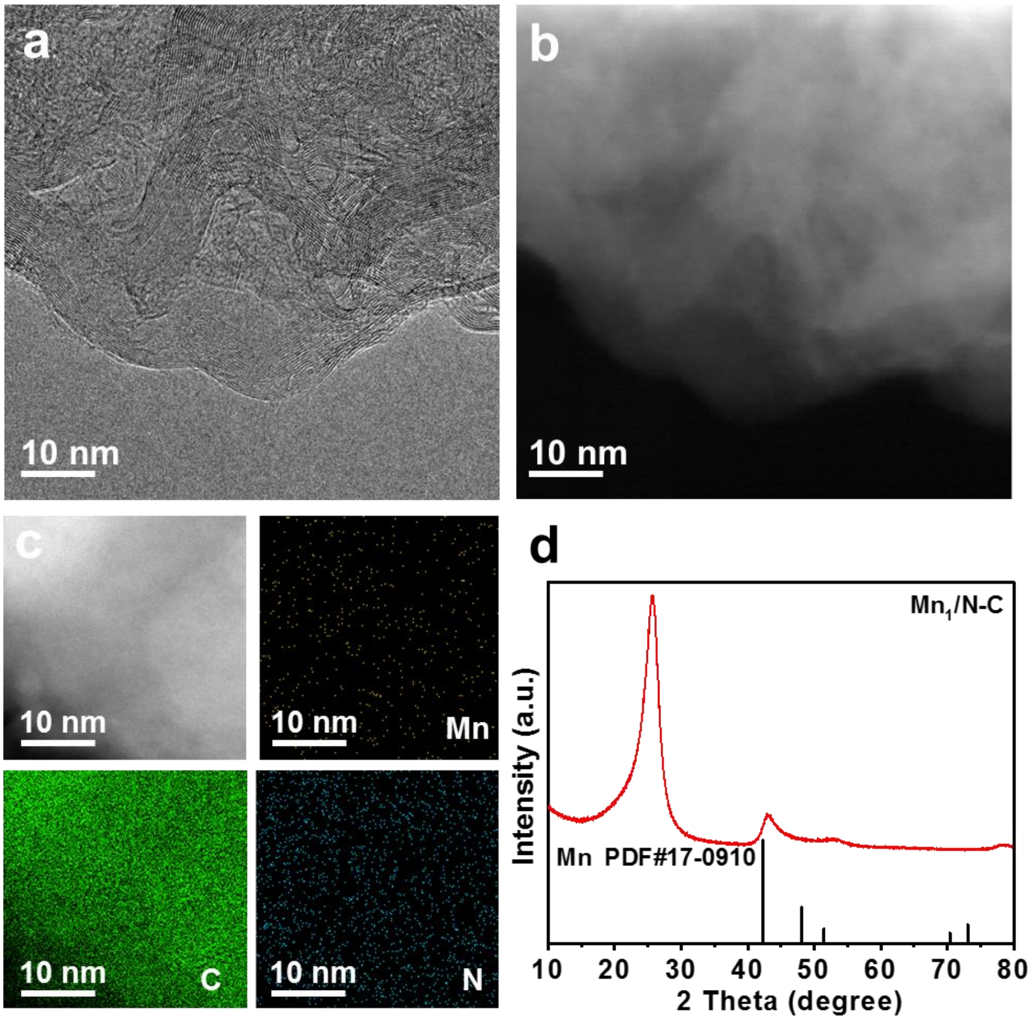

Supplementary Fig. 45 | Structural characterization results of Mn_1/N-C. a TEM image, **b** STEM image, **c** elemental mapping, and **d** XRD pattern of Mn_1/N-C. Scale bar, 10 nm.

Supplementary Table 30. Mn content, N content and BET surface area for Mn_1/N-C

Sample	Mn (wt%) [a]	N (wt%) [b]	BET surface area (m^2 g^{-1}) [c]
Mn_1/N-C	0.13	0.83	88.9

[a] ICP-OES. [b] EA. [c] Sorption isotherm of N_2 at 77 K.

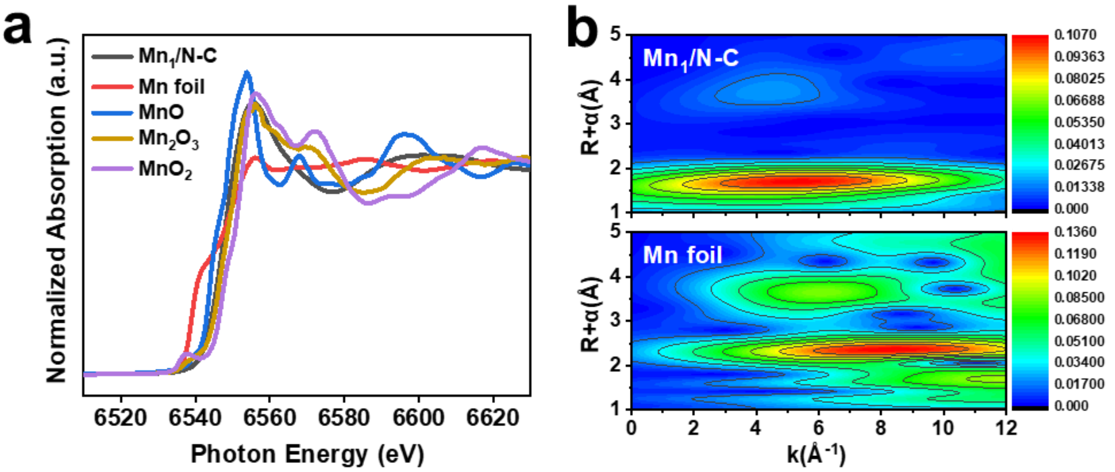

Supplementary Fig. 46 | Structural characterization results of Mn_1/N-C. a XANES Mn K-edge for Mn_1/N-C, MnO, Mn_2O_3, MnO_2, and Mn foil. **b** WT of Mn K-edge EXAFS of Mn_1/N-C and Mn foil.

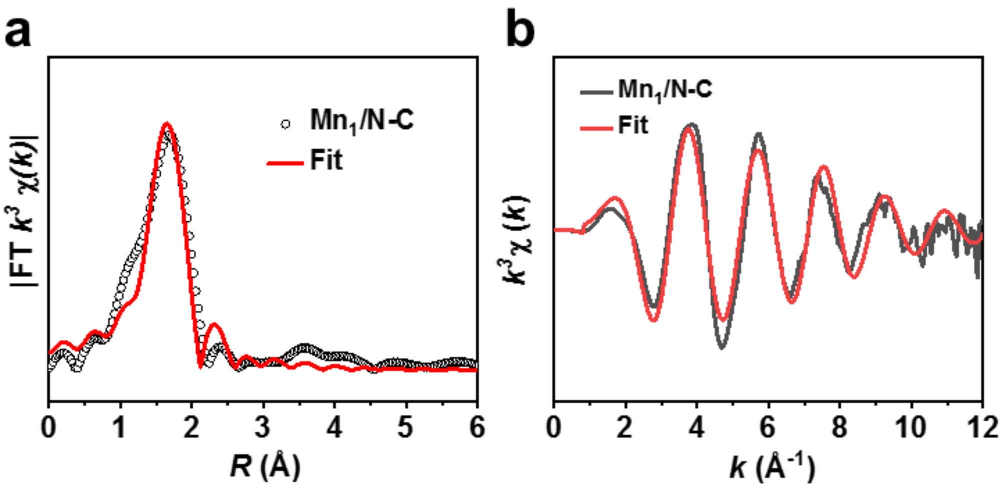

Supplementary Fig. 47 | EXAFS fitting for the Mn_1/N-C. **a** FT k^3-weighted $\chi(k)$-function of the EXAFS spectra for Mn K-edge and corresponding R-space fitting curves for the Mn_1/N-C catalyst. **b** EXAFS *k* space fitting curve and the experimental one of Mn_1/N-C.

Supplementary Table 31. Structural parameter of EXAFS fitting for the Mn_1/N-C

Sample	Scattering pair	CN [a]	R (Å) [b]	σ^2 (× 10^{-3} Å^2) [c]	ΔE_0 (eV) [d]	R factor [e]
Mn_1/N-C	Mn-N	4.1±1.2	2.19±0.02	9.6±5.1	2.7±2.5	0.02

[a] CN is the coordination number; [b] R is interatomic distance (the bond length between central atoms and surrounding coordination atoms); [c] σ^2 is Debye-Waller factor (a measure of thermal and static disorder in absorber-scatterer distances); [d] ΔE_0 is edge-energy shift (the difference between the zero kinetic energy value of the sample and that of the theoretical model). [e] R factor is used to value the goodness of the fitting. Data range: $2.1 \leq k \leq 9.7$ Å^{-1}, $1.0 \leq R \leq 2.2$ Å.

Fe_1/N-C

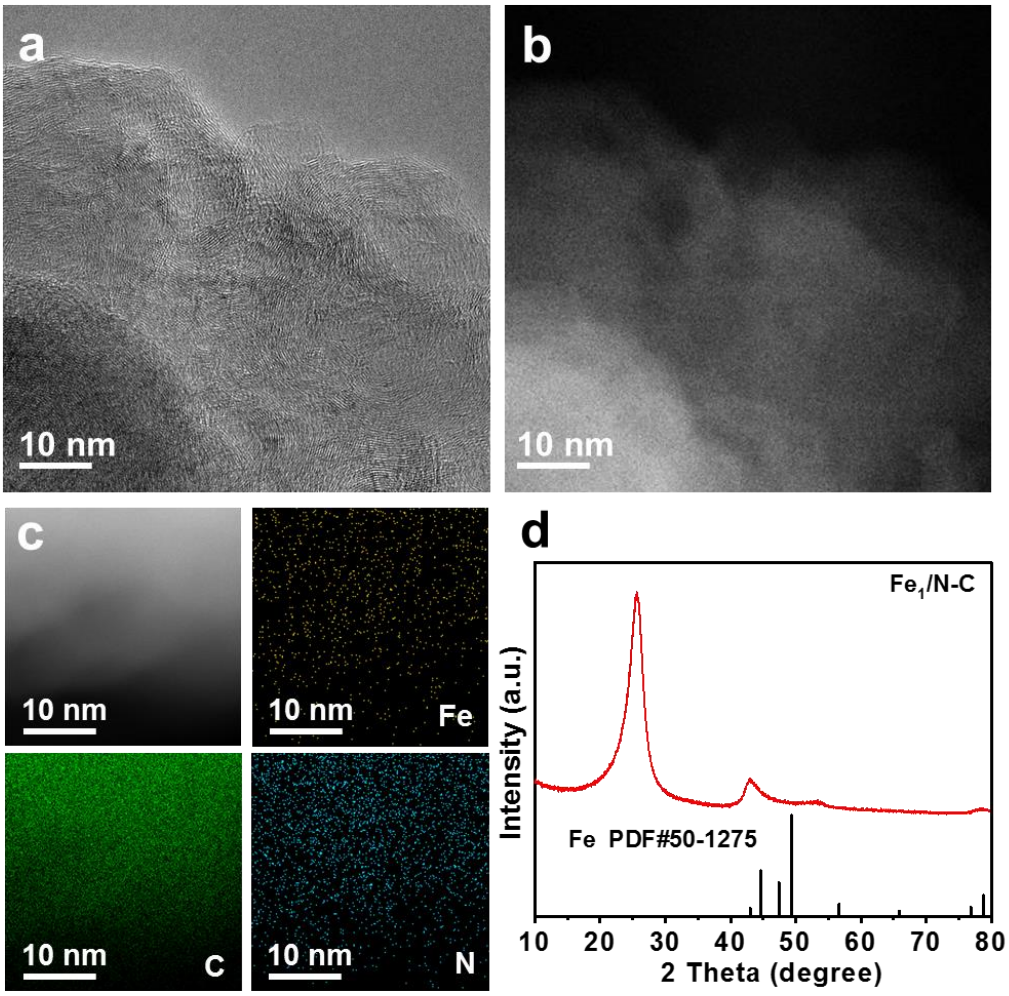

Supplementary Fig. 48 | Structural characterization results of Fe_1/N-C. **a** TEM image, **b** STEM image, **c** elemental mapping, and **d** XRD pattern of Fe_1/N-C. Scale bar, 10 nm.

Supplementary Table 32. Fe content, N content and BET surface area for Fe_1/N-C

Sample	Fe (wt%) [a]	N (wt%) [b]	BET surface area (m^2 g^{-1}) [c]
Fe_1/N-C	0.68	1.67	86.0

[a] ICP-OES. [b] EA. [c] Sorption isotherm of N_2 at 77 K.

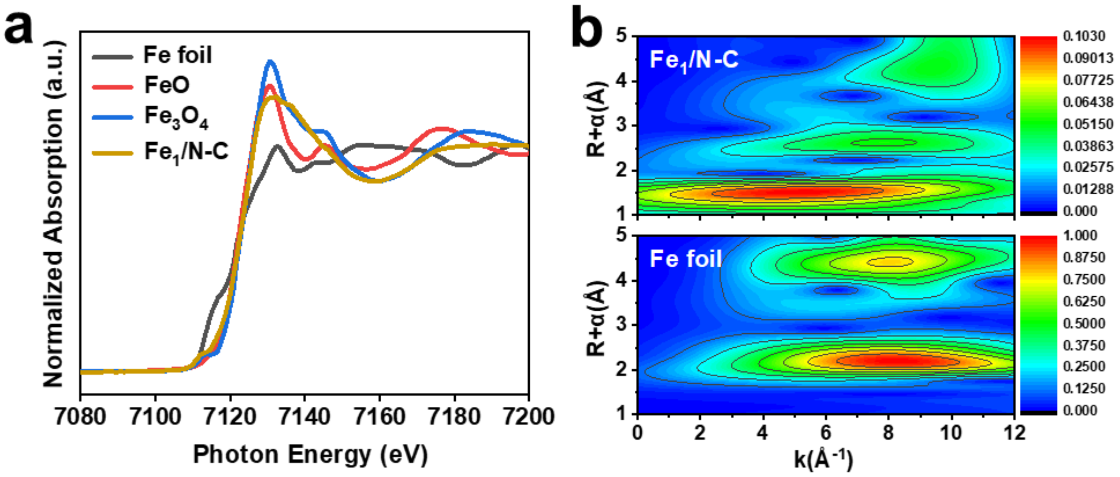

Supplementary Fig. 49 | Structural characterization results of Fe_1/N-C. **a** XANES Fe K-edge for Fe_1/N-C, FeO, Fe_3O_4, and Fe foil. **b** WT of Fe K-edge EXAFS of Fe_1/N-C and Fe foil.

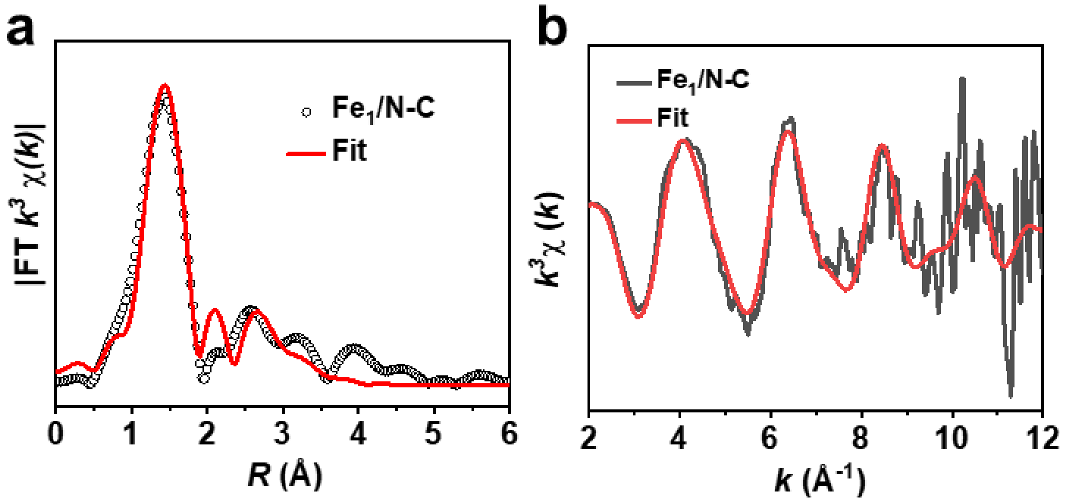

Supplementary Fig. 50 | EXAFS fitting for the Fe_1/N-C. **a** FT k^3-weighted $\chi(k)$-function of the EXAFS spectra for Fe K-edge and corresponding R-space fitting curves for the Fe_1/N-C catalyst. **b** EXAFS k space fitting curve and the experimental one of Fe_1/N-C.

Supplementary Table 33. Structural parameter of EXAFS fitting for the Fe_1/N-C

Sample	Scattering pair	CN [a]	R (Å) [b]	σ^2 (× 10^{-3} Å^2) [c]	ΔE_0 (eV) [d]	R factor [e]
Fe_1/N-C	Fe-N	4.2±0.7	1.97±0.02	6.8±3.0	-5.5±1.7	0.009
	Fe-N-C	3.7±2.2	3.41±0.04	2.7±10.1		

[a] CN is the coordination number; [b] R is interatomic distance (the bond length between central atoms and surrounding coordination atoms); [c] σ^2 is Debye-Waller factor (a measure of thermal and static disorder in absorber-scatterer distances); [d] ΔE_0 is edge-energy shift (the difference between the zero kinetic energy value of the sample and that of the theoretical model). [e] R factor is used to value the goodness of the fitting. Data range: $2.5 \le k \le 8.9$ Å^{-1}, $1.0 \le R \le 3.6$ Å.

Co_1/N-C

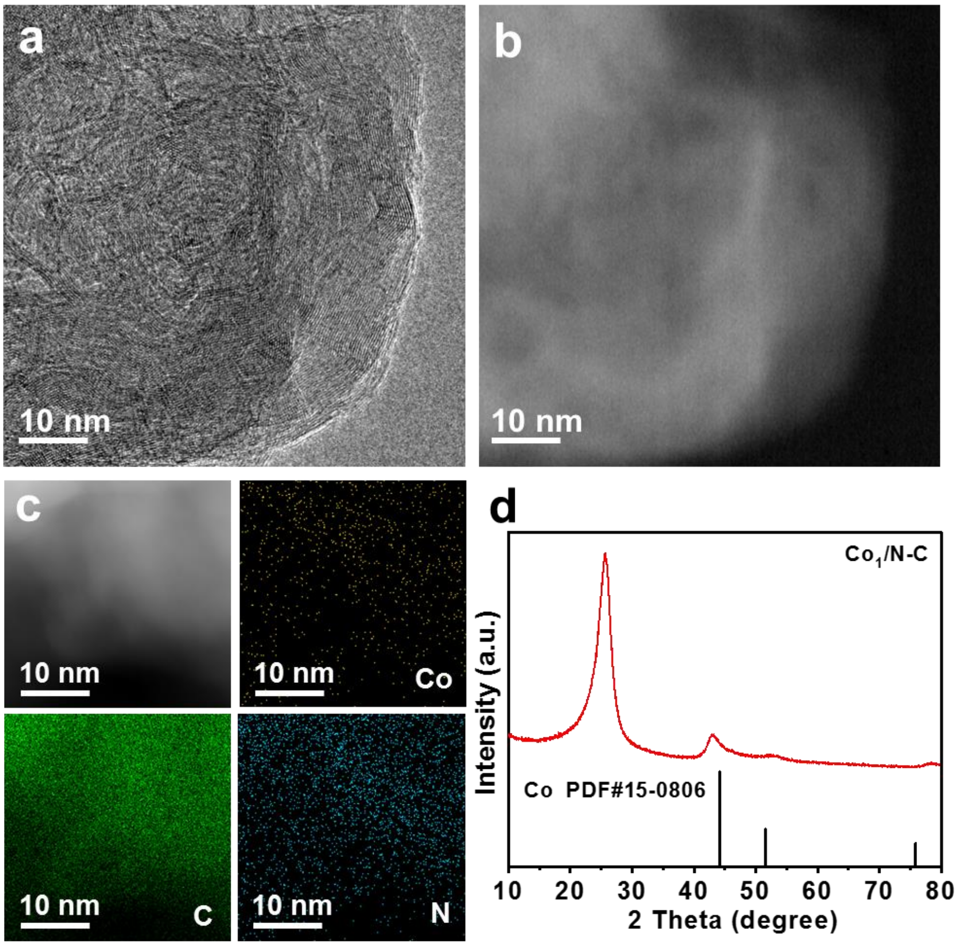

Supplementary Fig. 51 | Structural characterization results of Co_1/N-C. **a** TEM image, **b** STEM image, **c** elemental mapping, and **d** XRD pattern of Co_1/N-C. Scale bar, 10 nm.

Supplementary Table 34. Co content, N content and BET surface area for Co_1/N-C

Sample	Co (wt%) [a]	N (wt%) [b]	BET surface area (m^2 g^{-1}) [c]
Co_1/N-C	0.22	1.08	95.7

[a] ICP-OES. [b] EA. [c] Sorption isotherm of N_2 at 77 K.

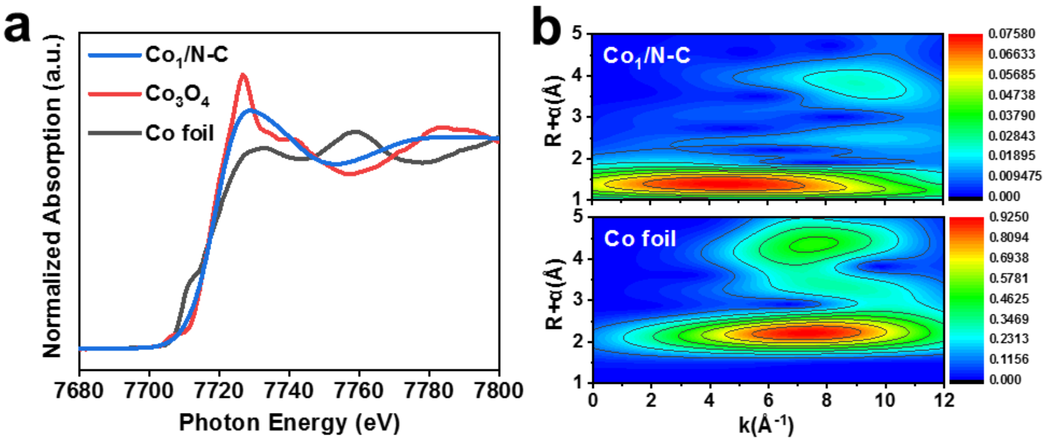

Supplementary Fig. 52 | Structural characterization results of Co_1/N-C. a XANES Co K-edge for Co_1/N-C, Co_3O_4, and Co foil. **b** WT of Co K-edge EXAFS of Co_1/N-C and Co foil.

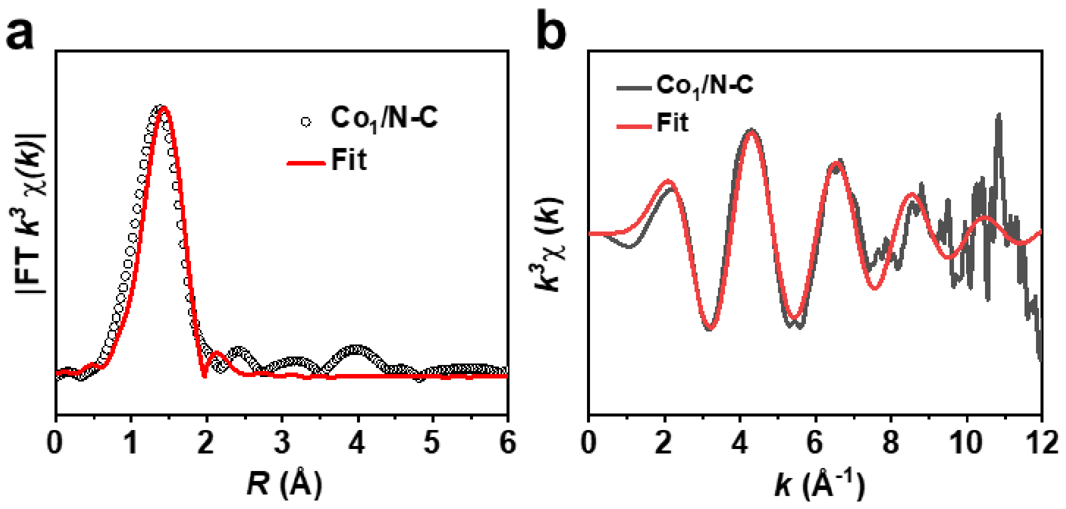

Supplementary Fig. 53 | EXAFS fitting for the Co_1/N-C. **a** FT k^3-weighted $\chi(k)$-function of the EXAFS spectra for Co K-edge and corresponding R-space fitting curves for the Co_1/N-C catalyst. **b** EXAFS *k* space fitting curve and the experimental one of Co_1/N-C.

Supplementary Table 35. Structural parameter of EXAFS fitting for the Co_1/N-C

Sample	Scattering pair	CN [a]	R (Å) [b]	σ^2 ($\times 10^{-3}$ $Å^2$) [c]	ΔE_0 (eV) [d]	R factor [e]
Co_1/N-C	Co-N	4.1±1.1	1.96±0.03	11.7±4.9	-1.2±2.8	0.02

[a] CN is the coordination number; [b] R is interatomic distance (the bond length between central atoms and surrounding coordination atoms); [c] σ^2 is Debye-Waller factor (a measure of thermal and static disorder in absorber-scatterer distances); [d] ΔE_0 is edge-energy shift (the difference between the zero kinetic energy value of the sample and that of the theoretical model). [e] R factor is used to value the goodness of the fitting. Data range: $2.6 \leq k \leq 9.0$ $Å^{-1}$, $1.0 \leq R \leq 2.5$ Å.

Ni_1/N-C

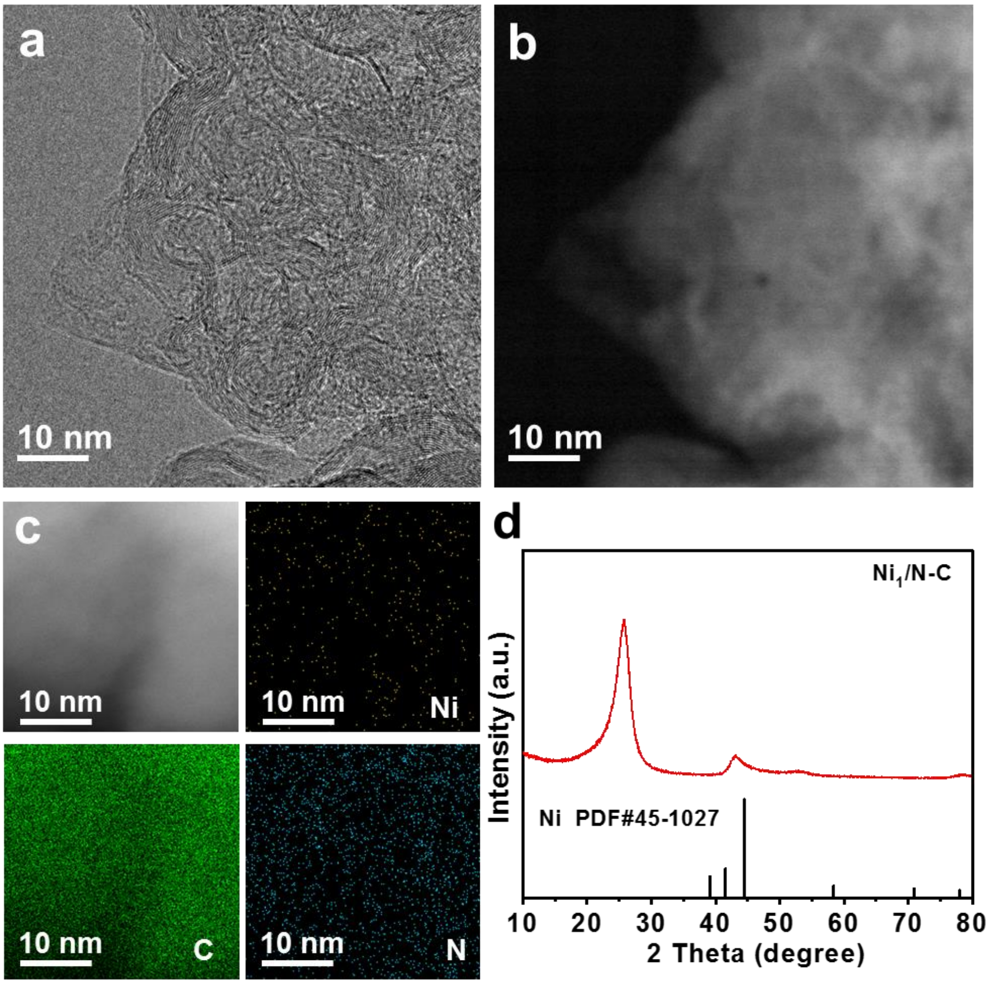

Supplementary Fig. 54 | Structural characterization results of Ni_1/N-C. a TEM image, **b** STEM image, **c** elemental mapping, and **d** XRD pattern of Ni_1/N-C. Scale bar, 10 nm.

Supplementary Table 36. Ni content, N content and BET surface area for Ni_1/N-C

Sample	Ni (wt%) [a]	N (wt%) [b]	BET surface area (m^2 g^{-1}) [c]
Ni_1/N-C	0.16	0.97	94.4

[a] ICP-OES. [b] EA. [c] Sorption isotherm of N_2 at 77 K.

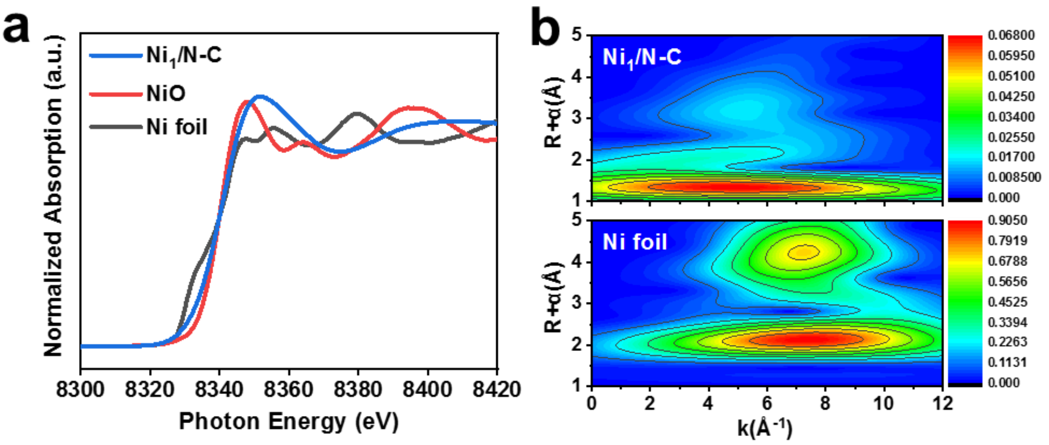

Supplementary Fig. 55 | Structural characterization results of Ni_1/N-C. **a** XANES Ni K-edge for Ni_1/N-C, NiO, and Ni foil. **b** WT of Ni K-edge EXAFS of Ni_1/N-C and Ni foil.

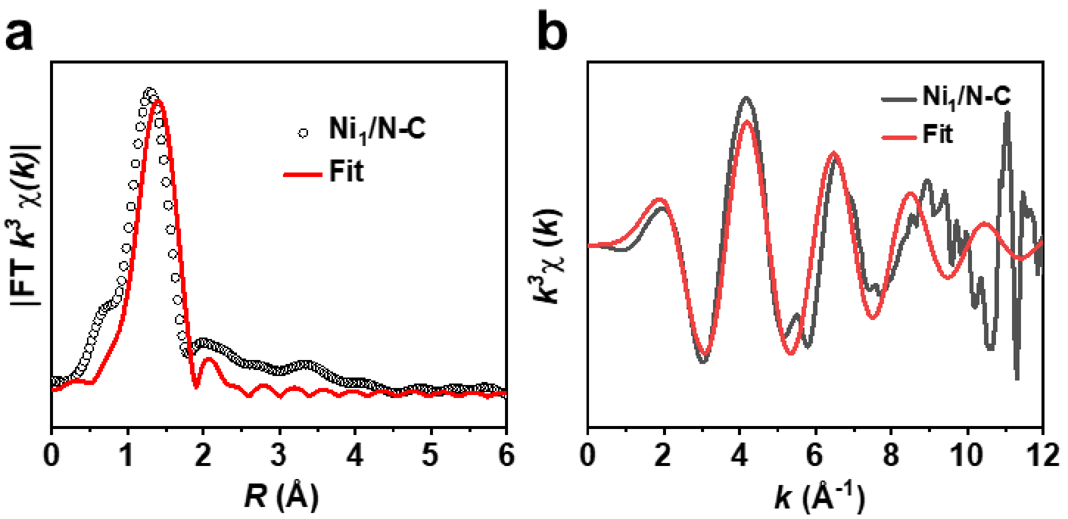

Supplementary Fig. 56 | EXAFS fitting for the Ni$_1$/N-C. **a** FT k^3-weighted $\chi(k)$-function of the EXAFS spectra for Ni K-edge and Ni corresponding R-space fitting curves for the Ni$_1$/N-C catalyst. **b** EXAFS k space fitting curve and the experimental one of Ni$_1$/N-C.

Supplementary Table 37. Structural parameter of EXAFS fitting for the Ni$_1$/N-C

Sample	Scattering pair	CN [a]	R (Å) [b]	σ^2 (× 10^{-3} Å^2) [c]	ΔE_0 (eV) [d]	R factor [e]
Ni$_1$/N-C	Ni-N	4.3±1.6	1.95±0.04	11.8±7.6	-6.4±4.0	0.03

[a] CN is the coordination number; [b] R is interatomic distance (the bond length between central atoms and surrounding coordination atoms); [c] σ^2 is Debye-Waller factor (a measure of thermal and static disorder in absorber-scatterer distances); [d] ΔE_0 is edge-energy shift (the difference between the zero kinetic energy value of the sample and that of the theoretical model). [e] R factor is used to value the goodness of the fitting. Data range: $2.0 \le k \le 9.0$ Å^{-1}, $1.0 \le R \le 2.4$ Å.

Zn_1/N-C

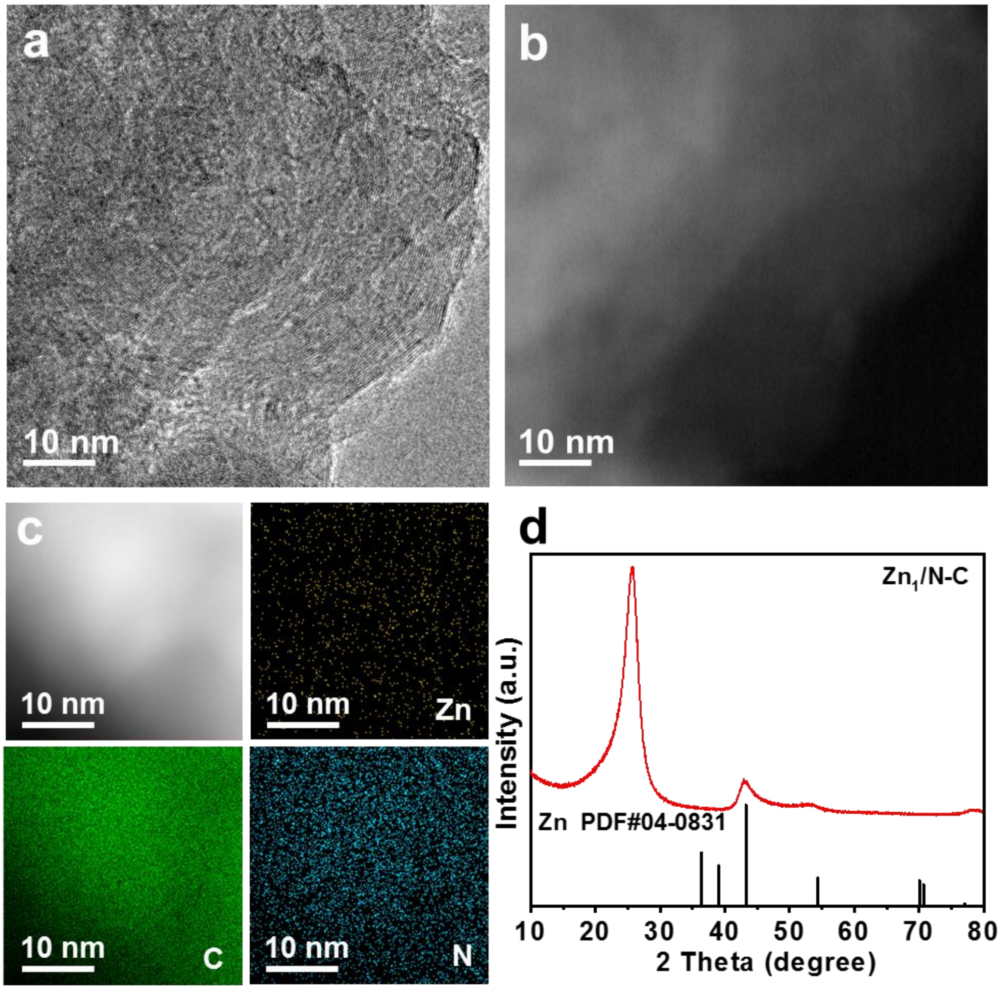

Supplementary Fig. 57 | Structural characterization results of Zn_1/N-C. a TEM image, **b** STEM image, **c** elemental mapping, and **d** XRD pattern of Zn_1/N-C. Scale bar, 10 nm.

Supplementary Table 38. Zn content, N content and BET surface area for Zn_1/N-C

Sample	Zn (wt%) [a]	N (wt%) [b]	BET surface area (m^2 g^{-1}) [c]
Zn_1/N-C	0.36	1.4	77.3

[a] ICP-OES. [b] EA. [c] Sorption isotherm of N_2 at 77 K.

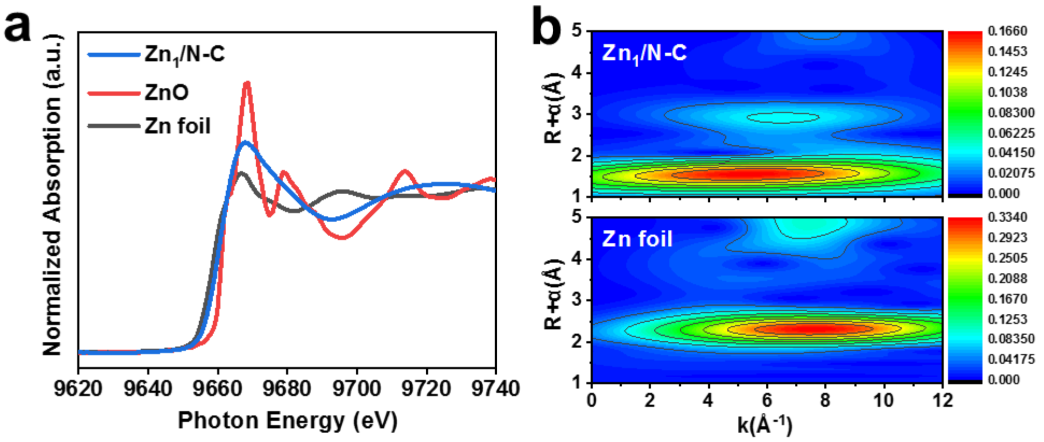

Supplementary Fig. 58 | Structural characterization results of Zn_1/N-C. a XANES Zn K-edge for Zn_1/N-C, ZnO, and Zn foil. **b** WT of Zn K-edge EXAFS of Zn_1/N-C and Zn foil.

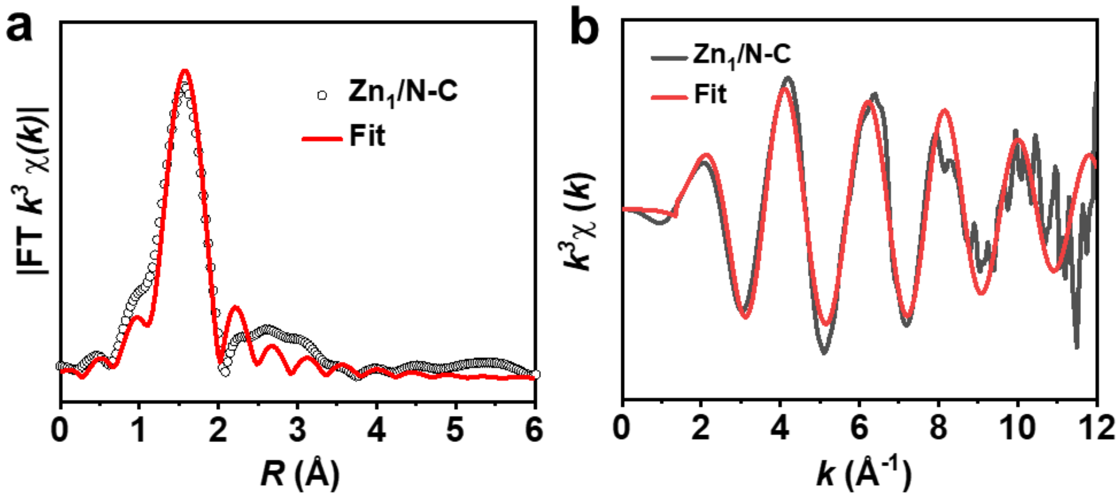

Supplementary Fig. 59 | EXAFS fitting for the Zn_1/N-C. **a** FT k^3-weighted $\chi(k)$-function of the EXAFS spectra for Zn K-edge and Zn corresponding R-space fitting curves for the Zn_1/N-C catalyst. **b** EXAFS *k* space fitting curve and the experimental one of Zn_1/N-C.

Supplementary Table 39. Structural parameter of EXAFS fitting for the Zn_1/N-C

Sample	Scattering pair	CN [a]	R (Å) [b]	σ^2 (× 10^{-3} Å^2) [c]	ΔE_0 (eV) [d]	R factor [e]
Zn_1/N-C	Zn-N	4.3±0.9	2.02±0.02	4.1±3.1	7.4±2.3	0.01

[a] CN is the coordination number; [b] R is interatomic distance (the bond length between central atoms and surrounding coordination atoms); [c] σ^2 is Debye-Waller factor (a measure of thermal and static disorder in absorber-scatterer distances); [d] ΔE_0 is edge-energy shift (the difference between the zero kinetic energy value of the sample and that of the theoretical model). [e] R factor is used to value the goodness of the fitting. Data range: $3.1 \leq k \leq 10.2$ Å^{-1}, $1.3 \leq R \leq 2.8$ Å.

Pd_1/Cu

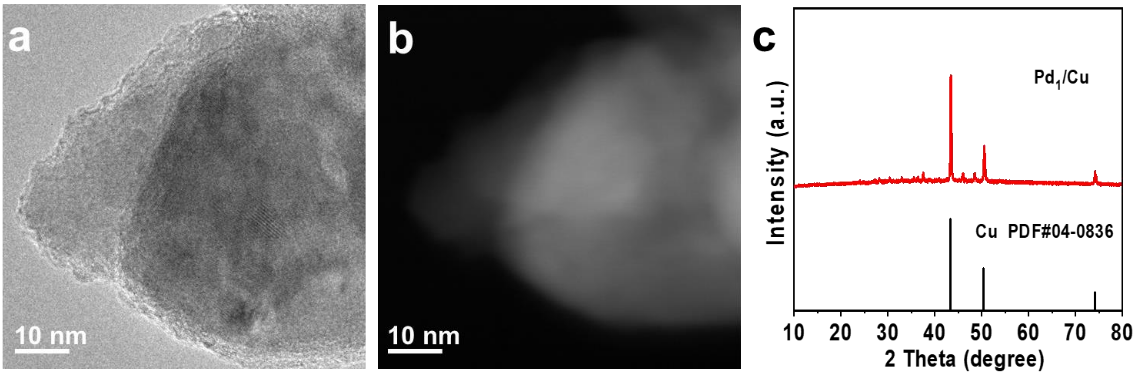

Supplementary Fig. 60 | Structural characterization results of Pd_1/Cu. **a** TEM image, **b** STEM image and **c** XRD pattern of Pd_1/Cu. Scale bar, 10 nm.

Supplementary Table 40. Pd content and BET surface area for Pd_1/Cu

Sample	Pd (wt%) [a]	BET surface area ($m^2\ g^{-1}$) [b]
Pd_1/Cu	0.12	3.19

[a] ICP-OES. [b] Sorption isotherm of N_2 at 77 K.

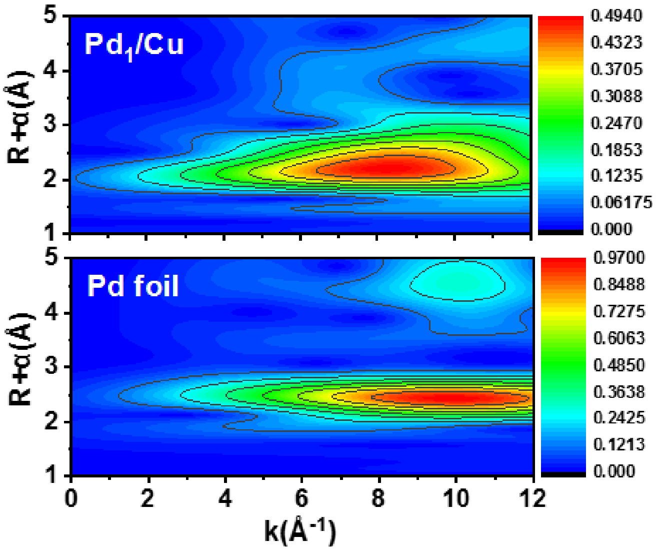

Supplementary Fig. 61 | WT results. WT of Pd K edge EXAFS of Pd_1/Cu and Pd foil.

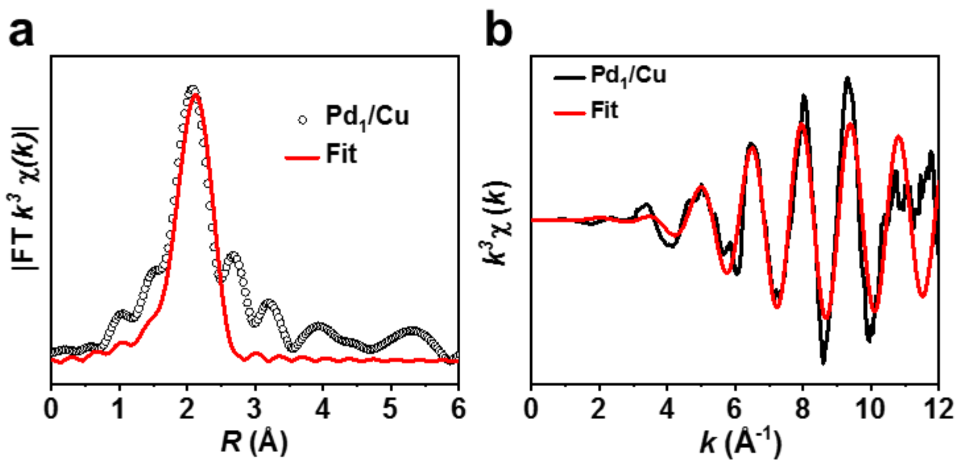

Supplementary Fig. 62 | EXAFS fitting for the Pd_1/Cu. a FT k^3-weighted $\chi(k)$-function of the EXAFS spectra for Pd K-edge and corresponding R-space fitting curves for the Pd_1/Cu catalyst. **b** EXAFS k space fitting curve and the experimental one of Pd_1/Cu.

Supplementary Table 41. Structural parameter of EXAFS fitting for the Pd_1/Cu

Sample	Scattering pair	CN [a]	R (Å) [b]	σ^2 (× 10^{-3} Å^2) [c]	ΔE_0 (eV) [d]	R factor [e]
Pd_1/Cu	Pd-Cu	7.8±3.8	2.55±0.03	3.9±3.9	-3.3±5.4	0.04

[a] CN is the coordination number; [b] R is interatomic distance (the bond length between central atoms and surrounding coordination atoms); [c] σ^2 is Debye-Waller factor (a measure of thermal and static disorder in absorber-scatterer distances); [d] ΔE_0 is edge-energy shift (the difference between the zero kinetic energy value of the sample and that of the theoretical model). [e] R factor is used to value the goodness of the fitting. Data range: $2.1 \leq k \leq 9.0$ Å^{-1}, $1.0 \leq R \leq 2.4$ Å.

Pd_2/FeO_x

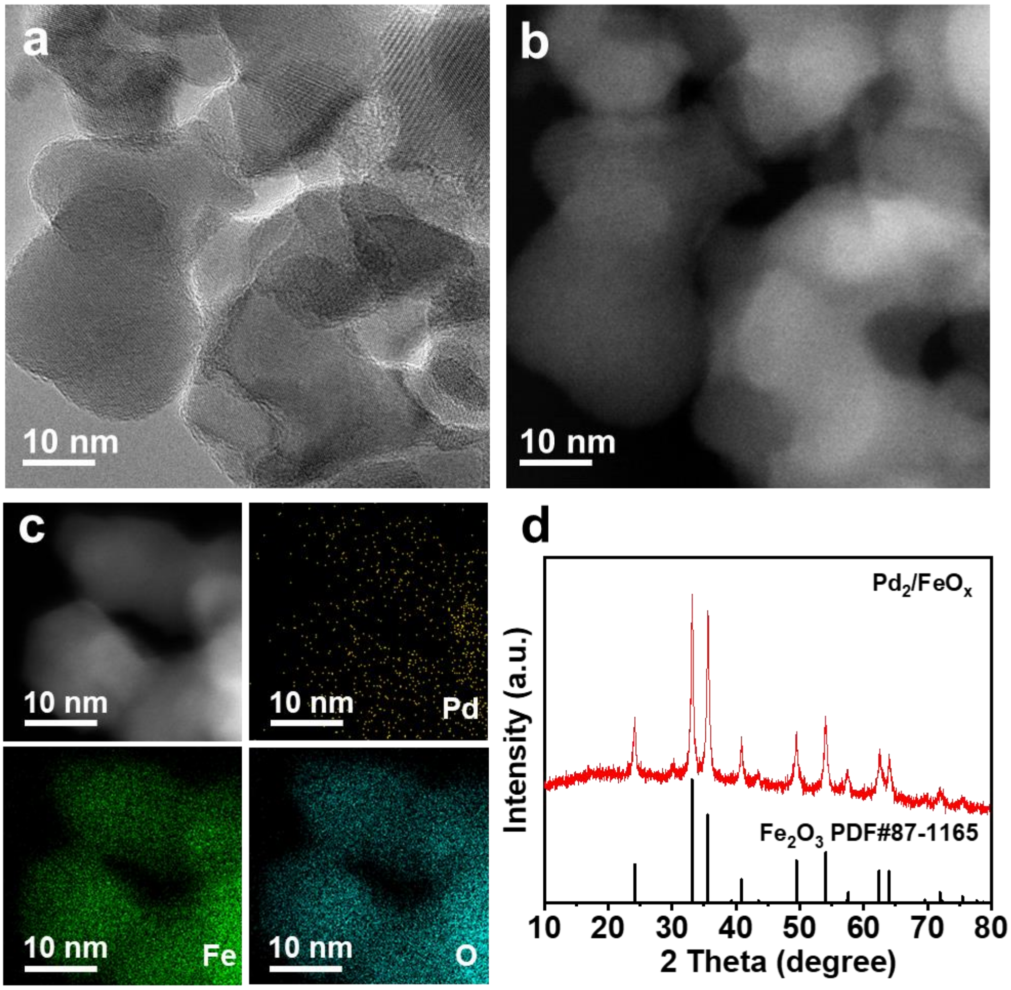

Supplementary Fig. 63 | Structural characterization results of Pd_2/FeO_x. a TEM image, **b** STEM image, **c** elemental mapping, and **d** XRD pattern of Pd_2/FeO_x. Scale bar, 10 nm.

Supplementary Table 42. Pd content and BET surface area for Pd_2/FeO_x

Sample	Pd (wt%) [a]	BET surface area ($m^2\ g^{-1}$) [b]
Pd_2/FeO_x	0.34	40.4

[a] ICP-OES. [b] Sorption isotherm of N_2 at 77 K.

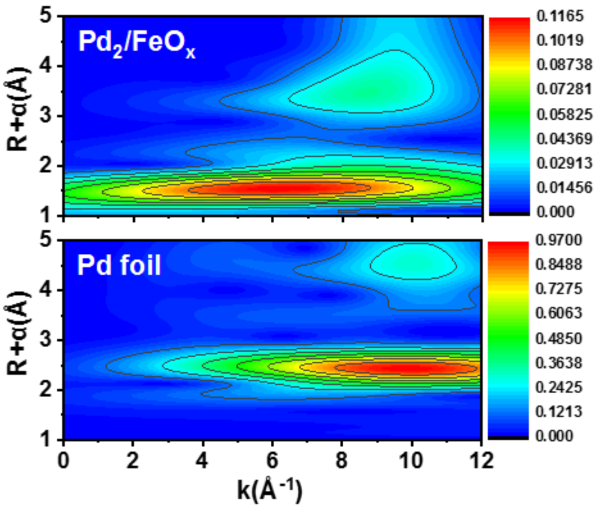

Supplementary Fig. 64 | WT results. WT of Pd K edge EXAFS of Pd_2/FeO_x and Pd foil.

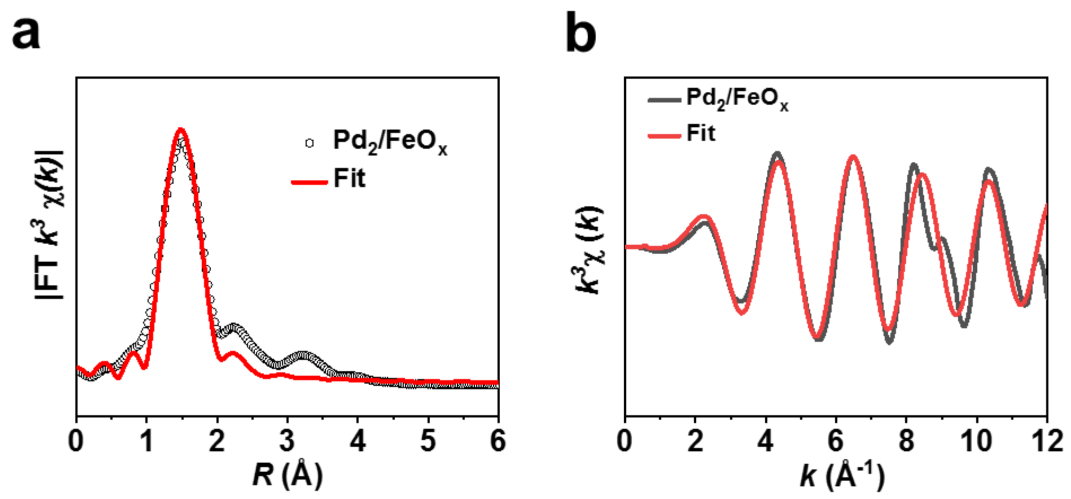

Supplementary Fig. 65 | EXAFS fitting for the Pd_2/FeO_x. **a** Fourier transform (FT) k^3-weighted χ(k)-function of the EXAFS spectra for Pd K-edge and corresponding R-space fitting curves for the Pd_2/FeO_x catalyst. **b** EXAFS k space fitting curve and the experimental one of Pd_2/FeO_x.

Supplementary Table 43. Structural parameter of EXAFS fitting for the Pd_2/FeO_x

Sample	Scattering pair	CN [a]	R (Å) [b]	σ^2 (× 10^{-3} Å^2) [c]	ΔE_0 (eV) [d]	R factor [e]
Pd_2/FeO_x	Pd-O	3.6±1.0	2.04±0.02	2.9±3.2	1.4±3.1	0.02

[a] CN is the coordination number; [b] R is interatomic distance (the bond length between central atoms and surrounding coordination atoms); [c] σ^2 is Debye-Waller factor (a measure of thermal and static disorder in absorber-scatterer distances); [d] ΔE_0 is edge-energy shift (the difference between the zero kinetic energy value of the sample and that of the theoretical model). [e] R factor is used to value the goodness of the fitting. Data range: $2.0 \leq k \leq 8.3$ Å^{-1}, $1.0 \leq R \leq 2.6$ Å.

Pd_1-Pt_1/FeO_x

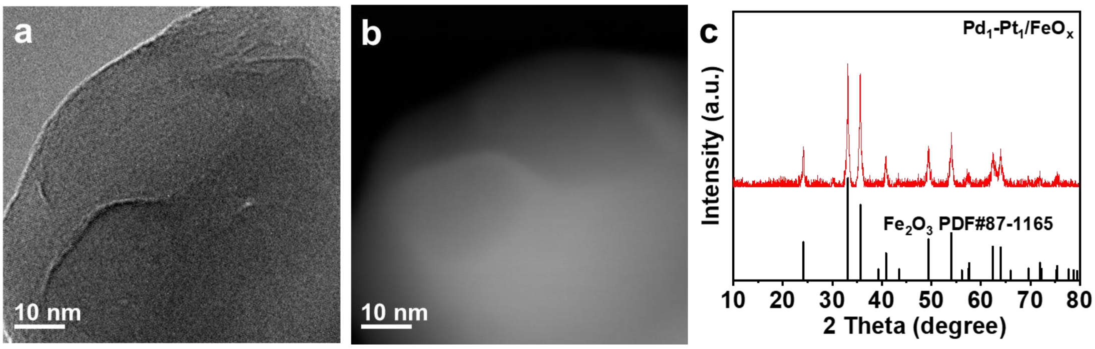

Supplementary Fig. 66 | Structural characterization results of Pd_1-Pt_1/FeO_x. a TEM image, **b** STEM image and **c** XRD pattern of Pd_1-Pt_1/FeO_x. Scale bar, 10 nm.

Supplementary Table 44. Pd content and BET surface area for Pd_1-Pt_1/FeO_x

Sample	Pt (wt%) [a]	Pd (wt%) [a]	BET surface area (m^2 g^{-1}) [b]
Pd_1-Pt_1/FeO_x	0.23	0.28	46.2

[a] ICP-OES. [b] Sorption isotherm of N_2 at 77 K.

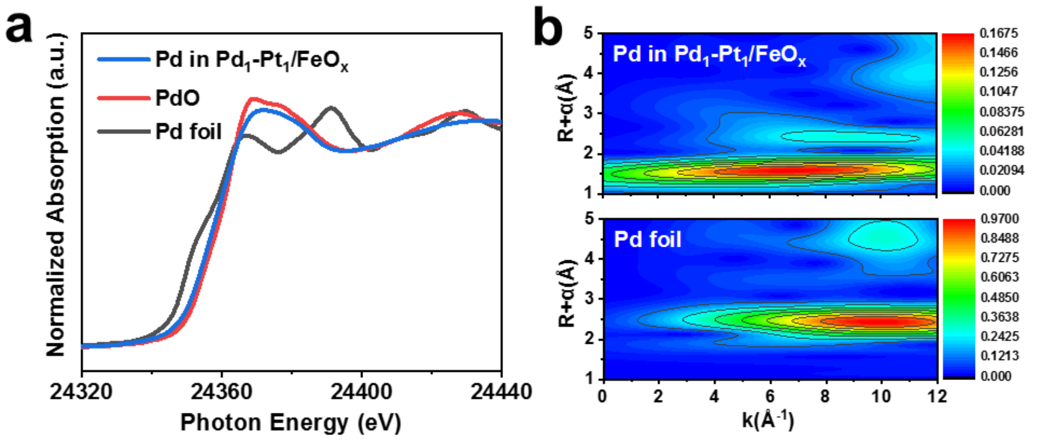

Supplementary Fig. 67 | Structural characterization results of Pd_1-Pt_1/FeO_x. a XANES Pd K-edge for Pd in Pd_1-Pt_1/FeO_x, PdO, and Pd foil. **b** WT of Pd K edge EXAFS of Pd in Pd_1-Pt_1/FeO_x and Pd foil.

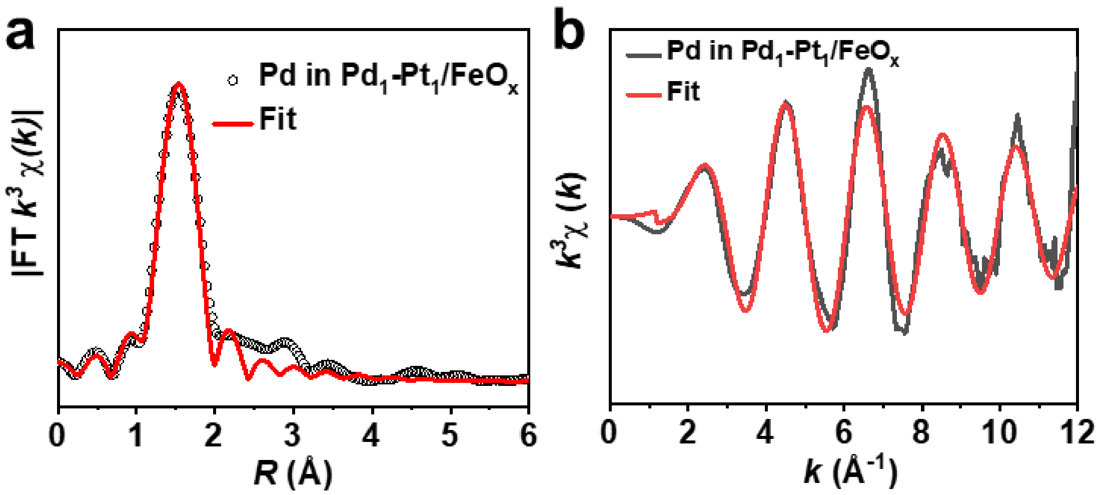

Supplementary Fig. 68 | EXAFS fitting for the Pd_1-Pt_1/FeO_x. a Fourier transform (FT) k^3-weighted $\chi(k)$-function of the EXAFS spectra for Pd K-edge and corresponding R-space fitting curves for the Pd in Pd_1-Pt_1/FeO_x catalyst. **b** EXAFS k space fitting curve and the experimental one of Pd in Pd_1-Pt_1/FeO_x.

Supplementary Table 45. Structural parameter of EXAFS fitting for the Pd in Pd_1-Pt_1/FeO_x

Sample	Scattering pair	CN [a]	R (Å) [b]	σ^2 (× 10^{-3} Å^2) [c]	ΔE_0 (eV) [d]	R factor [e]
Pd in Pd_1-Pt_1/FeO_x	Pd-O	4.0±1.1	2.04±0.02	3.7±2.7	4.9±3.1	0.03

[a] CN is the coordination number; [b] R is interatomic distance (the bond length between central atoms and surrounding coordination atoms); [c] σ^2 is Debye-Waller factor (a measure of thermal and static disorder in absorber-scatterer distances); [d] ΔE_0 is edge-energy shift (the difference between the zero kinetic energy value of the sample and that of the theoretical model). [e] R factor is used to value the goodness of the fitting. Data range: $2.3 \leq k \leq 9.9$ Å^{-1}, $1.0 \leq R \leq 2.4$ Å.

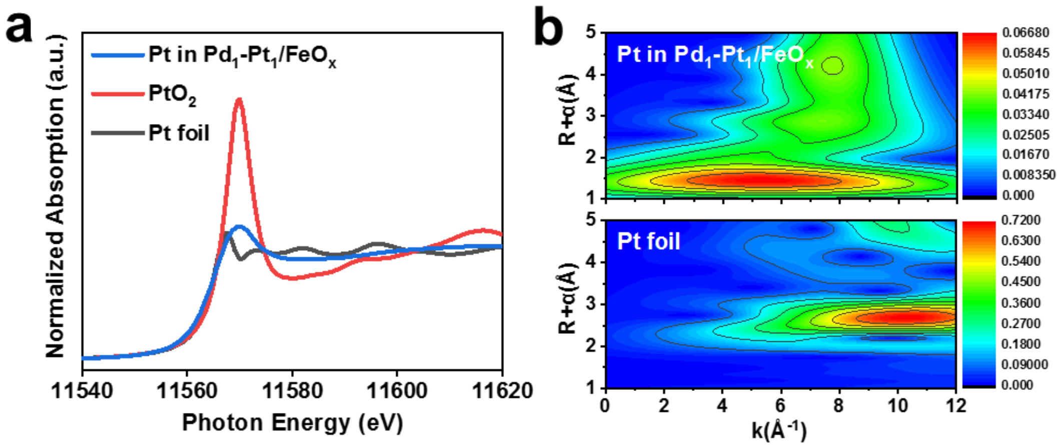

Supplementary Fig. 69 | Structural characterization results of Pd_1-Pt_1/FeO_x. a XANES Pt L_3-edge for Pt in Pd_1-Pt_1/FeO_x, PtO_2, and Pt foil. **b** WT of Pt L_3-edge EXAFS of Pt in Pd_1-Pt_1/FeO_x and Pt foil.

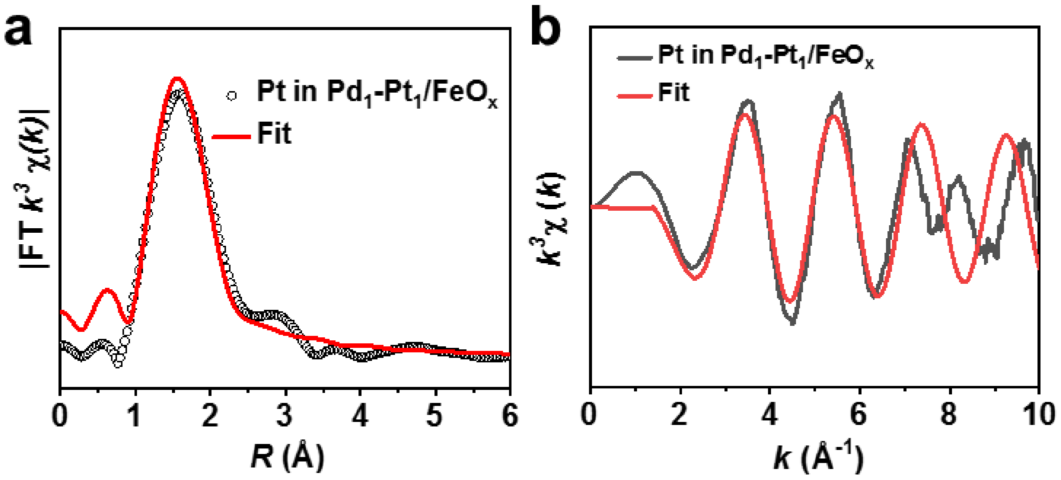

Supplementary Fig. 70 | EXAFS fitting for the Pd_1-Pt_1/FeO_x. **a** Fourier transform (FT) k^3-weighted χ(k)-function of the EXAFS spectra for Pt L_3-edge and corresponding R-space fitting curves for the Pt in Pd_1-Pt_1/FeO_x catalyst. **b** EXAFS k space fitting curve and the experimental one of Pt in Pd_1-Pt_1/FeO_x.

Supplementary Table 46. Structural parameter of EXAFS fitting for the Pt in Pd_1-Pt_1/FeO_x

Sample	Scattering pair	CN [a]	R (Å) [b]	σ^2 (× 10^{-3} $Å^2$) [c]	ΔE_0 (eV) [d]	R factor [e]
Pt in Pd_1-Pt_1/FeO_x	Pt-O	3.8±0.6	2.04±0.02	6.0±3.3	9.1±1.2	0.006

[a] CN is the coordination number; [b] R is interatomic distance (the bond length between central atoms and surrounding coordination atoms); [c] σ^2 is Debye-Waller factor (a measure of thermal and static disorder in absorber-scatterer distances); [d] ΔE_0 is edge-energy shift (the difference between the zero kinetic energy value of the sample and that of the theoretical model). [e] R factor is used to value the goodness of the fitting. Data range: $2.0 \le k \le 8.0$ $Å^{-1}$, $1.0 \le R \le 3.0$ Å.

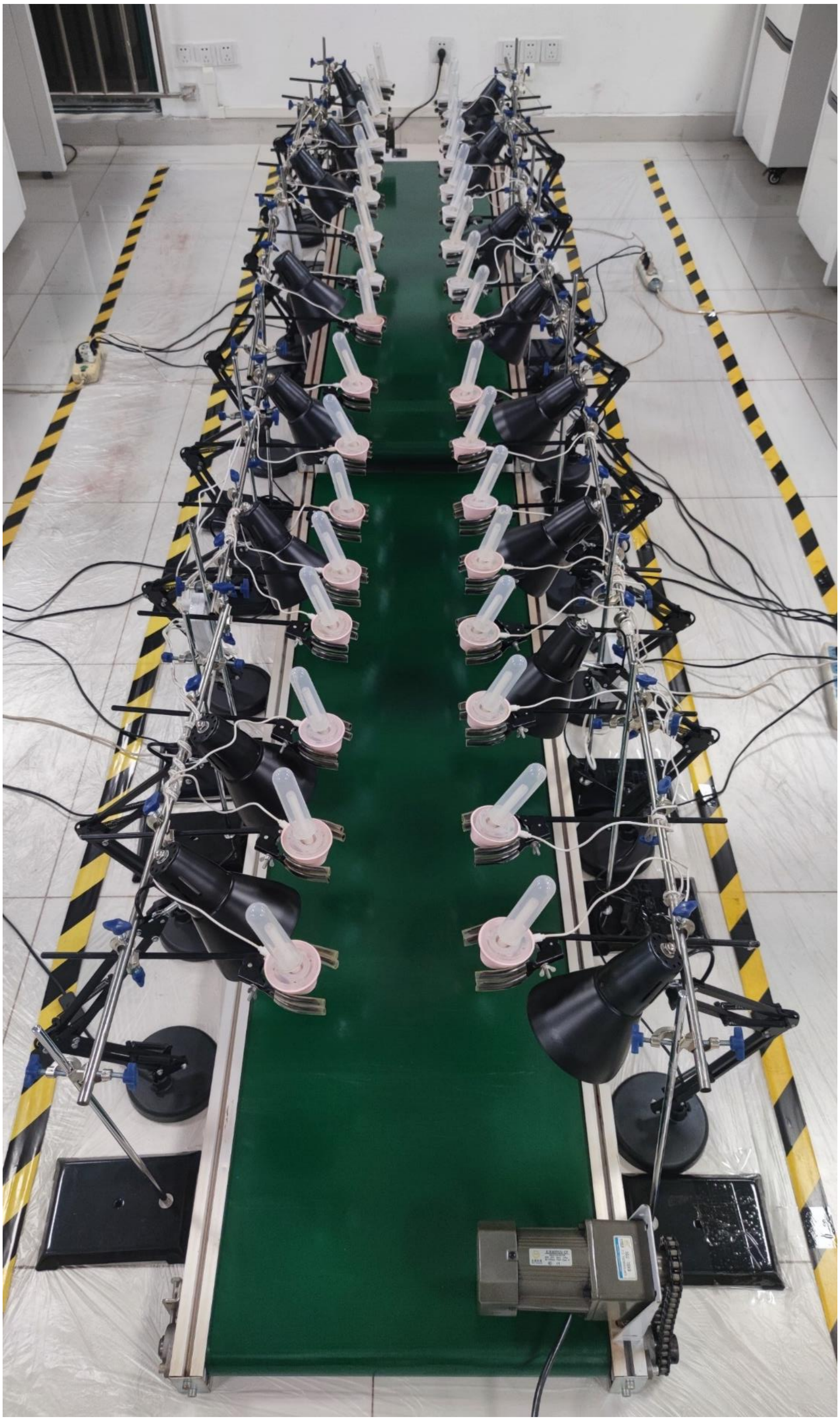

Supplementary Fig. 71 | Production line of SACs. The picture of the homemade production line for the large-scale synthesis of SACs.

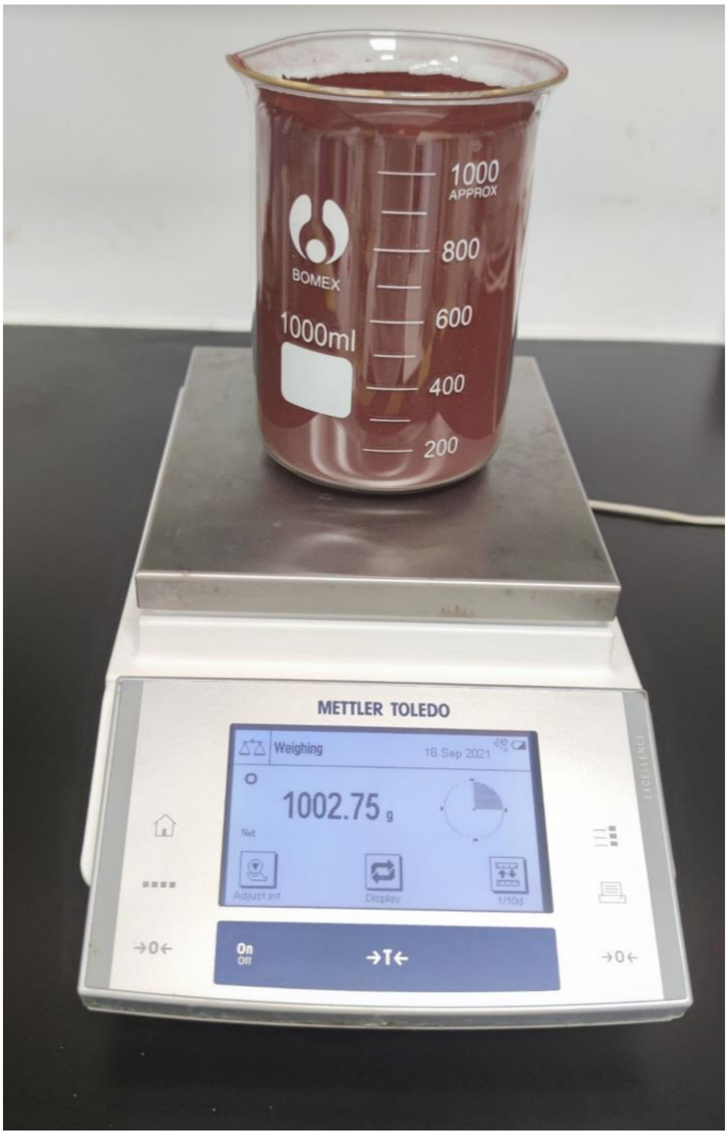

Supplementary Fig. 72 | Kilogram-scale SACs. The picture of Pd_1/FeO_x prepared by the production line.

Pd_1/FeO_x-1

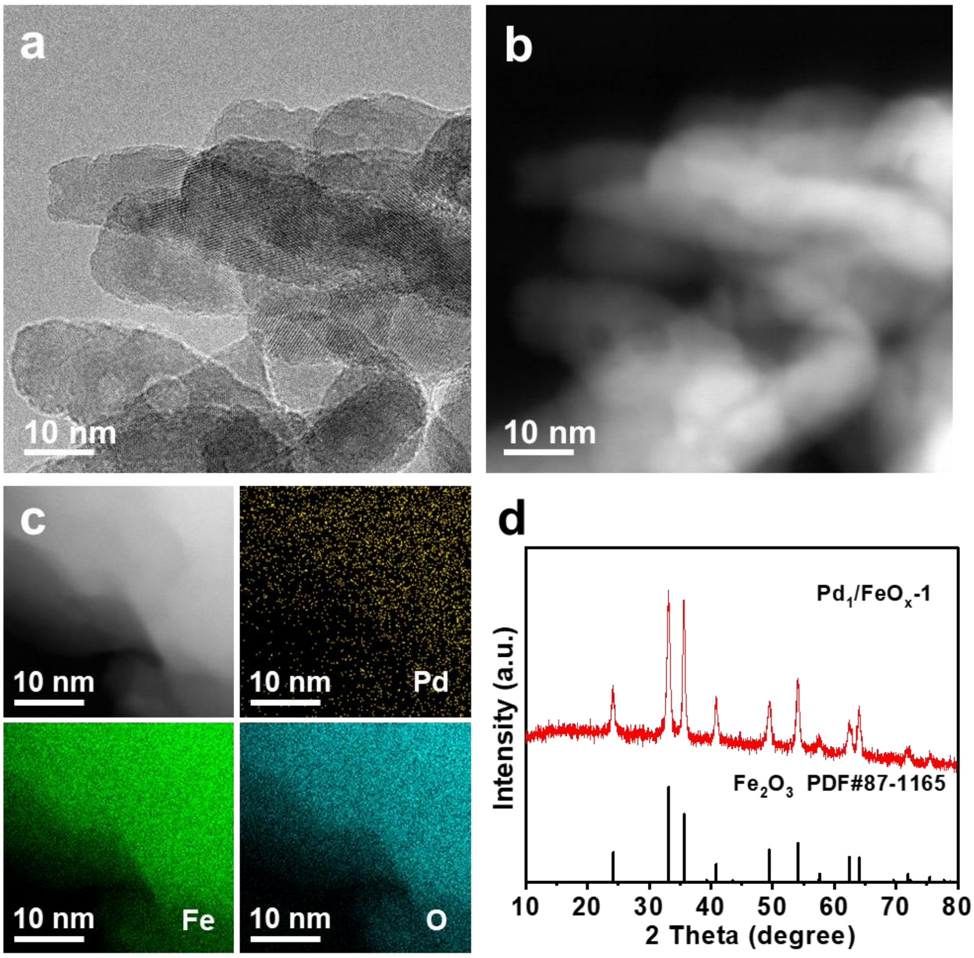

Supplementary Fig. 73 | Structural characterization results of Pd_1/FeO_x-1. a TEM image, **b** STEM image, **c** elemental mapping, and **d** XRD pattern of Pd_1/FeO_x-1. Scale bar, 10 nm.

Supplementary Table 47. Pd content and BET surface area for Pd_1/FeO_x-1

Sample	Pd (wt%) [a]	BET surface area (m^2 g^{-1}) [b]
Pd_1/FeO_x-1	0.22	44.3

[a] ICP-OES. [b] Sorption isotherm of N_2 at 77 K.

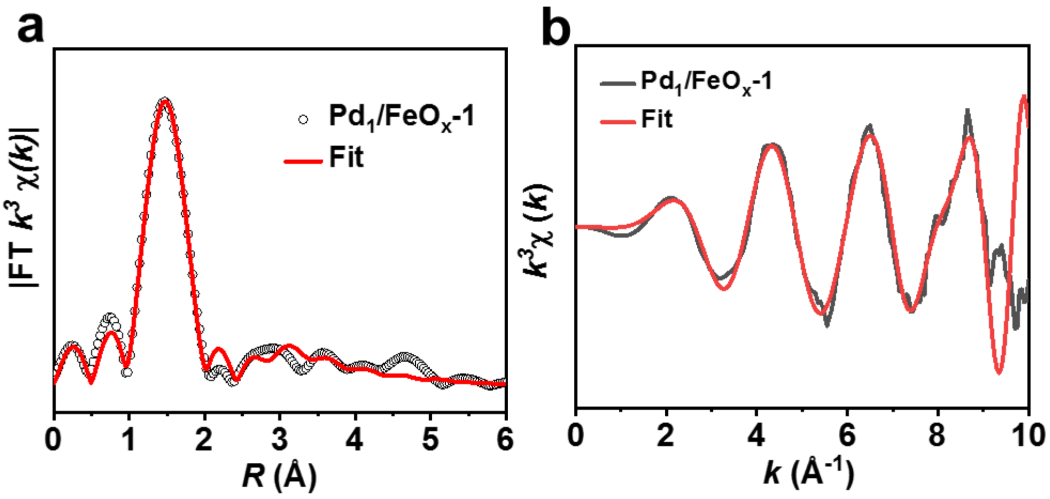

Supplementary Fig. 74 | EXAFS fitting for the Pd_1/FeO_x-1. a Fourier transform (FT) k^3-weighted $\chi(k)$-function of the EXAFS spectra for Pd K-edge and corresponding R-space fitting curves for the Pd_1/FeO_x-1 catalyst. **b** EXAFS *k* space fitting curve and the experimental one of Pd_1/FeO_x-1.

Supplementary Table 48. Structural parameters of EXAFS fitting for the Pd_1/FeO_x-1

Sample	Scattering pair	CN [a]	R (Å) [b]	σ^2 ($\times 10^{-3}$ Å^2) [c]	ΔE_0 (eV) [d]	R factor [e]
Pd_1/FeO_x-1	Pd-O	4.0±0.5	2.04±0.01	2.6±1.3	-0.1±1.4	0.01
	Pd-O-Fe	0.2±0.4	3.39±0.02	24.9±14.2		

[a] CN is the coordination number; [b] R is interatomic distance (the bond length between central atoms and surrounding coordination atoms); [c] σ^2 is Debye-Waller factor (a measure of thermal and static disorder in absorber-scatterer distances); [d] ΔE_0 is edge-energy shift (the difference between the zero kinetic energy value of the sample and that of the theoretical model). [e] R factor is used to value the goodness of the fitting. Data range: $2.6 \leq k \leq 9.1$ Å^{-1}, $1.0 \leq R \leq 4.2$ Å.

Pd_1/FeO_x-2

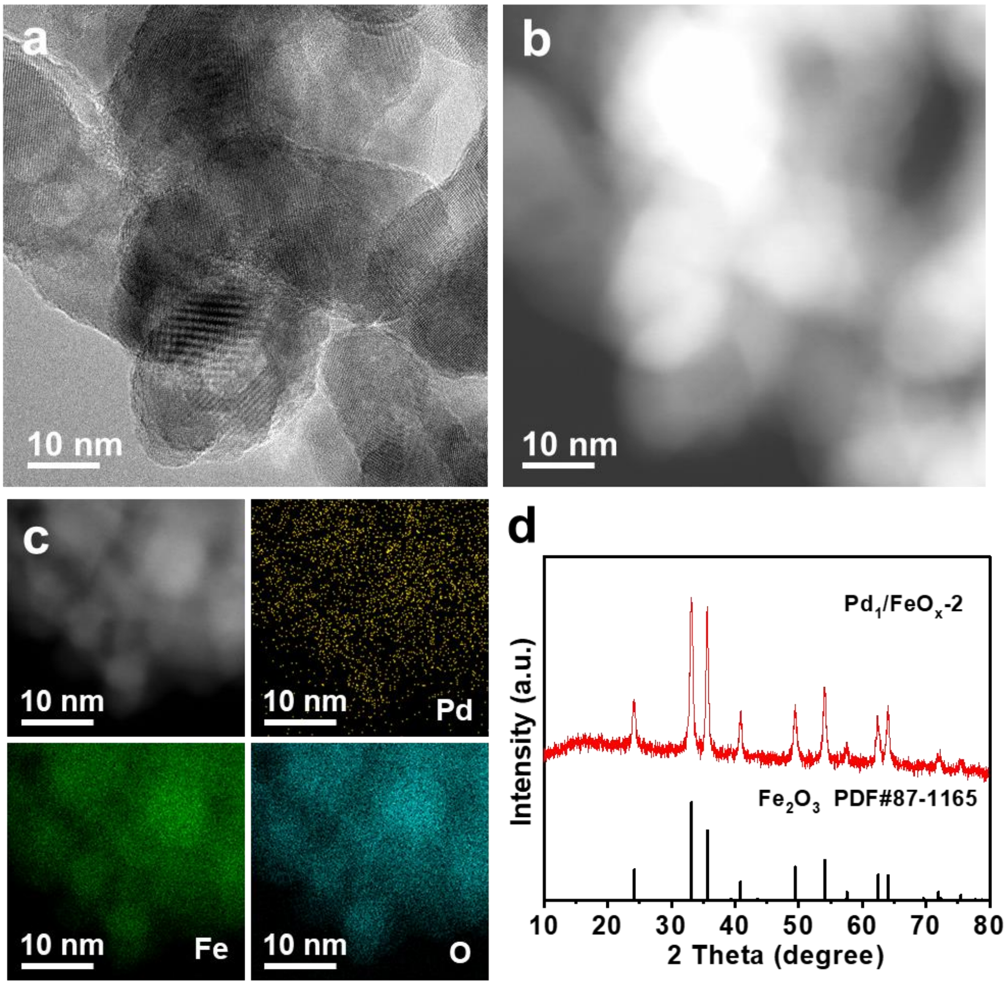

Supplementary Fig. 75 | Structural characterization results of Pd_1/FeO_x-2. **a** TEM image, **b** STEM image, **c** elemental mapping, and **d** XRD pattern of Pd_1/FeO_x-2. Scale bar, 10 nm.

Supplementary Table 49. Pd content and BET surface area for Pd_1/FeO_x-2

Sample	Pd (wt%) [a]	BET surface area (m^2 g^{-1}) [b]
Pd_1/FeO_x-2	0.22	42.1

[a] ICP-OES. [b] Sorption isotherm of N_2 at 77 K.

Pd_1/FeO_x-3

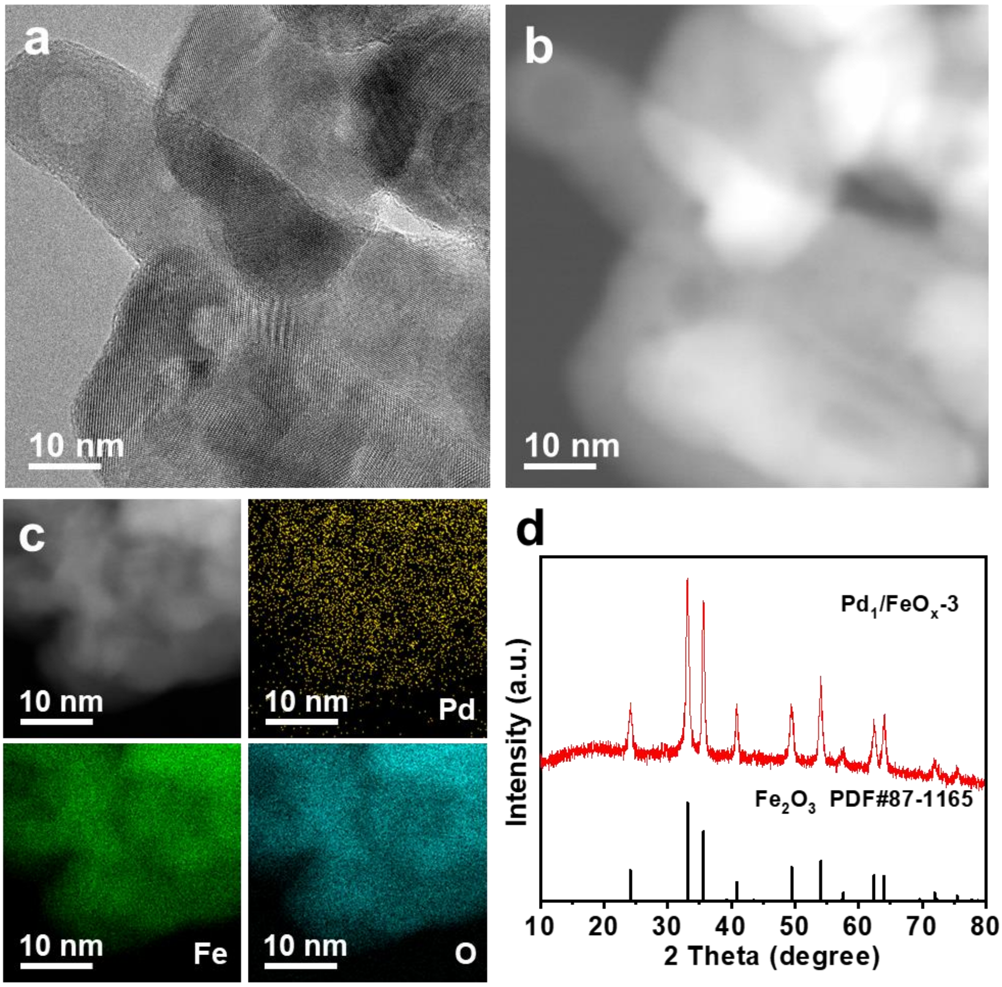

Supplementary Fig. 76 | Structural characterization results of Pd_1/FeO_x-3. a TEM image, **b** STEM image, **c** elemental mapping, and **d** XRD pattern of Pd_1/FeO_x-3. Scale bar, 10 nm.

Supplementary Table 50. Pd content and BET surface area for Pd_1/FeO_x-3

Sample	Pd (wt%) [a]	BET surface area ($m^2\ g^{-1}$) [b]
Pd_1/FeO_x-3	0.24	42.8

[a] ICP-OES. [b] Sorption isotherm of N_2 at 77 K.

Pd_1/FeO_x-4

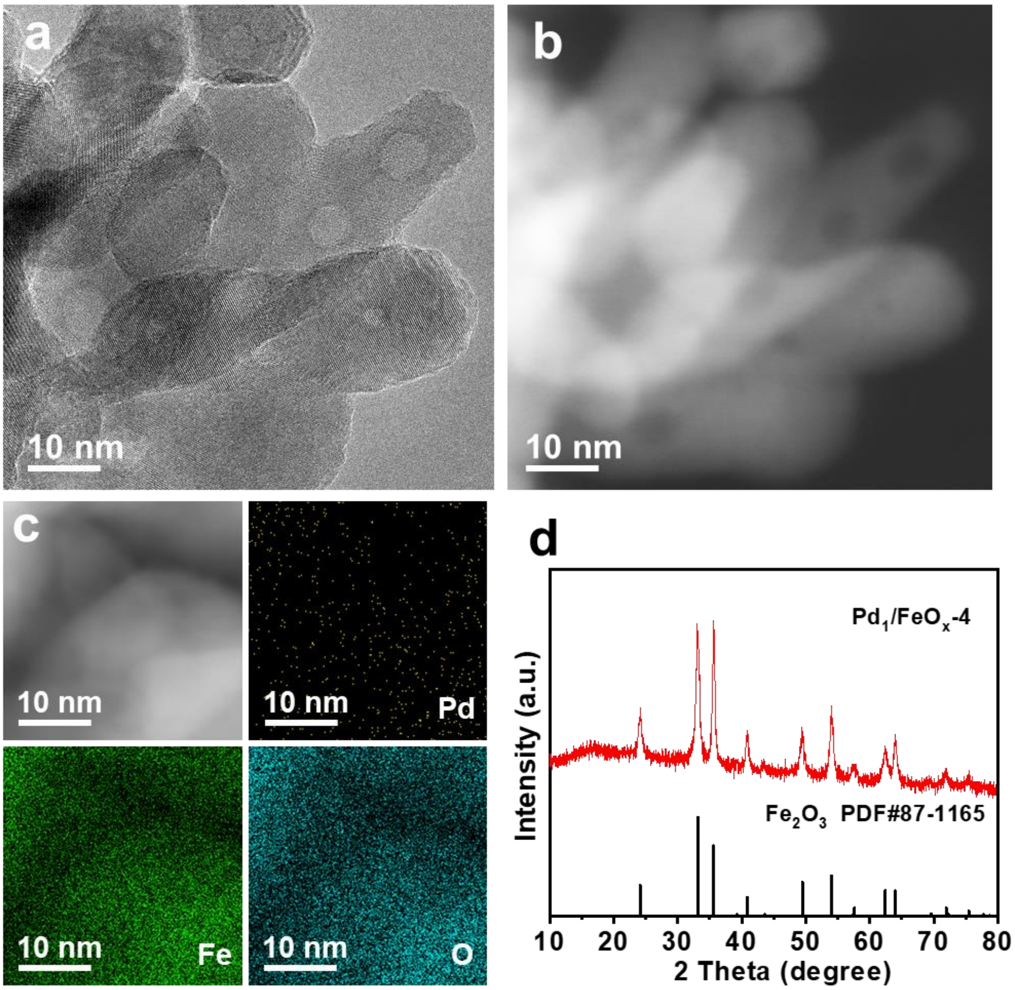

Supplementary Fig. 77 | Structural characterization results of Pd_1/FeO_x-4. **a** TEM image, **b** STEM image, **c** elemental mapping, and **d** XRD pattern of Pd_1/FeO_x-4. Scale bar, 10 nm.

Supplementary Table 51. Pd content and BET surface area for Pd_1/FeO_x-4

Sample	Pd (wt%) [a]	BET surface area (m^2 g^{-1}) [b]
Pd_1/FeO_x-4	0.24	47.2

[a] ICP-OES. [b] Sorption isotherm of N_2 at 77 K.

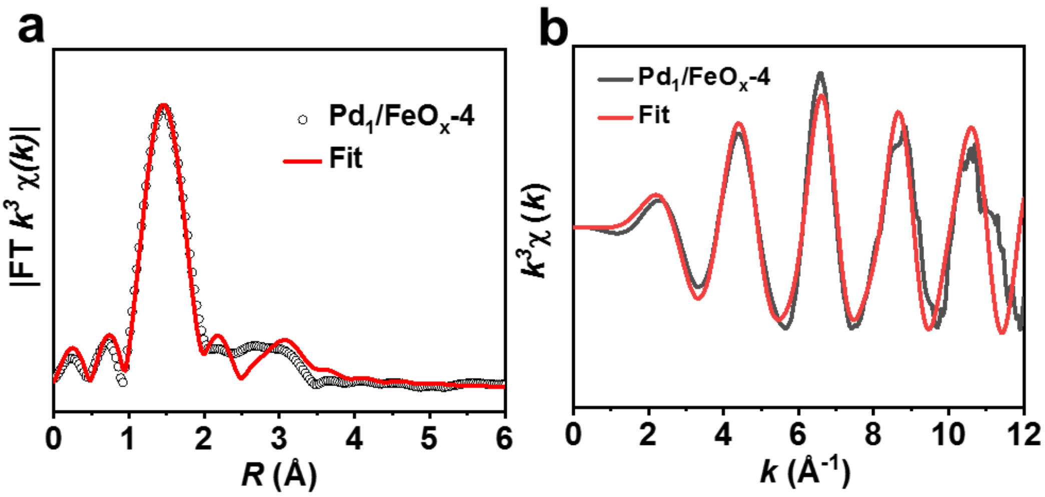

Supplementary Fig. 78 | EXAFS fitting for the Pd_1/FeO_x-4. a FT k^3-weighted χ(k)-function of the EXAFS spectra for Pd K-edge and corresponding R-space fitting curves for the Pd_1/FeO_x-4 catalyst. **b** EXAFS k space fitting curve and the experimental one of Pd_1/FeO_x-4.

Supplementary Table 52. Structural parameters of EXAFS fitting for the Pd_1/FeO_x-4

Sample	Scattering pair	CN [a]	R (Å) [b]	σ^2 (× 10^{-3} $Å^2$) [c]	ΔE_0 (eV) [d]	R factor [e]
Pd_1/FeO_x-4	Pd-O	3.7±0.7	2.01±0.01	1.2±2.0	-0.7±2.3	0.03
	Pd-O-Fe	1.5±2.4	3.42±0.03	6.3±14.3		

[a] CN is the coordination number; [b] R is interatomic distance (the bond length between central atoms and surrounding coordination atoms); [c] σ^2 is Debye-Waller factor (a measure of thermal and static disorder in absorber-scatterer distances); [d] ΔE_0 is edge-energy shift (the difference between the zero kinetic energy value of the sample and that of the theoretical model). [e] R factor is used to value the goodness of the fitting. Data range: $2.5 \leq k \leq 9.0$ $Å^{-1}$, $1.0 \leq R \leq 4.2$ Å.

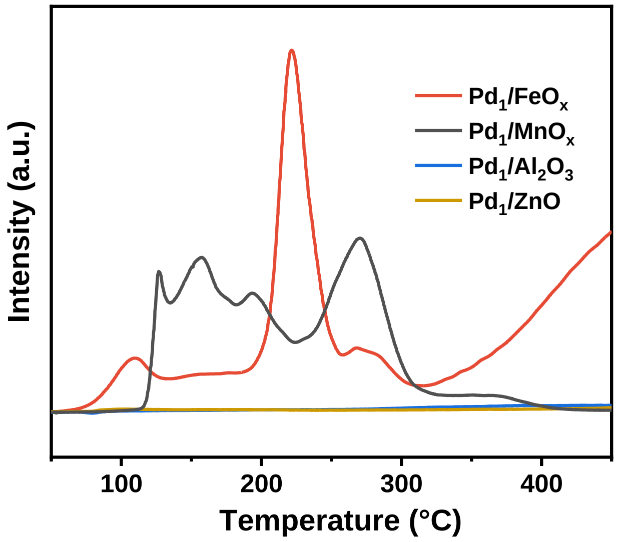

Supplementary Fig. 79 | H_2-TPR results. H_2-TPR results for Pd_1/FeO_x-1, Pd_1/MnO_x, Pd_1/ZnO, and Pd_1/Al_2O_3. Measurement conditions: 30 mg catalyst, 30 mL/min 5% H_2/N_2, room temperature to 500 ^oC with 10 ^oC/min rate.

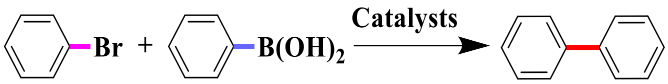

Supplementary Table 53. Comparison of the activities of various Pd catalysts in Suzuki-Miyaura cross-coupling

Entry	Catalyst	n_{Pd}(mol%)	n_{C6H5Br} (mmol)	Temp. (°C)	TOF (h^{-1})	Ref.
1	Pd_1/FeO_x-1	0.0226	0.5	30	9493	This work
2	Pd_1/FeO_x-1	0.0226	0.5	40	23810	This work
3	Pd_1/FeO_x-1	0.0240	0.5	50	51944	This work
4	PdNC/NRGO	0.0140	1	R.T.	1642	1
5	Magnetite@MCM-41@NHC@Pd	0.0226	0.5	R.T.	8867	2
6	Pd_1-$Ti_{0.87}O_2$	0.0470	0.1	R.T.	11110[a]	3
7	Pd–$Co_3[Co(CN)_6]_2$	0.4500	1	50	111	4
8	Fe_3O_4@SiO_2-TCT-GA-Pd(0)	0.12	1	50	533	5
9	Fe_3O_4/CS-Me@Pd	0.0026	1	50	74000	6
10	Mag-IL-Pd	0.025	0.5	60	437	7
11	Y_3Pd_2	40	0.5	60	4034	8
12	Fe_3O_4dpa@$Pd_{0.5}$	0.04	0.5	65	3150	9
13	PdO/Ce_xO_y	0.007	2	70	13944	10
14	Pd/SMP-$PhPh_3$	0.05	0.5	80	61353	11
15	Fe_3O_4@SiO_2@NHC@Pd-MNPs	0.0079	0.5	80	37691	12
16	Pd/$Ni(OH)_2$/SS	0.17	1	90	25976	13

[a] The substrate is p-bromotoluene instead of bromobenzene.

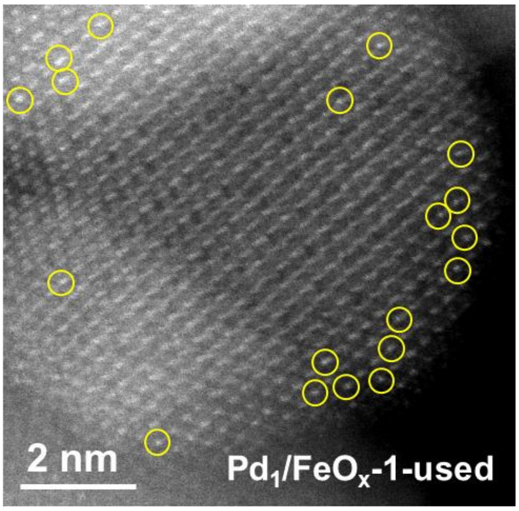

Supplementary Fig. 80 | Structural characterization results of Pd_1/FeO_x-1-used.

AC HAADF-STEM image of Pd_1/FeO_x-1-used.

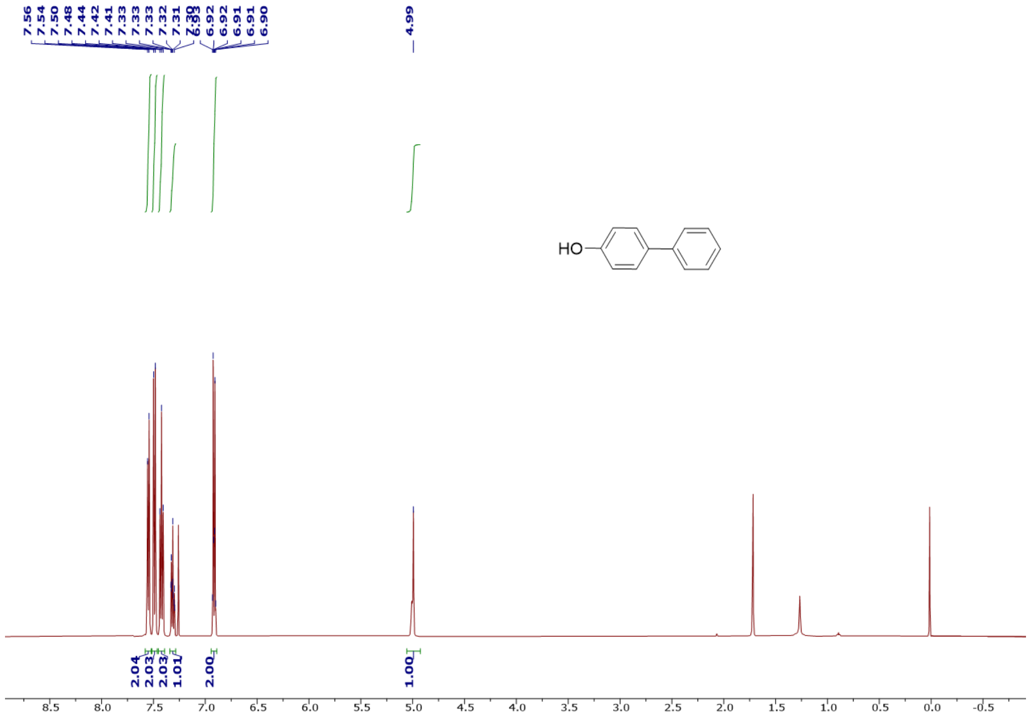

Supplementary Fig. 81 | NMR spectrum. ^{1}H NMR spectrum of 4-Phenylphenol (Solvent: $CDCl_3$).

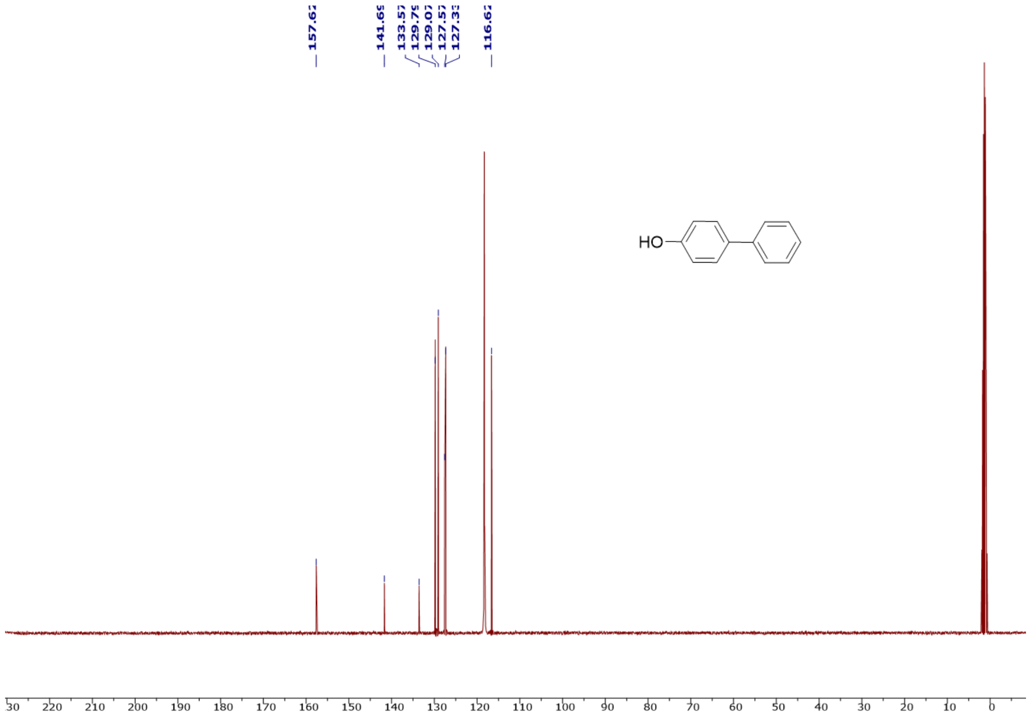

Supplementary Fig. 82 | NMR spectrum. ^{13}C NMR spectrum of 4-Phenylphenol (Solvent: CD_3CN).

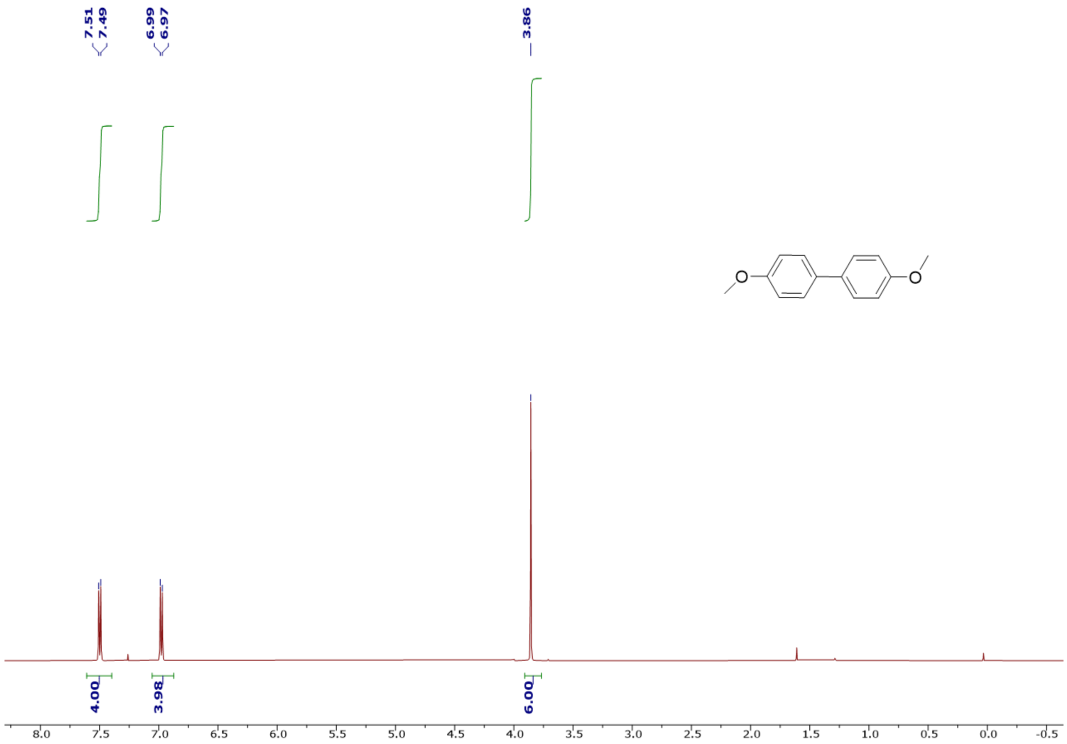

Supplementary Fig. 83 | NMR spectrum. ^{1}H NMR spectrum of 4,4'-Dimethoxybiphenyl (Solvent: $CDCl_3$).

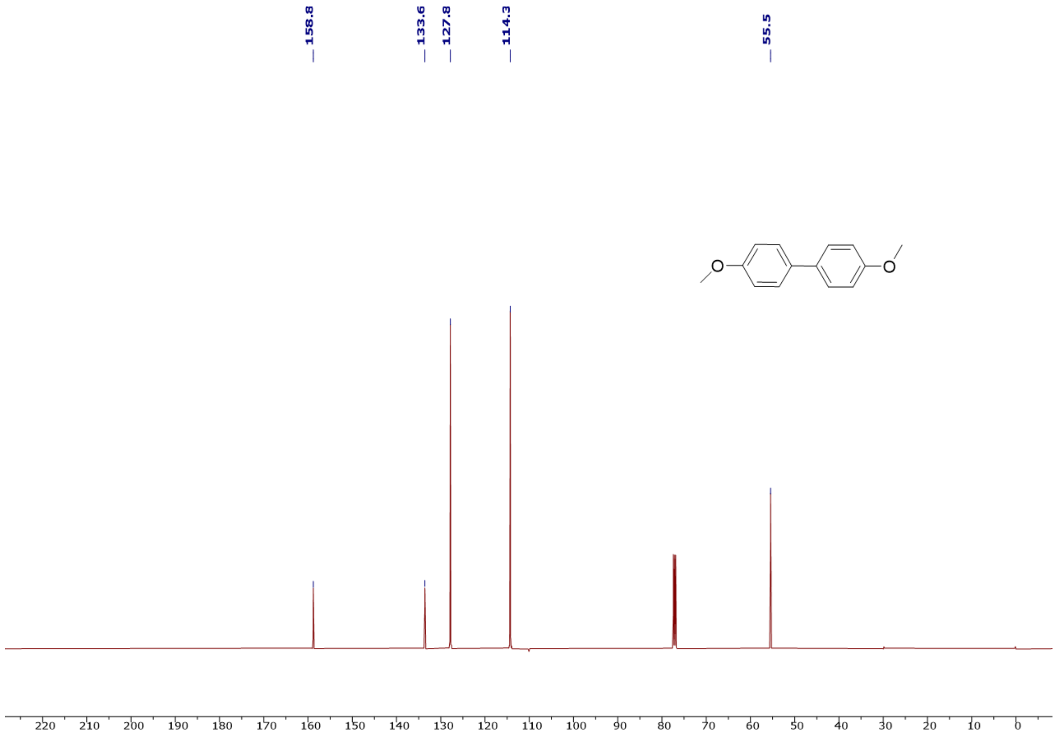

Supplementary Fig. 84 | NMR spectrum. ^{13}C NMR spectrum of 4,4'-Dimethoxybiphenyl (Solvent: $CDCl_3$).

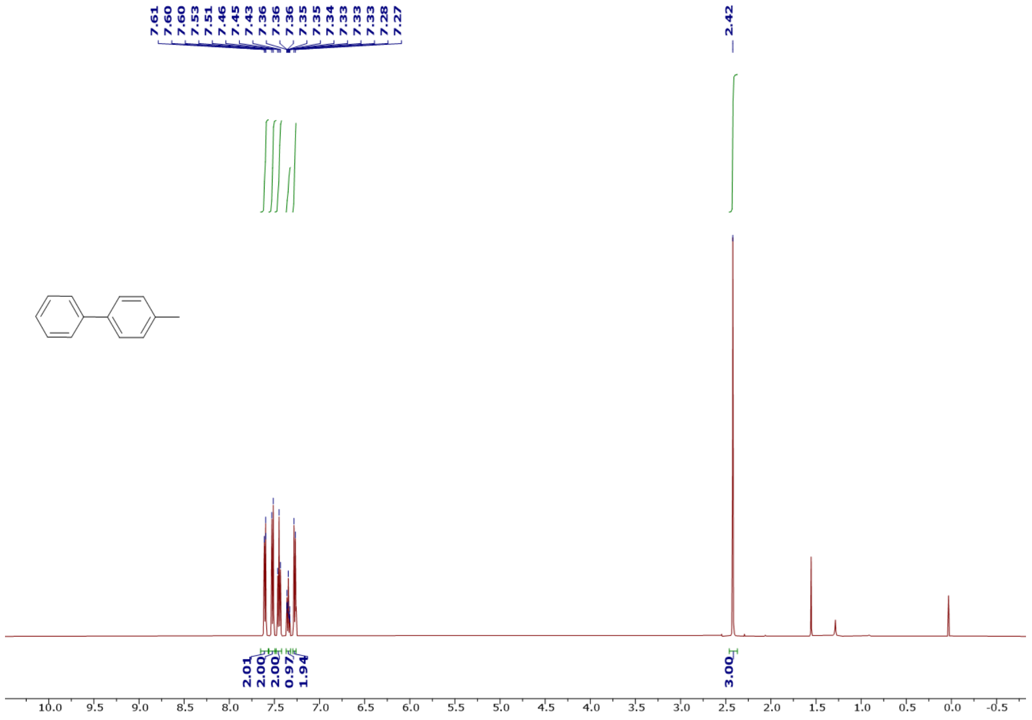

Supplementary Fig. 85 | NMR spectrum. ^{1}H NMR spectrum of 4-Phenyltoluene (Solvent: $CDCl_3$).

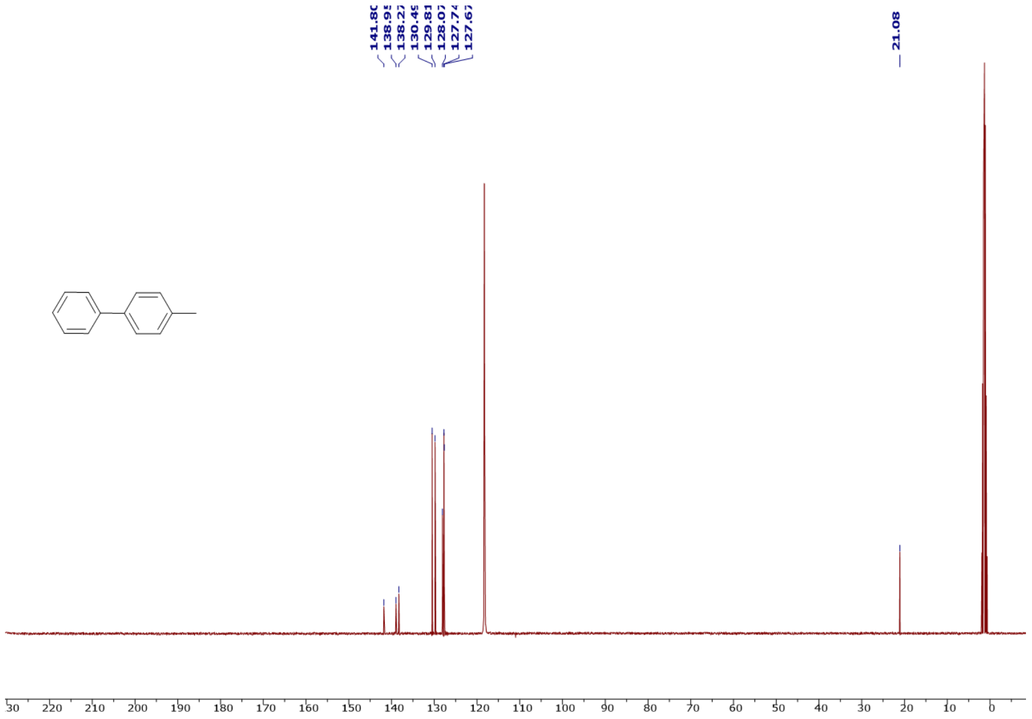

Supplementary Fig. 86 | NMR spectrum. ^{13}C NMR spectrum of 4-Phenyltoluene (Solvent: CD_3CN).

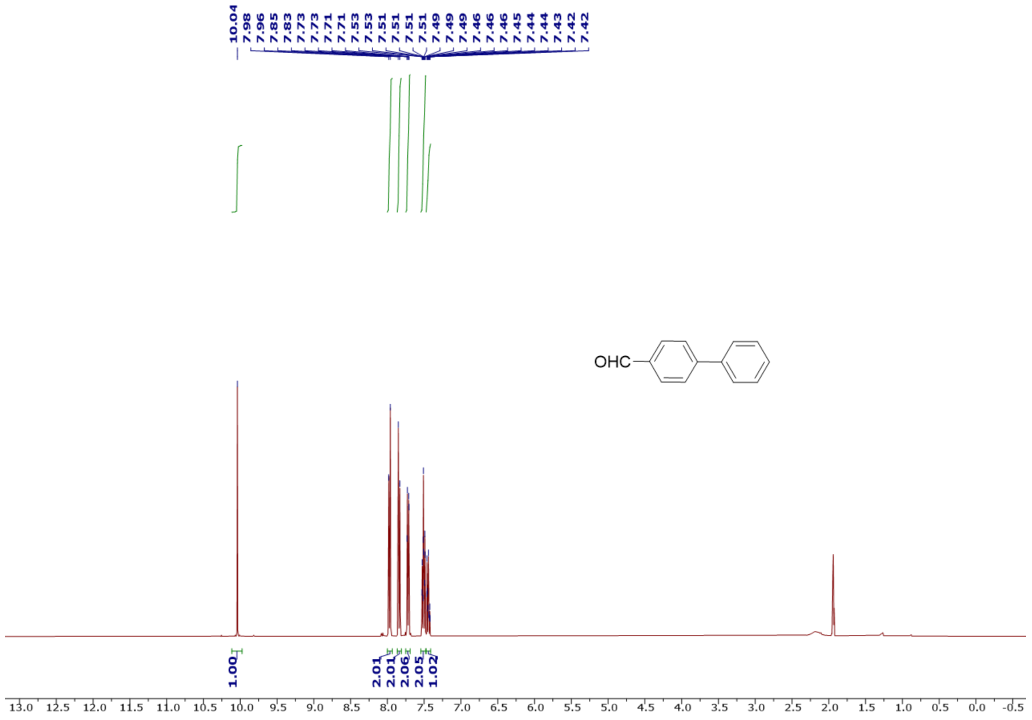

Supplementary Fig. 87 | NMR spectrum. ^{1}H NMR spectrum of 4-Phenylbenzaldehyde (Solvent: CD_3CN).

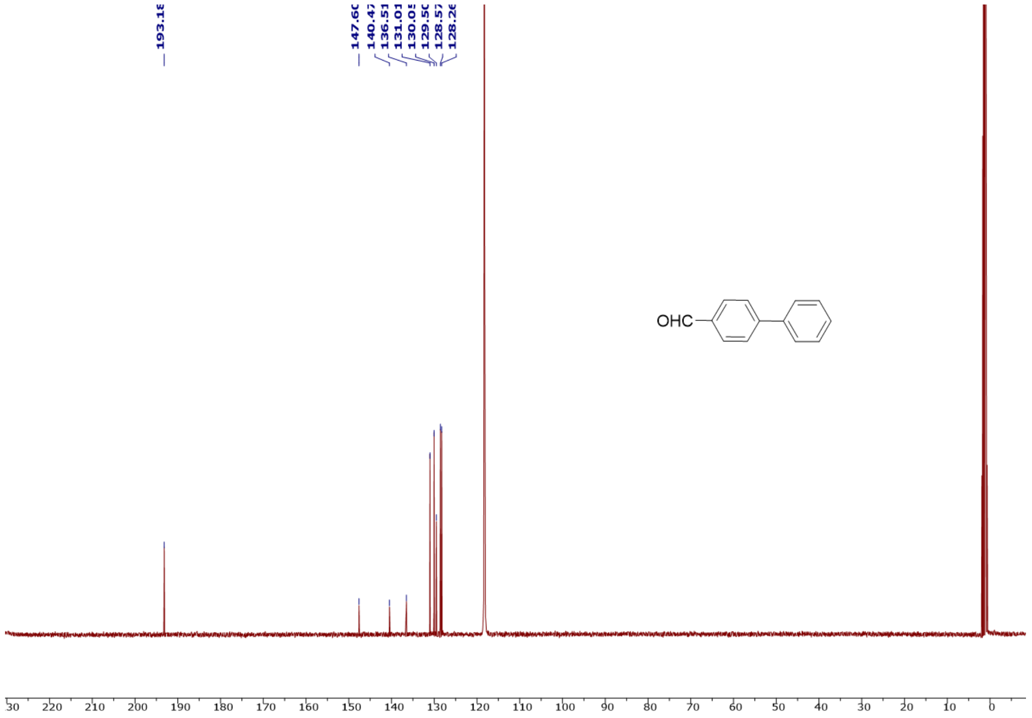

Supplementary Fig. 88 | NMR spectrum. ^{13}C NMR spectrum of 4-Phenylbenzaldehyde (Solvent: CD_3CN).

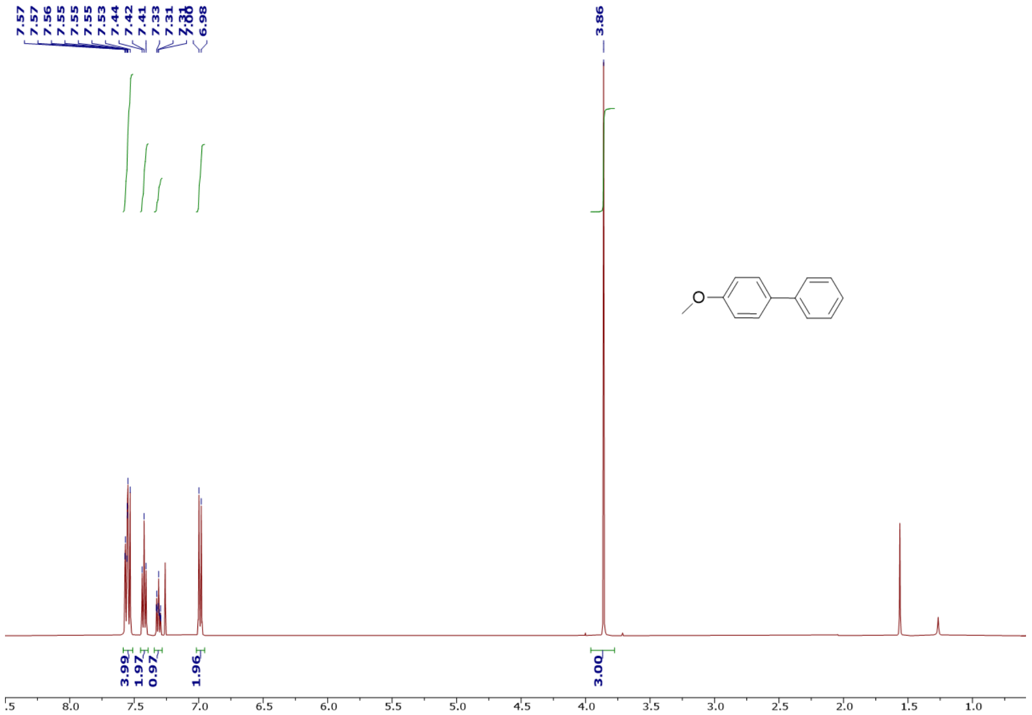

Supplementary Fig. 89 | NMR spectrum. ^{1}H NMR spectrum of 4-Methoxybiphenyl

(Solvent: $CDCl_3$).

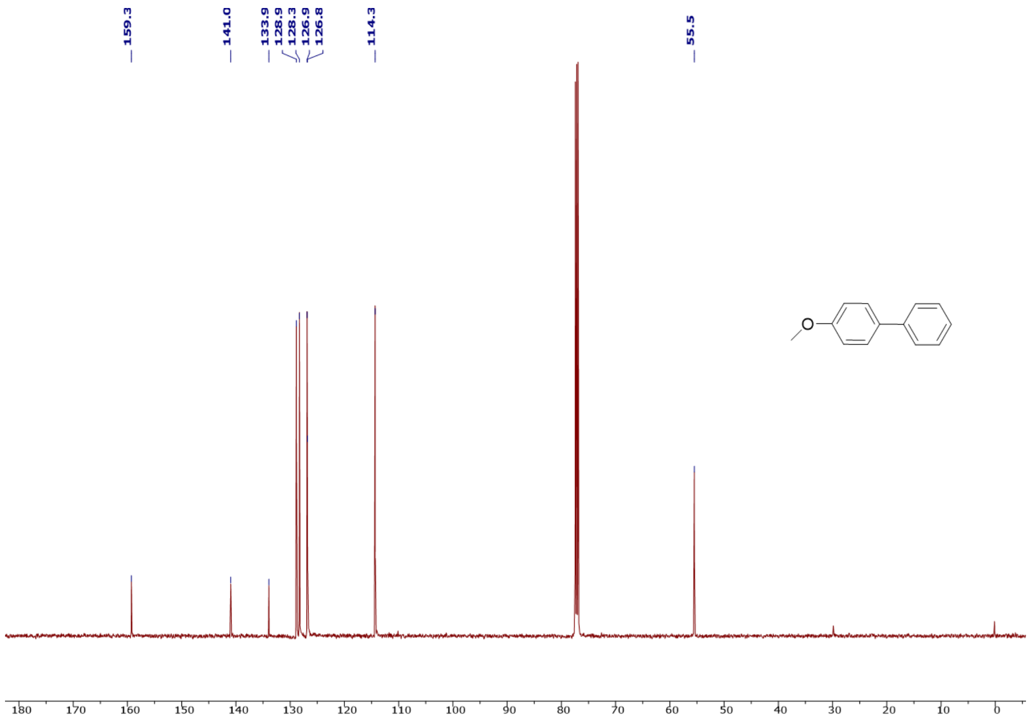

Supplementary Fig. 90 | NMR spectrum. ^{13}C NMR spectrum of 4-Methoxybiphenyl

(Solvent: $CDCl_3$).

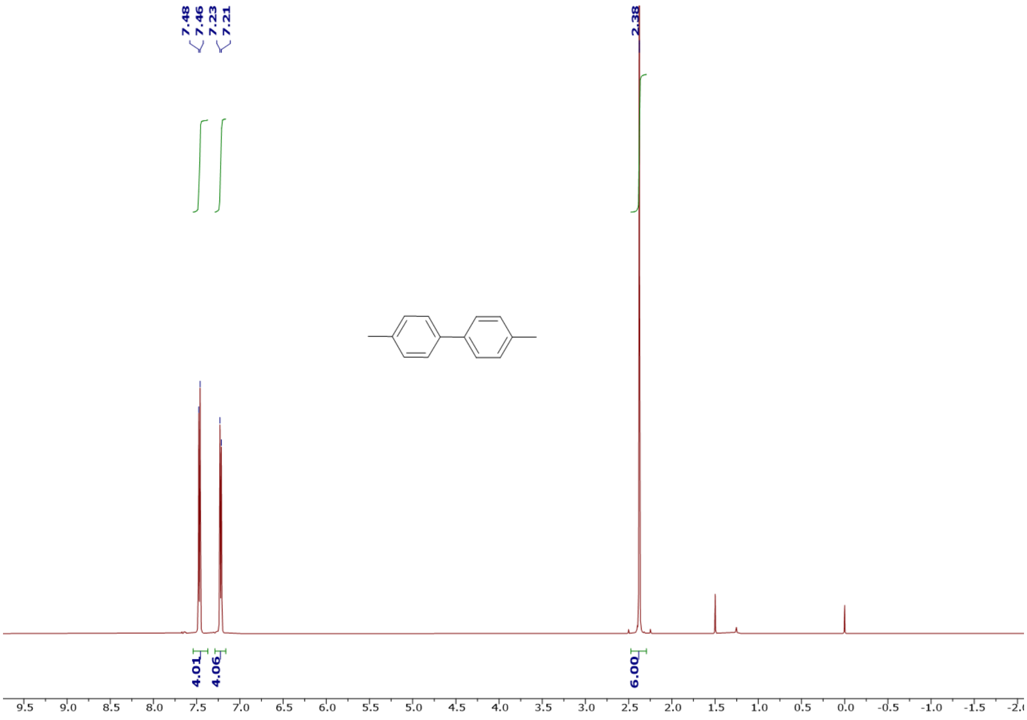

Supplementary Fig. 91 | NMR spectrum. ^{1}H NMR spectrum of 4,4'-Dimethylbiphenyl (Solvent: $CDCl_3$).

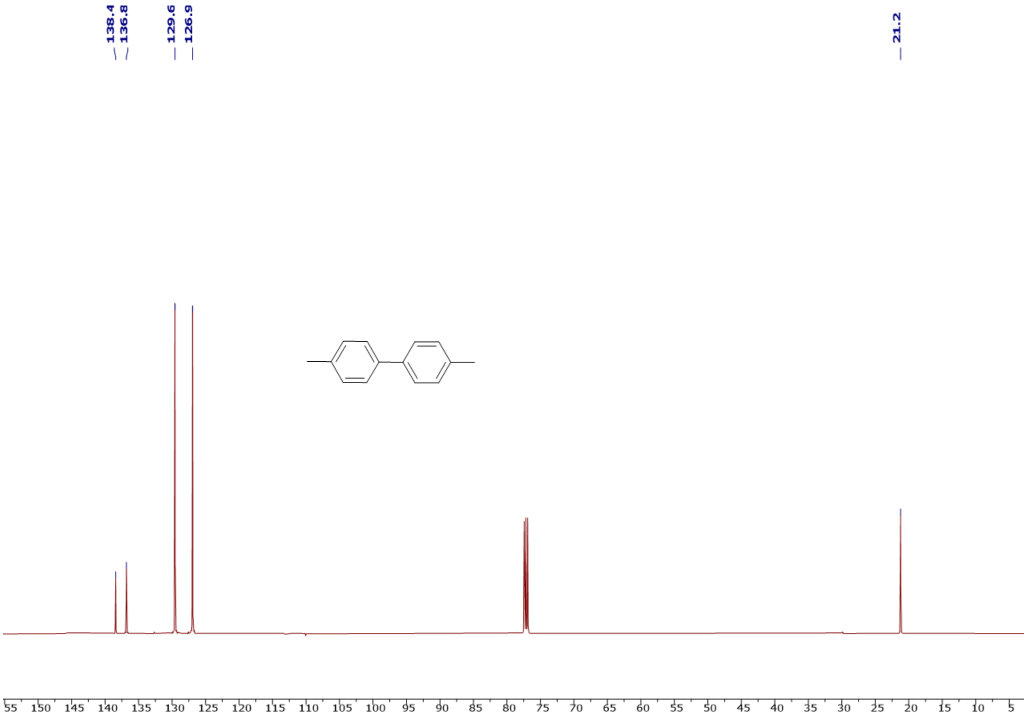

Supplementary Fig. 92 | NMR spectrum. ^{13}C NMR spectrum of 4,4'-Dimethylbiphenyl (Solvent: $CDCl_3$).

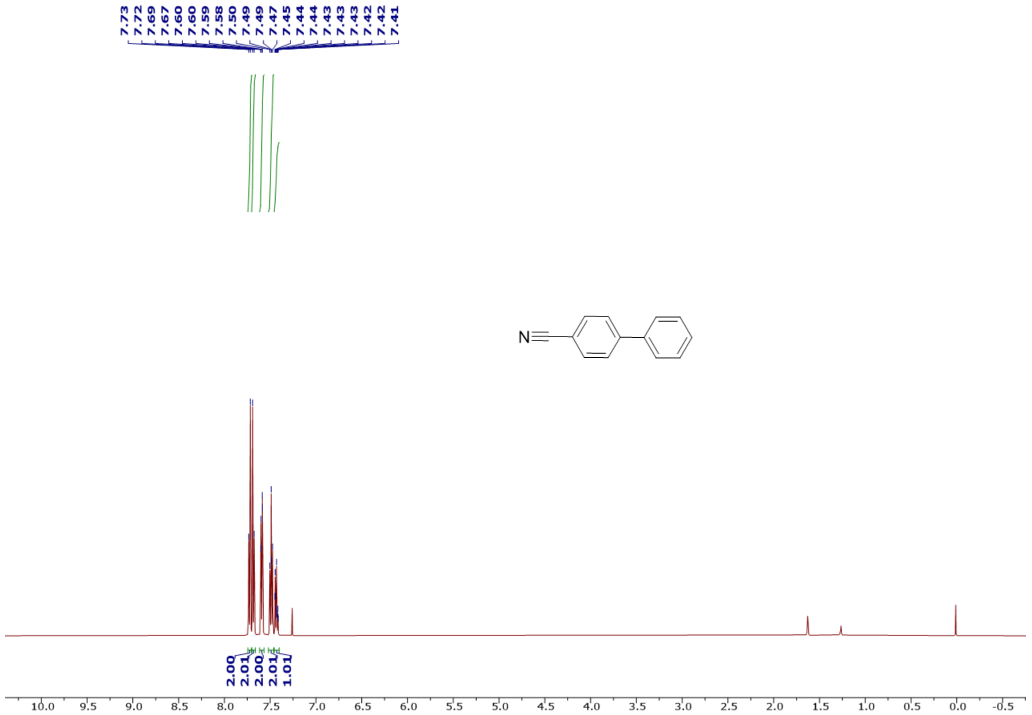

Supplementary Fig. 93 | NMR spectrum. ^{1}H NMR spectrum of 4-Phenylbenzonitrile (Solvent: $CDCl_3$).

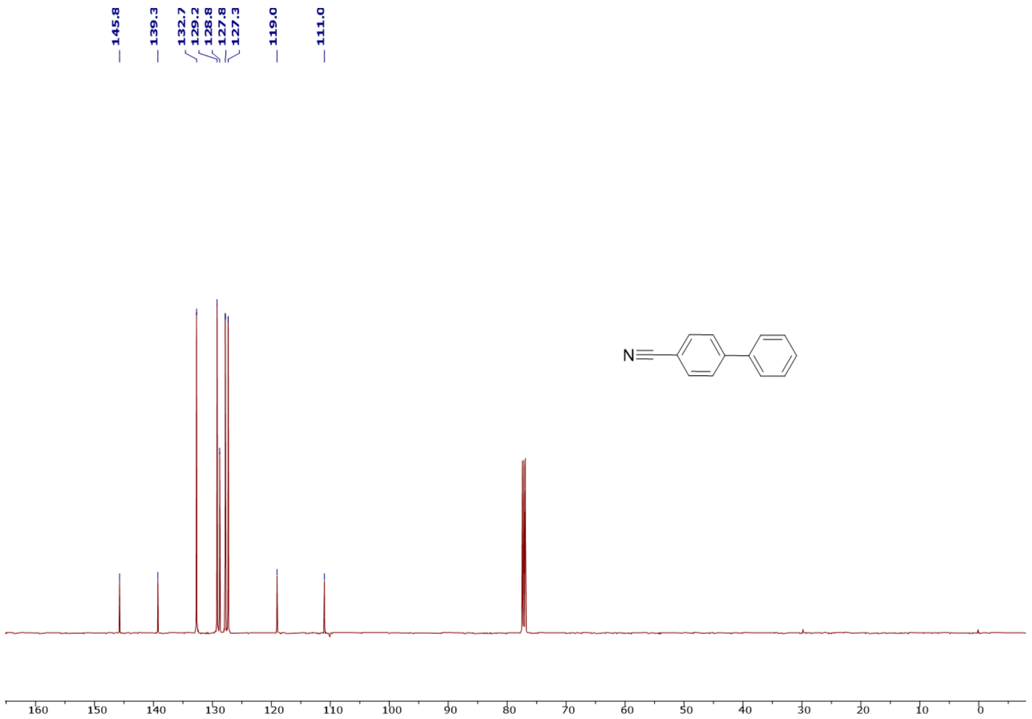

Supplementary Fig. 94 | NMR spectrum. ^{13}C NMR spectrum of 4-Phenylbenzonitrile (Solvent: $CDCl_3$).

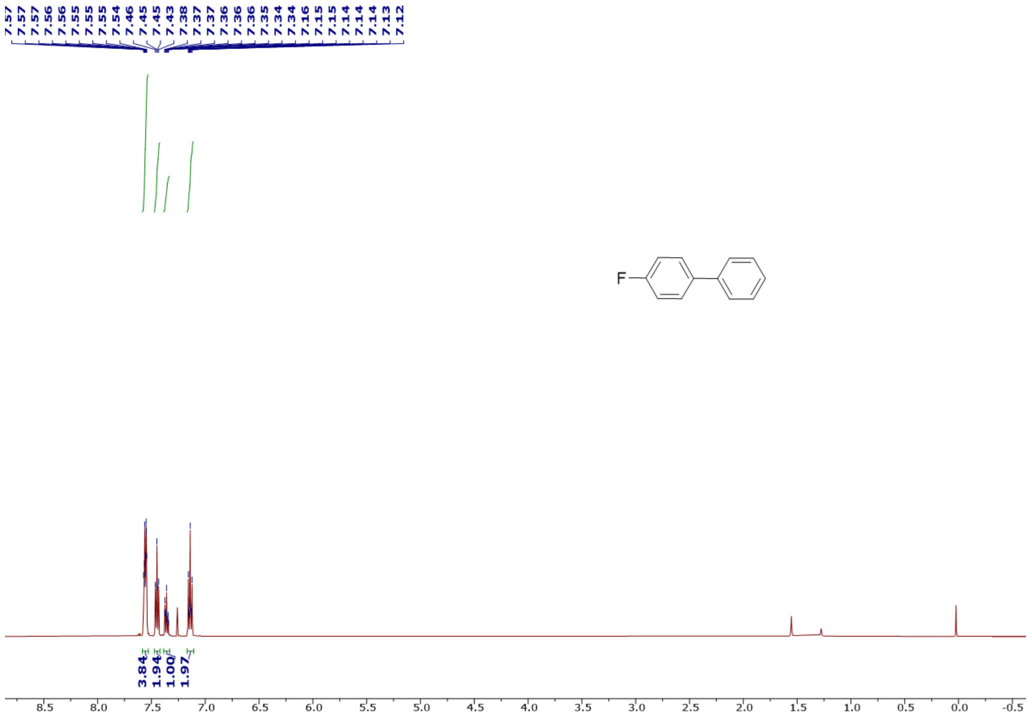

Supplementary Fig. 95 | NMR spectrum. ^{1}H NMR spectrum of 4-Fluorobiphenyl (Solvent: $CDCl_3$).

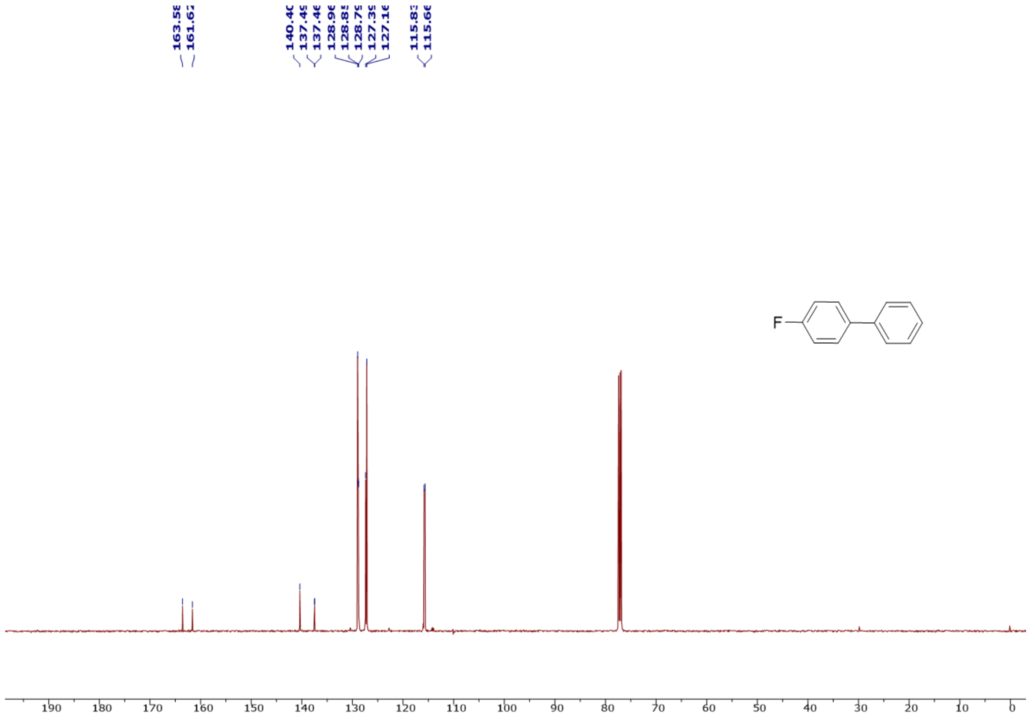

Supplementary Fig. 96 | NMR spectrum. ^{13}C NMR spectrum of 4-Fluorobiphenyl (Solvent: $CDCl_3$).

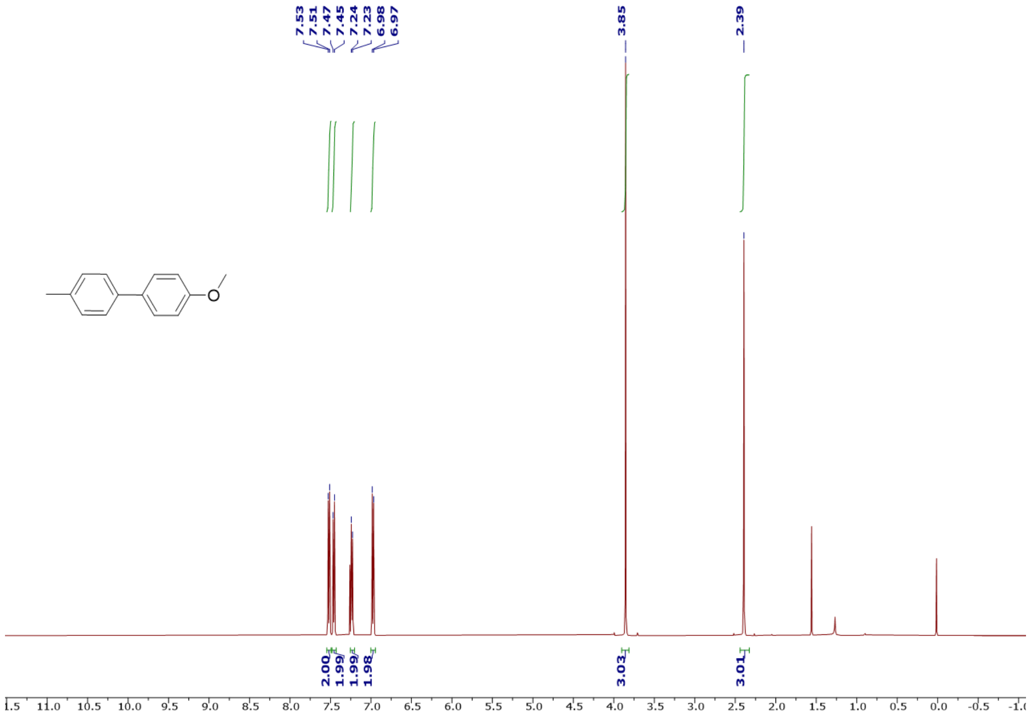

Supplementary Fig. 97 | NMR spectrum. ^{1}H NMR spectrum of 4-methoxy-4'-methylphenyl (Solvent: $CDCl_3$).

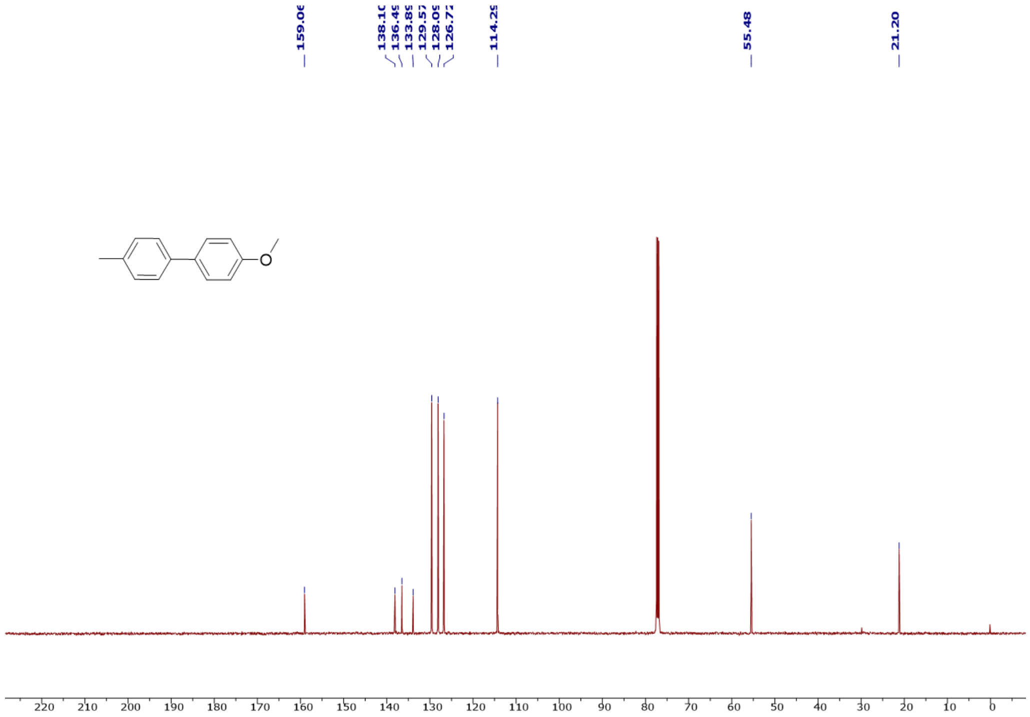

Supplementary Fig. 98 | NMR spectrum. ^{13}C NMR spectrum of 4-methoxy-4'-methylphenyl (Solvent: $CDCl_3$).

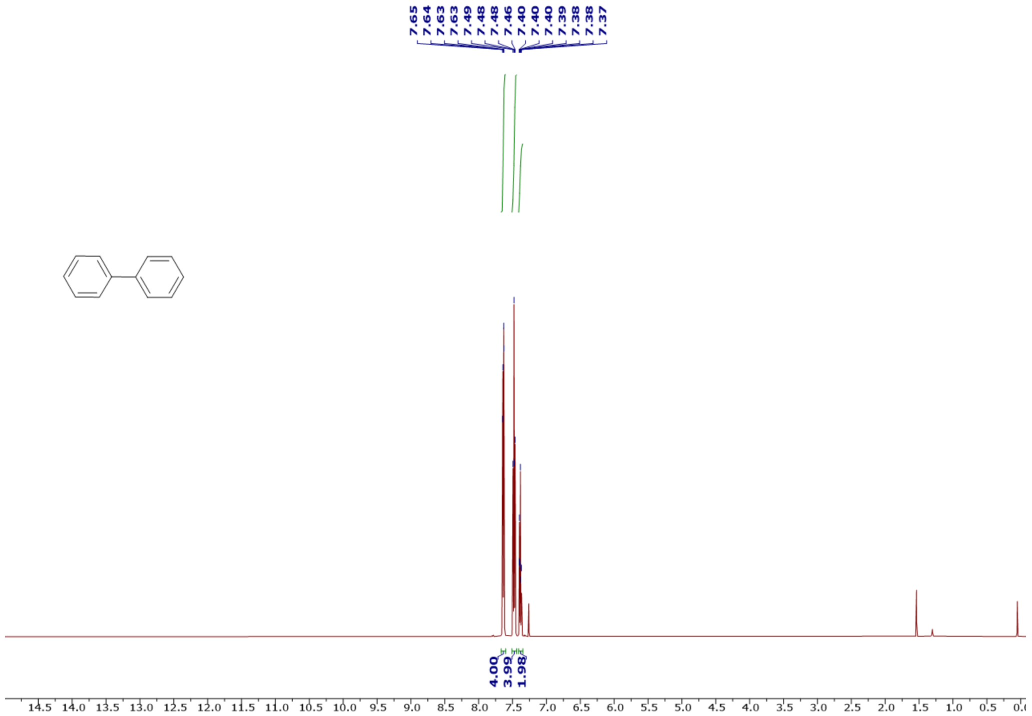

Supplementary Fig. 99 | NMR spectrum. ^{1}H NMR spectrum of Diphenyl (Solvent: $CDCl_3$).

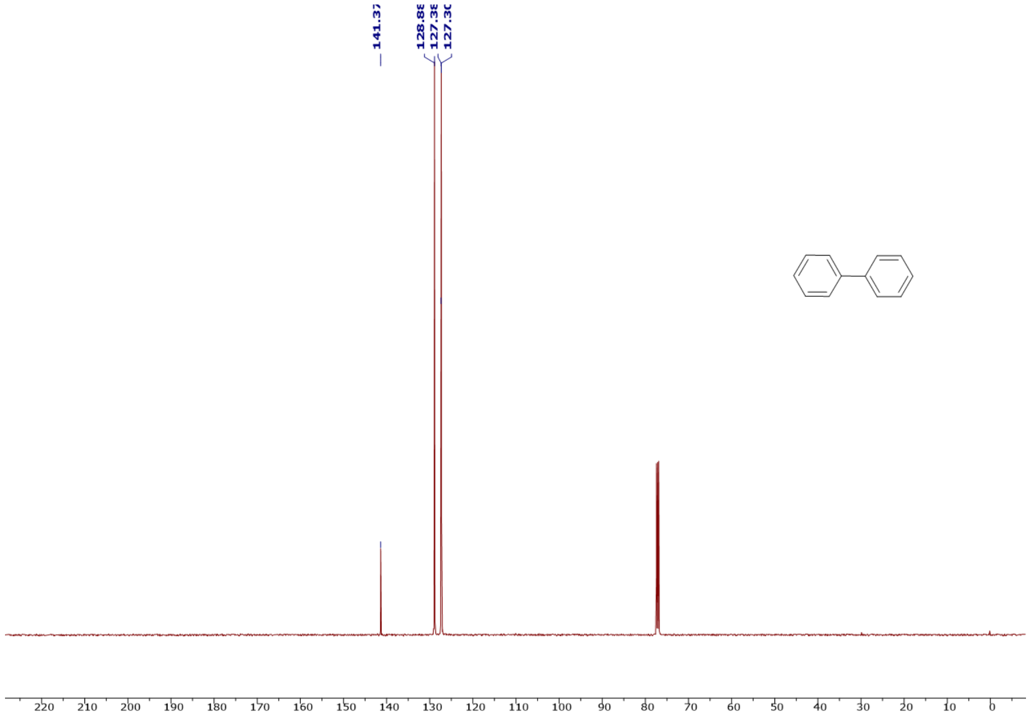

Supplementary Fig. 100 | NMR spectrum. ^{13}C NMR spectrum of Diphenyl (Solvent: $CDCl_3$).

Supplementary References

1. Sahoo, L., Mondal, S., Nayana, C.B. & Gautam, U.K. Facile d-band tailoring in Sub-10 nm Pd cubes by in-situ grafting on nitrogen-doped graphene for highly efficient organic transformations. *J. Colloid Interface Sci.* **590**, 175-185 (2021).
2. Akkoç, M., Buğday, N., Altın, S. & Yaşar, S. Magnetite@MCM-41 nanoparticles as support material for Pd-N-heterocyclic carbene complex: A magnetically separable catalyst for Suzuki-Miyaura reaction. *Appl. Organomet. Chem.* **35**, e6233 (2021).
3. Jin, Y., *et al.* Engineering electronic structure of single-atom Pd site on $Ti_{0.87}O_2$ nanosheet via charge transfer enables C–Br cleavage for room-temperature Suzuki coupling. *CCS Chem.* **3**, 1453-1462 (2021).
4. Li, R., Li, R., Wang, C., Gao, L. & Chen, Q. Pd-$Co_3[Co(CN)_6]_2$ hybrid nanoparticles: preparation, characterization, and challenge for the Suzuki-Miyaura coupling of aryl chlorides under mild conditions. *Dalton Trans.* **45**, 539-544 (2016).
5. Eslahi, H., Sardarian, A.R. & Esmaeilpour, M. Green and sustainable palladium nanomagnetic catalyst stabilized by glucosamine-functionalized Fe_3O_4@SiO_2 nanoparticles for Suzuki and Heck reactions. *Appl. Organomet. Chem.* **35**, e6260 (2021).
6. Wang, G., Lv, K., Chen, T., Chen, Z. & Hu, J. Immobilizing of palladium on melamine functionalized magnetic chitosan beads: A versatile catalyst for p-nitrophenol reduction and Suzuki reaction in aqueous medium. *Int. J. Biol. Macromol.* **184**, 358-368 (2021).
7. Karimi, B., Mansouri, F. & Vali, H. A highly water-dispersible/magnetically separable palladium catalyst based on a Fe_3O_4@SiO_2 anchored TEG-imidazolium ionic liquid for the Suzuki–Miyaura coupling reaction in water. *Green Chem.* **16**, 2587–2596 (2014).
8. Ye, T.N., *et al.* Palladium-bearing intermetallic electride as an efficient and stable catalyst for Suzuki cross-coupling reactions. *Nat. Commun.* **10**, 5653 (2019).
9. Guarnizo, A., *et al.* Highly water-dispersible magnetite-supported Pd nanoparticles and single atoms as excellent catalysts for Suzuki and hydrogenation reactions. *RSC Adv.* **6**, 68675-68684 (2016).
10. Yu, D., Jie, B., Wang, J. & Li, C. Design and fabrication of PdO/Ce_xO_y composite catalysts with coaxial nanotuber and studies of their synergistic performance in Suzuki-Miyaura reactions. *J. Catal.* **365**, 195-203 (2018).
11. Song, K., Liu, P., Wang, J., Tan, B. & Li, T. Highly active palladium nanoparticles immobilized on knitting microporous organic polymers as efficient catalysts for Suzuki–Miyaura cross-coupling reaction. *J. Porous Mater.* **23**, 725-731 (2016).

12. Akkoç, M., *et al.* N-heterocyclic carbene Pd(II) complex supported on Fe_3O_4@SiO_2: Highly active, reusable and magnetically separable catalyst for Suzuki-Miyaura cross-coupling reactions in aqueous media. *J. Organomet. Chem.* **943,** 121823 (2021).
13. Sun, X., *et al.* A hierarchical-structured impeller with engineered Pd nanoparticles catalyzing Suzuki coupling reactions for high-purity biphenyl. *ACS Appl. Mater. Inter.* **13**, 17429-17438 (2021).
