# Supplementary material for: Building up libraries and production line for single atom catalysts with precursor-atomization strategy
Source: Nat Commun. 2022 Sep 29;13:5721. doi: 10.1038/s41467-022-33442-2 (PMC9522824; doi:10.1038/s41467-022-33442-2)
Supplement: Supplementary file 3 — Description of Additional Supplementary Files [file 41467_2022_33442_MOESM3_ESM.pdf]

Description of Additional Supplementary Files

File Name: Supplementary Movie 1
Description: Continuous synthesis of SACs on our production line.
